# A Comprehensive, Affordable, Open-Source Hardware-Software Solution for Flexible Implementation of Complex Behaviors in Head-Fixed Mice

**DOI:** 10.1523/ENEURO.0018-23.2023

**Published:** 2023-06-26

**Authors:** Ali Ozgur, Soo Bin Park, Abigail Yap Flores, Mikko Oijala, Gyorgy Lur

**Affiliations:** University of California Irvine, Irvine, California 92697

**Keywords:** Go-NoGo, hardware-software, head-fixed behavior, open-source, sensory perception, two-alternative forced choice

## Abstract

Experiments that take advantage of head-fixed behavioral tasks have been a staple of systems neuroscience research for half a century. More recently, rodents came to the forefront of these efforts, primarily because of the rich experimental possibilities afforded by modern genetic tools. There is, however, a considerable barrier to entering this field, requiring expertise in engineering, hardware and software development, and significant time and financial commitment. Here, we present a comprehensive, open-source hardware and software solution to implement a head-fixed environment for rodent behaviors (HERBs). Our solution provides access to three frequently used experimental frameworks (two-alternative forced choice, Go-NoGo, or passive sensory stimulus presentation) in a single package. The required hardware can be built at a relatively low cost compared with commercially available solutions, from off-the-shelf components. Our graphical user interface-based software provides great experimental flexibility and requires no programming experience for either installation or use. Furthermore, an HERBs takes advantage of motorized components that allow the precise, temporal separation of behavioral phases (stimulus presentation, delays, response window and reward). Overall, we present a solution that will allow laboratories to join the growing community of systems neuroscience research at a substantially lower cost of entry.

## Significance Statement

In the past 2 decades, head-fixed rodent preparations have become an invaluable tool in systems neuroscience. Still, setting up sensory perception or complex behavioral experiments remains an arduous task, requiring expertise in hardware and software development, as well as significant time and financial investment. Here, we present a comprehensive, low-cost package to use a head-fixed environment for rodent behaviors. Our solution is complete with a flexible graphical user interface and can be built from mostly off-the-shelf components and operated by experimenters without any programming knowledge.

## Introduction

Head-fixed behavioral tasks are an invaluable tool for understanding how neuronal circuits drive behavior, and, thus, they have been a staple of systems neuroscience research for over half a century (Evarts, 1968; Wurtz, 1968; Moran and Desimone, 1985; Salzman et al., 1990; Shadlen and Newsome, 2001). This is because of three marked advantages of head-restrained preparations. First, they allow the precise and repeated use of microstimulation and recording modalities that give access to large neuronal populations (e.g., silicone probes, neuropixels, and two-photon and macroscopic imaging). Second, they allow accurate timing of behavioral variables like stimulus presentation, delays, response window, and reward and punishment delivery. Third, head fixation allows experimenters to manage some of the ambiguity resulting from the spatial aspects of a task (e.g., is the animal looking toward or away from the stimulus?). More recently, rodents, especially mice, gained traction in neuroscience research thanks to the access to cell types and circuits granted by modern genetic tools (Lein et al., 2007; Luo et al., 2008; O’Connor et al., 2009; Madisen et al., 2012; Huang and Zeng, 2013; Harris et al., 2014; Oh et al., 2014; Zingg et al., 2014). Thus, head-fixed preparations in rodents have been used for over a decade to study the neuronal circuits underlying behavioral output with great success across many laboratories (Poulet and Petersen, 2008; Cardin et al., 2009; Adesnik et al., 2012; Atallah et al., 2012; Harvey et al., 2012; Glickfeld et al., 2013; Lee et al., 2013; Olcese et al., 2013; Zagha et al., 2013; Fu et al., 2014; Zhang et al., 2014; Guo et al., 2014a; Li et al., 2015; Kim et al., 2016; Kwon et al., 2016; Burgess et al., 2017; Licata et al., 2017; Mohan et al., 2018; Pho et al., 2018; Stringer et al., 2019; Zhong et al., 2019; Aruljothi et al., 2020; Takahashi et al., 2020; Tang and Higley, 2020). While tasks used in these studies can vary greatly, they are typically built on one of the following three frameworks: two-alternative forced choice (2AFC), Go-NoGo (GNG), or passive reception of sensory stimuli. Each of these paradigms come with their own advantages and caveats, which has been described in detail by several authors (Carandini and Churchland, 2013; Bjerre and Palmer, 2020; Zagha et al., 2022). The importance of head-fixed rodent behaviors is well illustrated by the development of streamlined training protocols and efforts toward the standardization of such tasks (Guo et al., 2014b; Burgess et al., 2017; Goard, 2019; Bjerre and Palmer, 2020; Aguillon-Rodriguez et al., 2021). Recent years have also brought significant advancements in head-restrained rodent behaviors. These include improved hardware timing in complex environments (Solari et al., 2018) to better couple behavior to neuronal recordings, or the addition of trial self-initialization akin to the “fixation” step in primate experiments using a third lickspout (Marbach and Zador, 2017; Najafi et al., 2020) or levers (Musall et al., 2019). While some solutions to implement head-fixed rodent behaviors are available commercially, these typically carry price tags that may be prohibitive for junior laboratories or for those conducting neuroscience in more disadvantaged parts of the world. Commercially available solutions also tend to be more rigid, not allowing for much experimental flexibility. Thus, most laboratories opt for building their own behavioral apparatus. Such endeavors require considerable expertise in both hardware and software development and significant time investment. Furthermore, moving between different tasks typically involves building new setups, compounding the above difficulties. It is important to mention that the development of this impressive array of tasks may also introduce caveats. Each group using a unique experimental setup limits reproducibility and data interpretation across laboratories, a growing concern in all biomedical research, including neuroscience (Chesler et al., 2002; Botvinik-Nezer et al., 2020; Voelkl et al., 2020; Marek et al., 2022). Transparent and detailed documentation of experimental procedures, as done by many of our colleagues, is a critical step toward enhanced reproducibility (Marbach and Zador, 2017; Solari et al., 2018; Goard, 2019; Aguillon-Rodriguez et al., 2021).

One remaining caveat in many of the currently used tasks pertains to the difficulty in unambiguously separating sensory detection from higher-order cognitive processes like attention, working memory, and decision-making, as well as sensory–motor transformation and the final motor action resulting in the behavioral readout (Zagha et al., 2022). Currently, the best solution to this problem is temporally segregating the sensory detection phase of the task from the reporting phase. This is often achieved by adding a short delay between the end of stimulus presentation and the available response window. However, because of the inherent impulsiveness of rodents, even a short (a few hundred milliseconds) delay can drastically increase the time necessary for task acquisition. For example, while mice can typically learn sensory discrimination in three to four sessions, a 200 ms “lockout” period between stimulus and the response window necessitates an additional 10 session of training on average with a proportion of animals never reaching the desired performance (Aruljothi et al., 2020). The slow decay constant inherent in using intracellular calcium transients to report neuronal activity necessitates even longer delays in imaging experiments. Furthermore, to study cognitive processes like attention or short-term memory, delays on the order of several seconds may be desirable. A sensory discrimination task using 1–5 s delays could require ≥40 d of additional training (Gallero-Salas et al., 2021). Even when mice learn to withhold licking for the duration of these delay periods, the interpretation of such data is complicated by the experimenters’ inability to distinguish neuronal activity related to impulse control from attention, working memory, or decision-making. Additionally, it remains unclear whether in such tasks we measure the innate ability of animals to use working memory or whether the behavior also contains elements that were learned over the many sessions of training on longer delays (Liu et al., 2014; Kim et al., 2016). A solution to this problem is to mount the lick spout on a moving platform that allows the physical removal of the spout from the vicinity of the animal, making it available only during the response window (Goard et al., 2016; Kamigaki and Dan, 2017).

Here, we present a unified solution to the above detailed issues. We developed a behavioral platform for head-fixed rodents that allows the implementation of any of three behavioral frameworks (2AFC, GNG, and passive sensory stimulus presentation). Our head-fixed environment for rodent behaviors (HERBs) solution is an all-inclusive hardware and software package that can be built from off-the-shelf components with minimal (or no) need for custom-manufactured parts at comparatively low cost. The behavioral paradigms are controlled via a graphical user interface (GUI), making it easily accessible for those with minimal training or no programming experience. The GUI includes a plethora of selectable variables, yielding massive experimental flexibility in a single package. Since the entire system is open source, future additions to the design are also relatively straightforward, although making such changes will require some programming experience. HERBs includes servo-mounted lickspouts, allowing the experimenter to temporally segregate behavioral phases without the need for extensive impulse control training. Overall, our solution should provide a comprehensive, highly flexible, and affordable solution to those planning to use head-fixed rodent behaviors in their research.

## Materials and Methods

### Animals

All experiments were performed in accordance with the National Institutes of Health *Guide for the Care and Use of Laboratory Animals* and approved by the Institutional Animal Care and Use Committee (Approval AUP-20–076). Male and female C57BL/6J mice used in the study were either purchased from Charles River or bred in house and were group housed in a quiet, uncrowded facility on a 12 h light/dark cycle, with *ad libitum* access to lab chow and water (until the start of behavioral training).

### Surgeries

To express a genetically encoded calcium indicator, GCaMP6s, mice were anesthetized with 1.5% isoflurane (v/v) mixed with pure oxygen, and analgesia was provided via 5 mg/kg meloxicam delivered subcutaneously. A small craniotomy was made over the posterior parietal cortex [PPC; distance from bregma: anteroposterior (AP), −2.1 mm; mediolateral (ML), 1.7 mm; dorsoventral (DV), 0.45 mm]. Each mouse received one 300 nl injection of adeno-associated virus (AAV2.9-hSynapsin1-GCaMP6s, Addgene). Injections were made via beveled glass micropipette (model EG-402 Microgrinder, Narishige) at a rate of ∼25 nl/min using a microinjection pump (model UMP3T, WPI). After injection, pipettes were left in the brain for ∼5 min to prevent backflow. Two weeks following virus injection, animals were implanted with a titanium headpost and an ∼3-mm-diameter cranial window was opened above the injection site. An imaging window, consisting of a 3 mm circular coverslip attached to a 5 mm circular coverglass using an ultraviolet-curing adhesive (Norland Products), was inserted into the craniotomy and secured to the skull with dental cement (Metabond). Mice were allowed to recover for a minimum of 2 weeks before imaging.

### Muscimol injection

Head-posted mice were bilaterally implanted with sealable cannulas (PlasticsOne) above the PPC (distance from the bregma: AP, −2.1 mm; ML, −1.7 mm) before covering the remaining skull surface with dental acrylic (Stoelting). On the day of inactivation experiments, cannulas were opened and 100 nl of muscimol solution (2 mm) or ACSF vehicle was injected using a Hamilton syringe connected to the infusion insert via mineral oil-filled Teflon tubing. Infusion was conducted 30 min before the start of the experiment. Cannula locations were histologically confirmed *post hoc*; 100 nl fluorescein (1%) solution was injected through the cannulas followed by PFA fixation. Sections (50 μm) were produced on a vibrating microtome (Compresstome Vibrating Microtome) and were mounted on microscope slides with Prolong antifade containing DAPI.

### Water restriction

To motivate task engagement, mice were water deprived as described previously (Guo et al., 2014b). Briefly, 1 week before the start of behavior, mice were shifted to 1 ml of water/d, administered precisely to each animal. After ∼1 week, mice on this water schedule reached and stabilized at 85% of their starting weight. Behavioral training started at this point. Typically, mice gathered 600–800 μl water during a day of training. When mice did not receive 800 μl, they were supplemented to that value.

### Behavioral training

All behaviors were trained in stages as described before (Guo et al., 2014b; Goard, 2019). Briefly, mice were first habituated to the rig by gradually increasing rig time from ∼10 s to 30 min over the course of 3–4 d. Each day, mice went through multiple sessions of habituation. Keeping a running disk in the home cage may help the mice habituate to the running disk quicker. Once the animals habituated to head fixing and were running on the wheel comfortably, they learned to lick the center lick spout for water. At this stage, the spout was extended and water was dispensed by the user via the GUI. If the paradigm required side spouts (2AFC), mice were introduced to side rewards in a similar fashion. Next, mice went through classical conditioning using the Free Reward feature. Spouts extended automatically, and the correct spout immediately dispensed a reward that the animal could collect at will. This was coupled with the appropriate stimulus. In the next stage, mice went through operant conditioning and only received water reward following a lick on the appropriate spout. To reinforce deliberate choice (especially in 2AFC), lick requirements can be gradually increased to four or five licks per second over several days. Licking the incorrect side resulted in punishment (noise and/or a small air puff), and a brief timeout. When training for the 2AFC, the Retrial Mode allowed the mice to try again on the same stimulus, withholding the next trial until they licked the correct side. When mice showed stable performance (75% correct choices in 2AFC or >1.5 d′ in GNG for 3 consecutive days), they could progress onto the next stage. For psychometric testing in 2AFC, 70% of the trials were the same as the training trials and 30% were test trials with novel stimuli. The inclusion of training trials appears necessary to maintain motivation in the task. To minimize learning effects during testing, retrials and punishments were given only on trials displaying the training stimuli. In GNG, training (no delay) and the various delay trials were distributed equally.

### Analysis of behavioral data

HERBs saves a text (.txt) file with all parameters and response times stamped. All parameters set in the GUIs are also saved in a separate .xls file for user convenience. The produced text file can be used to assess performance without having to record all behavioral parameters through a data acquisition (DAQ) board. The text files are updated in semi-real time, with an ∼1 min lag. Thus, if desired, daily performance can be plotted with little lag as the animal is training. The same data can be reproduced from recoding the outputs of the board throughout a data acquisition board. It is to be noted that the precision of time stamps in the text file are subject to operating system, MATLAB and other internal clocks in the used PC. Consequently, while the relative timing of events in the text file may be accurate to ∼2–3 ms, it is not advised to use this output for synchronization with neuronal recordings. The output to a data acquisition board is precise to a few microseconds (primarily subject to data acquisition rates and the internal timers of the Arduino), which is much more suitable for alignment with neuronal recordings.

Learning curves were obtained by plotting the percentage of correct trials against the session number across multiple days. Psychometric curves for 2AFC were obtained by plotting the percentage of right-side licks for each light stimulus column from left to right (0–8) in a given session. Panels 0 and 8 are the training panels, and panels 1–7 are the six novel stimuli. For psychometric analyses, choice data in the 2AFC task were fitted with a four-parameter sigmoid function (Wichmann and Hill, 2001), as follows:

f (x, α, β, g, l) = g + (1 – g – l) [1 + exp (x – α/β)],

where *x* is the location of the light panel from left to right, α is the mean value of the distribution representing the choices of the animal, β is the discrimination sensitivity of the animal, and *g* and *l* are the guess and lapse rates, respectively.

To calculate discriminability (*d*′) in the GNG paradigm, we used the following standard *d*′ calculation:

d′ = z(FA) – z(H),

where d′ is the discriminability index, *z*(FA) is the *z*-scored false alarm rate, and *z*(*H*) is the *z*-scored Hit rate. We completed these analyses using custom Python scripts.

### Hardware

The goal of our hardware development was to build a single apparatus that can run programs for different types of rodent behaviors (2AFC, GNG, or passive sensory stimulus perception). We drive the hardware using a highly customizable GUI allowing the user to set experimental parameters without any programming knowledge. To make the hardware open source and easily reproducible, we avoid custom parts as much as possible and instead use affordable, of-the-shelf components. We also provide a step-by-step guide to building both the mechanical and electrical components of the system [Extended Data 1: HERBs mechanical hardware build instructions and HERBs electrical hardware build instructions (or on GitHub: mechanical, electrical)].

10.1523/ENEURO.0018-23.2023.ed1 Extended Data 1 Extended Data containing 3D files, all necessary code, example video clips, parts list, detailed build instructions and full software documentation. Download Extended Data 1, ZIP file.

**Figure 1.**
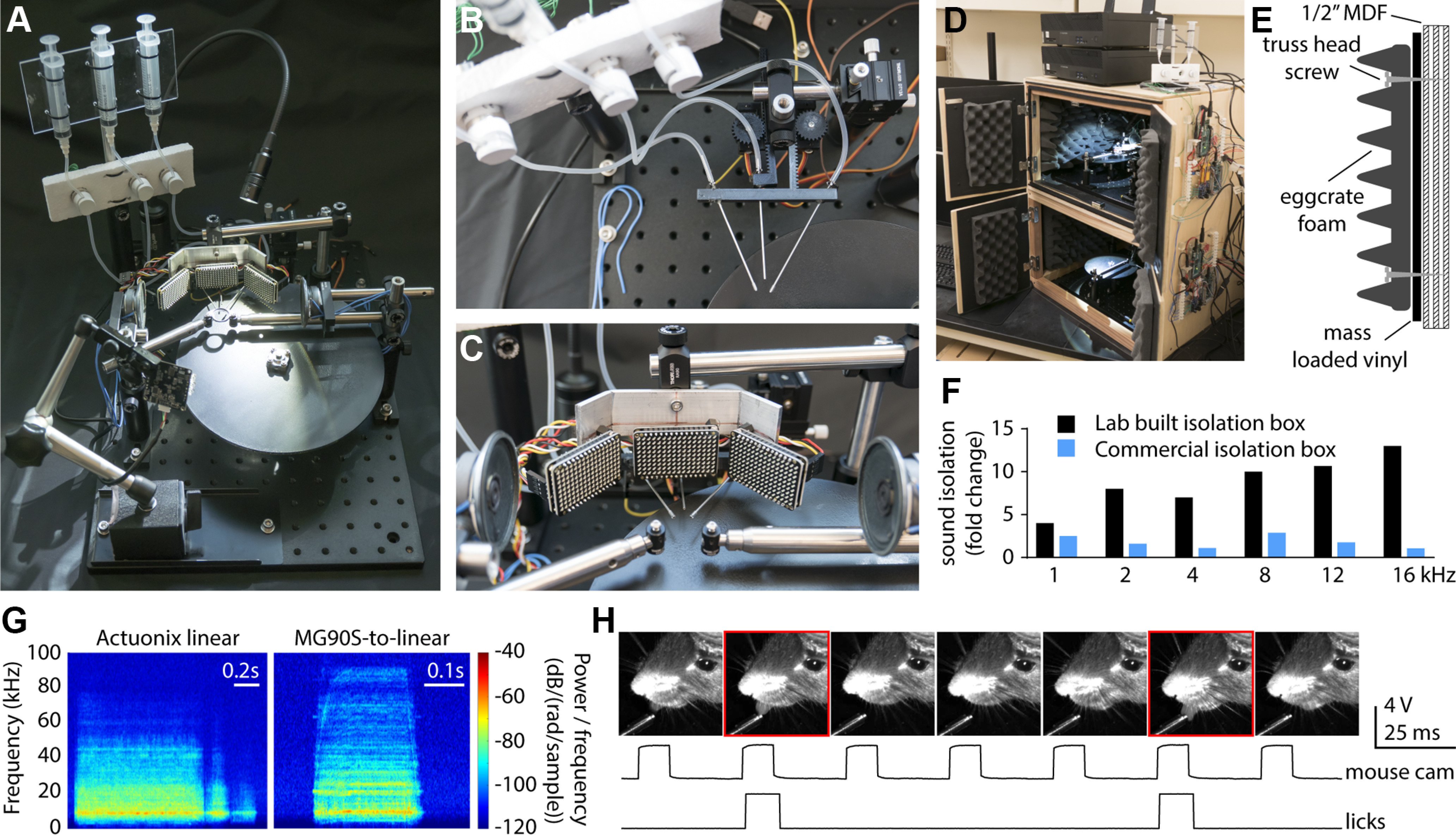
HERBs hardware. ***A***, Full hardware assembly. ***B***, Top-down view of the reward delivery system, complete with three solenoid valves and motorized lick spouts using the rotary servo to linear movement conversion. ***C***, Placement of the three 16 × 9 LED panels and two speakers around the head-holder bars. ***D***, Photograph of the isolation box (double rig) with the behavioral apparatus inside and electronics mounted on the right side of the box. ***E***, Schematic cross section of the isolation box wall. ***F***, Sound attenuation performance of the in house-built isolation box compared with an affordable commercially available solution at different tones. ***G***, Sound profile (spectrogram) of the linear actuator (Actuonix; left) and the rotary servo-to-linear converter (right). ***H***, Video confirmation of the lick detector system. Red boxes mark the frames where a lick was observed (top), compared with the registered camera frames (middle) and the detected licks (bottom).

The mechanical elements of the apparatus are constructed with parts available from Thorlabs, McMaster-Carr, and Amazon (or any other vendor for generic parts; Fig. 1*A*). Electrical components include an Arduino MEGA 2560 board that serves as the central input/output interface; an Arduino DUE for auditory tone generation; integrated circuit boards for capacitive lick detection, power control, solenoid pinch valves, and audio amplifier; and LED panels for stimulus presentation. The full parts list can be found in the Extended Data 1 [HERBs_parts_list and on GitHub (parts list)]. The only components that may need custom machining or 3D printing are the following: (1) a holder for the LED panels [this can be 3D printed using the model in the Extended Data 1 3D files and on GitHub (LED holder) or simply made from a 150 × 30 × 2 mm sheet of aluminum bent to a crescent shape and drilled], and for auditory experiments this part is not needed; (2) a spout holder when using linear actuators [3D printed using the model in the Extended Data 1: 3D files and on GitHub (spout holder - linear actuator) or fabricated using an 80 × 25 × 10 mm piece of plastic or polyurethane foam]. If this part is used with linear actuators, part 3 in this list is not needed; and (3) linear actuator converter if using rotary servos [3D printed using the model in the Extended Data 1: 3D files and on GitHub (servo-to-linear converter)]. This can be substituted with linear actuators if 3D printing is not an option. If the rotary servos are used, part 2 in this list is not needed. The converter is a modification of the original design (https://www.thingiverse.com/thing:4557945).

Syringe and solenoid valve holders can be constructed from a piece of plastic and polyurethane foam with minimal cost and effort (Fig. 1*A*, Extended Data 1 (HERBs mechanical hardware build instructions), but any solution (e.g., zip ties to a column) will work.

Central to our design is a moving spout setup (Fig. 1*B*). This mechanism enables control over the availability of reward spouts during the experiment via servos or linear actuators. Spout movement is essential for controlling the temporal delays in attention or working memory type tasks. For example, during a working memory delay, the stimulus and response windows are separated by the physical removal of the reward spout, circumventing issues with impulse control. This approach may also drastically reduce training time for such tasks as the animals do not need to learn to withhold licking during the delay period.

We provide two different solutions for implementing spout movement. These have distinct advantages and disadvantages with regard to movement speed, emitted noise profile, availability, and serviceability. For an off-the-shelf solution, HERBs can use L12-I-type “rod” or linear actuators (Actuonix) that contain an internal position controller. These actuators take an analog voltage command to set position. Actuonix actuators can have a range of 30–100 mm depending on the version purchased, require a separate 12 V power supply, move at a speed of 25 mm/s (different gearing options are available but have not been tested), and produce an audible noise when measured directly next to the device (∼10 dB above ambient noise measured within 20 mm). At the position of the animal (∼150 mm from the actuator) the noise is ∼1–2 dB above ambient noise with a sound profile showcased in Figure 1*G*. The lifetime of these actuators is ∼3–4 months with ∼500 movements/d, 5 d/week. They cost approximately $80 each. If 3D printing is an option, instead of the Actuonix actuators, we recommend using MG90S servos attached to the servo-to-linear converter assembly documented in our Extended Data 1. These have a similar range (∼40 mm), move at a considerably faster speed (50 mm/s), use a 5 V power supply, and are controlled via pulse-width modulation through the Arduino MEGA. The sound produced by the servo and linear translator assembly can vary across builds, in our hands ranging from 6 to 15 dB above ambient noise when measured within 20 mm of the device. This falls to <1–6 dB above ambient at the location of the animal (distance, 150 mm) with a sound profile shown in Figure 1*G*. Sound levels were measured using a Class 1 Sound Level Meter (catalog #DSM403SD, General Tools), the spectrum of the noise was recorded using a Pettersson M500-384kHz USB UL Ultrasound Microphone via the BatRecorder app. Servo lifetime is highly variable, but they can last up to 6 months with daily use; they cost approximately $3, and we found them to be easier to replace than the linear actuators. Additionally, the design can be easily modified by users experienced with 3D design software. To choose which type of servo motion is used, the user simply selects the actuator type (linear or servo) in the GUI. The distance traveled by the spouts is determined by the value entered into the appropriate box in millimeters. Spout movement can also be completely disabled in the GUI if desired by the experimenter.

Licks are detected via capacitive touch breakout boards (catalog #AT42QT1010, SparkFun), connected to the metal lick spouts via separate wires and alligator clips (see build instructions in the Extended Data 1). The interrupt routines controlling the touch sensors in the Arduino MEGA scripts are disabled for 10 ms after a lick has been registered, allowing a maximum detectable lick rate of 100 Hz. This provides a markedly higher detection rate than the typical licking behavior, which is ∼10 Hz. To test the accuracy of the lick detector, we recorded video footage of the licks with an infrared camera (model Alvium 1800 U-501 M, Allied Vision) at 30 frames/s. Frames corresponding to each lick were identified manually and compared with the output of the capacitive touch sensor. We found a >97% match between the licks detected in the video and by our electronics (tested on a 5 min video, ∼150 licks total) with lick frames closely aligned to the electronically detected responses (Fig. 1*H*). Reward amounts must be calibrated for each individual spout by measuring the volume of the water droplet using a pipettor and adjusting the solenoid valve open time in the GUI until the desired amount is dispensed. Although we did not observe drift in the reward amount, we recommend frequent (weekly) calibration. In two-photon imaging applications, we did not detect any artifacts coming from spout movement, lick detection, or solenoid valve activation. However, the capacitive detectors will likely produce artifacts in electrophysiology recordings (not tested in our laboratory). If the experiment requires electrophysiology, we recommend using infrared lick detectors instead [e.g., the Optical Lickometer, Sanworks (https://sanworks.io/shop/viewproduct?productID=1020)].

Stimuli are delivered via bilateral audio speakers or a crescent of LED panels (16 × 9 pixels/panel; Fig. 1*C*). This allows lateralized stimulus selection to accommodate flexible choices for recording hemisphere and a plethora of stimulus combinations. The LED panels allow for a great range of stimuli that are easy to set up in the Arduino MEGA (example code provided in the Extended Data 1: Software Documentation, section 5). Our software includes a library of tones ranging from 2 to 32 kHz, white noise, and a set of visual patterns (full panel, multiple stationary bars at four different angles, or a single moving bar of 1, 2, or 3 pixel width). Our package currently does not include internal routines for sound level calibration. Since the response of the speakers depends on the tone frequency, it is recommended that the user calibrates left and right speakers independently and for each tone pitch to ensure equal sound pressure levels of the stimuli. This can be done using a sound level meter or an ultrasound microphone (e.g., Pettersson M500-384).

We recommend enclosing the apparatus in a sound-proofed environment (Fig. 1*D*). While such enclosures can be purchased as off-the-shelf parts, testing in our laboratory and by others (Solari et al., 2018) indicates that simple solutions made in house [e.g., 0.5 inch plywood or medium-density fiberboard (MDF) and 1 pound/square foot mass loaded vinyl or other sound insulators; Fig. 1*E*] can be very effective at acoustic insulation (Fig. 1*F*). Sound attenuation for the enclosure was measured using a Pettersson M500-384kHz USB UL Ultrasound Microphone via an app (BatRecorder version 1.0R172) on a Lenovo tablet. Tones were generated via the Tone Generator app on a cellphone that was placed 2 feet from the enclosure. We subtracted the ambient sound pressure from the sound pressure measured at the generated tone and expressed sound attenuation as a ratio (fold change) of the calculated sound intensity with the door of the enclosure open divided by sound intensity with the door closed.

**Figure 2.**
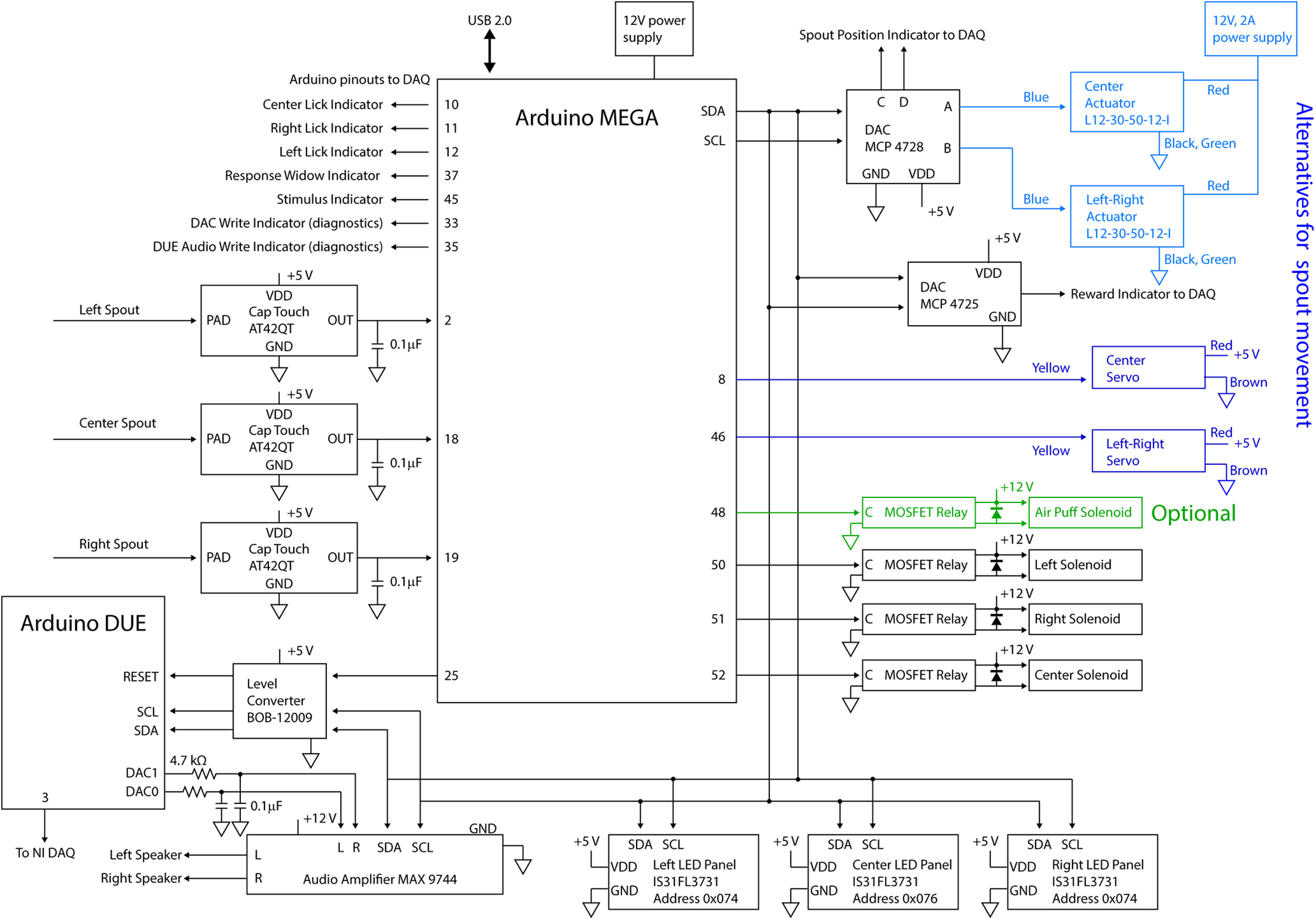
HERBs wiring diagram. The diagram shows all wire contacts with the pinouts noted on each board. The two alternatives for wiring up servo movements are indicated in blue. The light blue wiring diagram should be followed when using linear actuators, while the dark blue route indicates wiring for rotary servos. Follow the green wiring for the optional air puff punishment delivery (not necessary for operation). All wiring indicated in black is necessary for operation enabling both audio and visual stimulus/punishment presentation.

The wiring diagram for the setup is provided in Figure 2, with black indicating all necessary components, green showing optional components, and blue indicating alternative wiring options for linear actuators versus servos for spout movement. For a detailed guide on how to build the electrical components of the system, refer to Extended Data 1 (HERBs electrical hardware build instructions).

### Software

To drive the above detailed hardware, the central Arduino MEGA communicates with a PC running MATLAB via a serial port (USB 2.0 interface). The software was developed in MATLAB version 2022a with the only dependence being the Instrument Control toolbox (see Extended Data 1: Software Documentation, section 1). GUIs were created using the MATLAB App designer. Since the .mlapp files are compiled code, to make our package truly open source, we also include easily editable .m files for each GUI (stored in the “Source_code_for_APPs” folder of our package). We have tested our design on Windows 10 and Windows 11 PCs, testing hardware included Intel (core i3, i5, and i7) and AMD (Ryzen 5 and 7) processors with a minimum of 8 GB of RAM and 128 GB of HDD (hard disk drive).

The state machines for 2AFC and GNG, as well as the control functions for sensory stimulus display, are programmed in MATLAB. Detailed state machine diagrams are provided in Extended Data 1 [Software Documentation, sections 8 (2AFC) and 9 (GoNoGo)]. MATLAB communicates with the Arduino MEGA by sending data through the serial interface. For example, the valves are controlled by sending a 3 byte instruction to Arduino MEGA as follows:

write(arduino,[‘k’ 1 1] “uint8”),

where the first byte indicates the device type to be controlled (e.g., “k” refers to the valves), the second byte indicates the device assignment (e.g., “1” refers to Center), and the third byte indicates the action (e.g., “1” instructs to open the valve). The Arduino first receives the first byte, decodes it, and then reads two more bytes to determine which device is operated and what the action is (see Extended Data 1: Software Documentation, section 4 for example code). All settings in the GUIs are saved in the header of the .txt file that records MATLAB command line printouts, in a separate .xls file, and in an .m file. After exiting the GUIs, the .m settings file is overwritten with the parameters at the time of shutting the program down. When restarted, these latest settings will be loaded to the GUI. Settings may also be saved and loaded via the appropriate buttons in each GUI. For safety, the main folder also contains separate “default_settings” .m files for each GUI, which allows rollback to the original settings. If there are no .m settings files present, the GUI will automatically load with these default_settings.

The Arduino MEGA handles all the signal timers related to the LED panel on/off/number of cycles and audio on/off/number of cycles. Reward durations are also handled by Arduino timers, affording greater precision. All other timers guiding the state machine are handled by MATLAB (see the list of necessary Arduino MEGA libraries in Extended Data 1: Software Documentation section 2).

For visual stimulus presentation, we use 9 × 16 LED matrices. These are cost effective, highly flexible, and easy to program devices for generating visual stimuli (Swanson et al., 2021). LED panels are directly programmed in the Arduino MEGA according to the manufacturer instructions (https://learn.adafruit.com/i31fl3731-16x9-charliplexed-pwm-led-driver). They do not need extra software like Psychtoolbox that would otherwise be necessary to drive more advanced displays. Extended Data 1 (Software Documentation, section 5) provides examples for how to program/modify the operation of these panels. HERBs includes preprogrammed stationary stimuli and a single moving bar, but these panels can produce more complex stimuli, even including sinusoid-like drifting gratings (https://www.adafruit.com/product/2974).

Auditory signals are generated using a 32-point lookup table in the Arduino Due and then sent to the Due built-in digital analog converters (DACs) via direct memory access. This process allows the generation of sine waves with great precision to produce near-pure tones (see the list of necessary Arduino Due libraries in Extended Data 1: Software Documentation, section 3; a description of the process and the code used can be found in the Extended Data 1: Software Documentation, sections 6 and 7).

**Figure 3.**
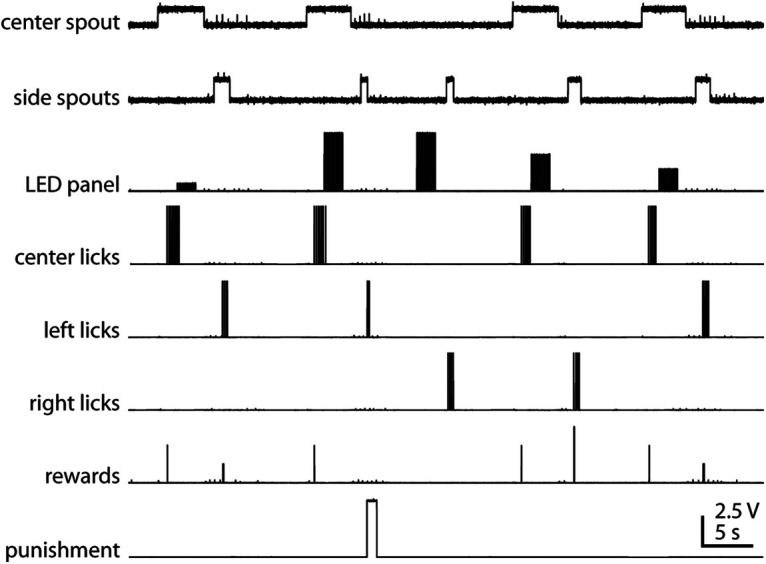
Signal output from HERBs, recorded on a data acquisition device during 2AFC behavior. The “center spout” signal shows the movement of the central lick spout, with high voltage indicating the spout being in position for the animal to reach it. The “side spout” signal shows the simultaneous movement of the left and right lick spouts, with high voltage indicating the spouts being in position for the animal to reach it. The “LED panel” shows the signal indicating when the LEDs are on, with the voltage encoding the location of the stimulus along the LED crescent. “Center licks” registers licks on the center spout. “Left licks” registers licks on the left spout. “Right licks” registers licks on the right spout. “Rewards” registers when the reward is delivered, with the voltage encoding the spout (high, right reward; medium, center reward; low, left reward). “Punishment” indicates the on and offset of the punishment tone following an incorrect choice.

Output from the Arduinos is sent to a DAQ board (e.g., National Instruments, Measurement Computing, or Cambridge Electronic Design) via DAC boards (available from Adafruit or SparkFun). We have tested NI 6xxx series (e.g., USB-6218) and MC USB-1208FS PLUS boards. These are available with USB connection (no need for BNC breakout boards) and have the bandwidth to record output channels at a 5 kHz sampling rate. NI boards work well with WaveSurfer [Adam L. Taylor, Janelia Research Campus (https://wavesurfer.janelia.org)], while MC boards work with the MCC DAQ Software (https://www.mccdaq.com/Software-Downloads.aspx). Both are free software packages to digitize and record analog data. This output allows the alignment of physiology recordings (e.g., two-photon calcium imaging) with 0.2 ms precision (primarily limited by the bandwidth of the DAQ board used). The output contains the following: spout movement, sensory signal, licks on each spout, rewards on each spout, punishment, and microscope frame rate (for two-photon imaging), with connections to spare for future additions like a rotary encoder, pupil camera, and frame rate. An example of the output recorded from a 2AFC session is shown in Figure 3.

### Two-photon imaging

Calcium imaging data were acquired on a MOM two-photon microscope (Sutter Instrument) equipped with an 8 kHz resonant scanner and a 20× (0.9 numerical aperture) Olympus objective, and coupled to a Ti-Sapphire femtosecond pulsed laser (model Chamelon Ultra II, Coherent) via a Pockels cell (Conoptics) for power modulation. Excitation light was set to 940 nm, fluorescence was collected through filter sets appropriate for GCaMP via a GaAsP photomultiplier detector. Images were collected at 30 Hz frame rate with 256 × 256 pixel resolution using ScanImage 5.4 software (Vidrio Technologies) from Layer2/3 of the PPC (depth from the brain surface, 150–250 μm). Multisession images were aligned using the vasculature of the brain surface to find the approximate region, and then cells were overlayed via the motion correction utility in Scanimage 5.4.

### Two-photon data analysis

Calcium imaging data were registered and segmented using Suite2P (Pachitariu et al., 2017). After neuropil subtraction, neuronal responses were aligned to stimulus onset, averaged, and displayed via custom scripts in Python 3.7 (Anaconda distribution).

### Data availability

All necessary MATLAB and Arduino codes are available in the Extended Data 1 (HERBs – code and in our GitHub repository). All documentation and installation instructions are available in the Extended Data 1 and on a wiki page and in Extended Data 1: Software Documentation, section 12. Issues can be reported on our GitHub Issues page.

## Results

### Two-alternative forced choice and categorical decision-making

**Figure 4.**
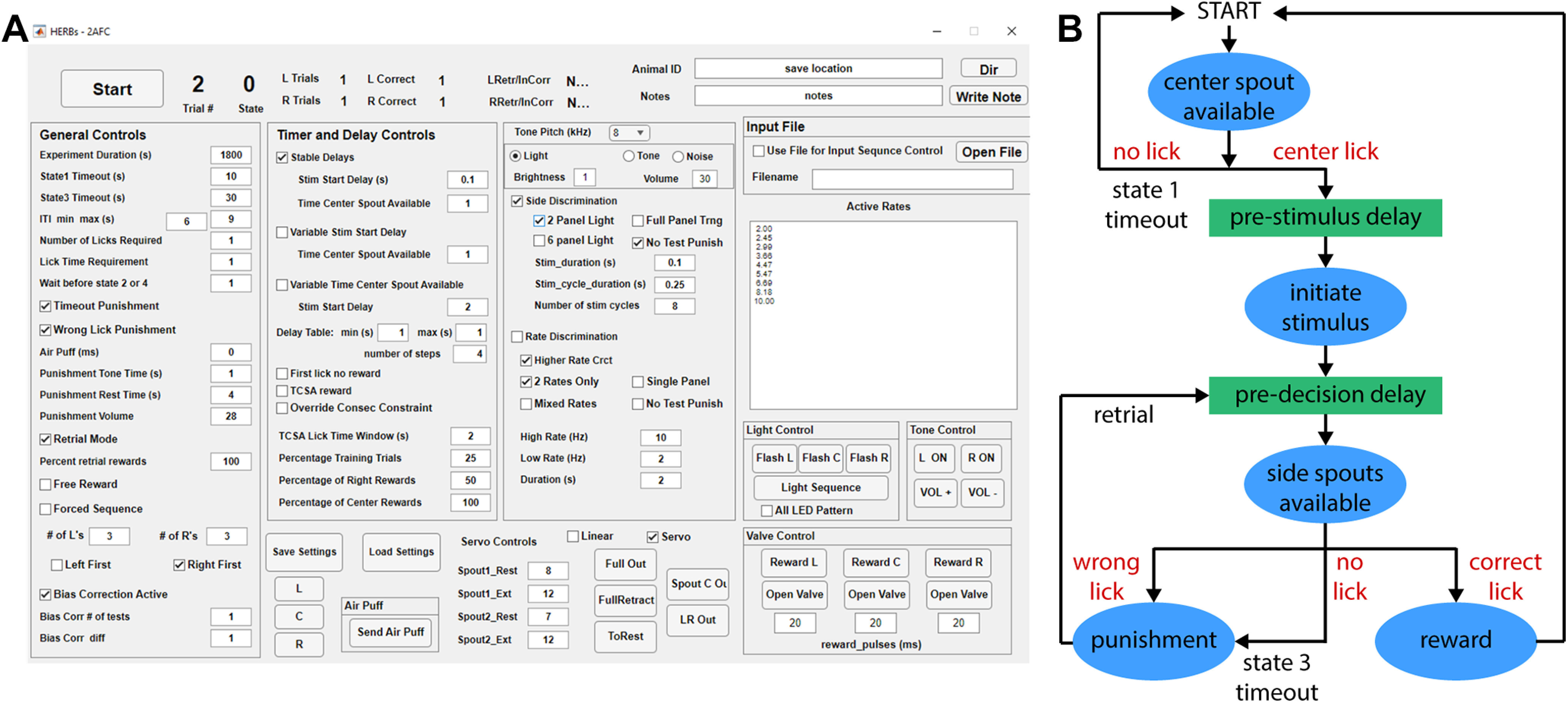
HERBs 2AFC GUI and state machine. ***A***, GUI for the 2AFC paradigm. A full description of all controls can be found in the Extended Data 1: Software Documentation, section 8. ***B***, Simplified state machine for the 2AFC task. The full, detailed state machine can be found in Extended Data 1: Software Documentation, section 8.

Our goal was to create a GUI that provides great flexibility to the user to set up experiments based on the 2AFC framework (Fig. 4*A*). To start the GUI, add (with subfolders) the “Behavior_GUIs” folder to your MATLAB path (or cd to the “Behavior_GUIs” folder in MATLAB, launching the GUI will automatically add the relevant folders to the path), type:

≫HERBs_2AFC

into the MATLAB command line, and hit enter.

**Figure 5.**
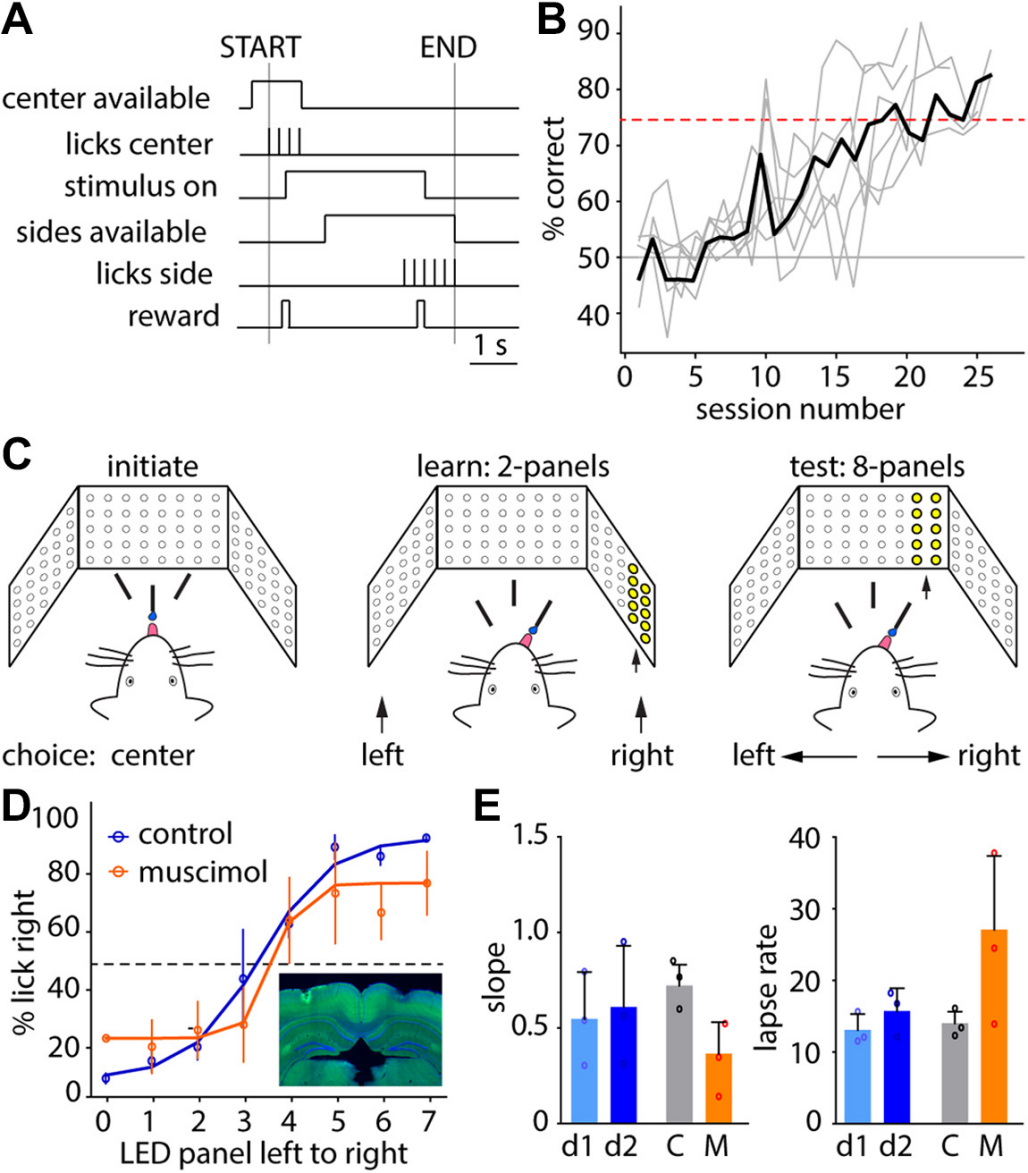
Visuospatial categorical decision-making using the 2AFC paradigm. ***A***, Schematic showing the trial structure. ***B***, Learning curves showcasing *n* = 6 mice learning the left–right discrimination. Light gray lines are data from individual animals; the solid black line is the mean. ***C***, Schematic showing the categorical decision-making paradigm. Trials are initiated on the center spout (left), followed by left or right stimulus (middle) during training, or an intermediate stimulus location (right) during the testing phase. ***D***, Psychometric performance in visuospatial categorization following bilateral vehicle (blue) or muscimol (orange) injection to the posterior parietal cortex. Inset, Fluorescence image showing the canulation site; blue, DAPI; green, fluorescein. ***E***, Comparison of the slope (β) and lapse rate (l) fitting coefficients on consecutive days (d1 and d2 in blue, *n* = 3) or between vehicle-injected (gray, *n* = 3) and muscimol-injected (orange, *n* = 3) animals. C, Control; M, muscimol.

Detailed description of each parameter and function in the GUI is provided in the Extended Data 1 (Software Documentation, section 8). Two key elements of our design are (1) the self-initiated nature of the task via a central lick spout (Marbach and Zador, 2017; Najafi et al., 2020) and (2) the experimenter’s ability to control time delays between task stages via spout movements (Goard et al., 2016; Kamigaki and Dan, 2017; Fig. 4*B*). A behavioral trial starts out with the central port made available for initiation, followed by a definable prestimulus delay. Rewards for trial initiation are controlled by the “Percentage of Center Rewards” box where the user can set the probability of rewarding licks on the Center Spout. Stimulus presentation is then followed by a second selectable delay period before the side ports are made available for reporting a decision. The cycle is then concluded with an intertrial interval (ITI) until the next trial can be initiated (Fig. 5*A*). The length of the ITI is defined in a range (minimum and maximum) and varies from trial to trial following an exponential distribution. This task structure may be used to study simple two-choice decision-making where the choice may be spatial (left vs right) or stimulus rate (frequency) discrimination based in either visual or auditory modality. Mice can learn to perform such a task with stable performance of >75% correct in 20–25 sessions (Fig. 5*B*) when trained 5 d/week (a video clip showing a mouse in training can be seen in Extended Data 1: HERBs - 2AFC example.mp4).

Following adequate training on any of the above rules, a novel set of stimuli may be introduced. Currently, this can be visuospatial by introducing novel locations on the LED crescent (Fig. 5*C*) or by introducing novel stimulus rates in either auditory or visual modality. This allows the researchers to test psychometric performance in categorical decision-making. A possible future addition would be categorization of novel tone pitches; however, our current solution does not offer this possibility. In our example, we used a visuospatial categorization paradigm where performance decreased with spatial shift of the stimuli until the mice performed at chance level when the stimuli columns were near the center of the field of vision (Fig. 5*D*). This task lends itself well to determining the effect of manipulations. Here, we show the effect of the bilateral inactivation of the PPC on the categorization of novel locations (Fig. 5*D*,*E*). This effect is similar to what was seen in audiospatial categorization experiments (Funamizu et al., 2016). Stimulus locations or rates are randomly drawn without replacement from the pool of available possibilities until the pool is depleted, then the pool is reshuffled and drawn again, ensuring equal sampling of all categories.

To prevent the animals from trying to use hidden underlying temporal structures in the task, trial selection is randomized but follows two rules: the same trial type (left or right) cannot be presented more than three times in a row, and there cannot be more than four back-and-forth jumps in a row between opposing trials. If the randomization produced a conflict, the next trial is forced to obey the above rules. These rules can be turned off by checking the “Override Consec Constraint” box. This may be necessary if the experimenter desires >66% of the trials to go to one direction.

To aid the separation of behavioral stages (e.g., initiation, stimulus encoding, delay activity, or decision and sensory–motor transformation), the above-described simple two-way decision-making or novel stimuli categorization can be conducted while setting prestimulus and poststimulus delays that remain unchanged during the entire session. In addition, the GUI offers straightforward functionality to test performance across varying prestimulus or poststimulus delays by controlling the relative movement of the center and side lickspouts. Checking the “Variable Stim Start Delay” box allows the use of an arbitrary number of steps for the delay between the trial initiation and the stimulus onset while the poststimulus delay (set in the “Time Center Spout Available” box) remains unchanged. Continued licking of the center spout (that may be interpreted as continued engagement, akin to fixation in tasks designed for primates) can be rewarded via the “TCSA reward” checkbox. In contrast, checking the “Variable Time Center Spout Available” box will allow the setting of an arbitrary number of delay steps between stimulus presentation and the opening of the response window (when the side ports become available) while maintaining a stable “Stim Start Delay.” Delays are randomly drawn from the distribution defined in the “Delay Table” without replacement until all possibilities are exhausted, and then the distribution is rerandomized to give approximately equal sampling of all possible delays. The number of steps is only limited by the number of trials the animal performs in a given session. Typically, we limit steps to five or six per side to ensure a sufficient number of trials on each.

### Go-NoGo paradigm

**Figure 6.**
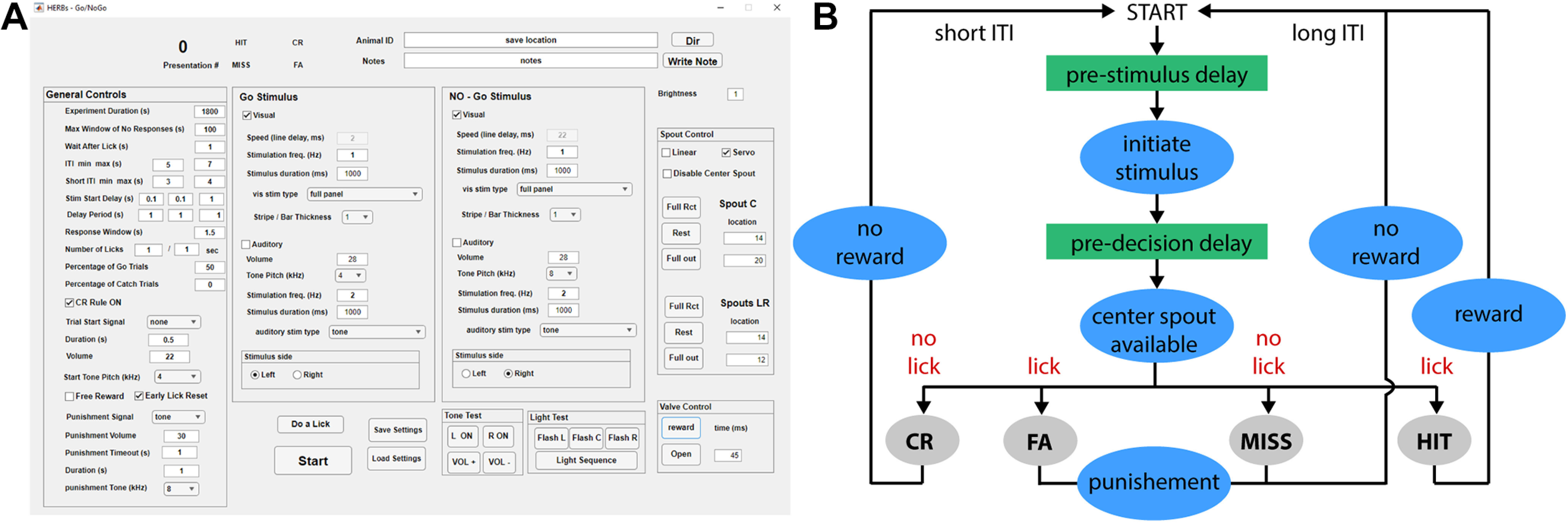
GNG GUI and state machine. ***A***, GUI for the GNG paradigm. Detailed description of all controls can be found in the Extended Data 1: Software Documentation, section 9. ***B***, Simplified state machine for the GNG task. The full, detailed state machine can be found at the end of Extended Data 1: Software Documentation, section 9.

Our goal was to produce a comprehensive GUI that allows control of all necessary settings for a behavioral task based on the GNG framework (Fig. 6*A*). To start the GUI, add (with subfolders) the “Behavior_GUIs” folder to your MATLAB path (or cd to the “Behavior_GUIs” folder in MATLAB; launching the GUI will automatically add the relevant folders to the path) and type:

≫HERBs_GoNoGo

into the MATLAB command line and hit enter.

**Figure 7.**
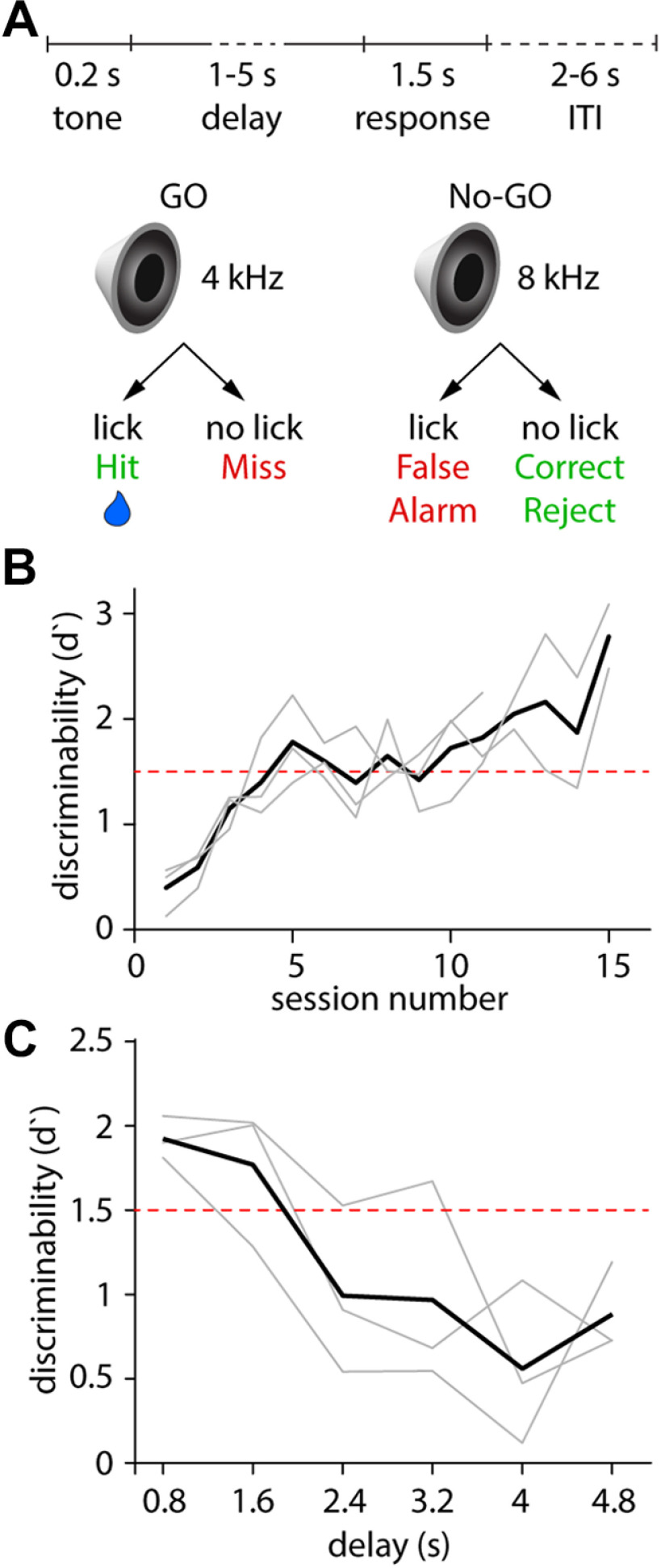
Performance across multiple delays in an auditory GNG paradigm. ***A***, Schematic of the GNG task. ***B***, Learning curve of *n* = 3 mice. ***C***, Auditory discrimination performance (expressed as *d*′) across varied delays. Light gray lines are data from individual animals; the solid black line is the mean.

The basic structure follows the task used by a number of laboratories (Zagha et al., 2013; Guo et al., 2014a; Goard et al., 2016; Batista-Brito et al., 2017; Aruljothi et al., 2020). Detailed description of each parameter and function in the GUI is provided in the Extended Data 1 (Software Documentation, section 9). Similar to the above-described 2AFC task, the key element of our design is a servo-mounted, moving lickspout that allows precise timing of prestimulus and poststimulus delays (Figs. 1*B*, 6*B*). Trials start automatically following an ITI and can be signaled by a visual or auditory cue (“Trial Start Signal”). Following a prestimulus delay period [set in “Stim Start Delay (s)”], a variety of auditory or visual stimuli may be presented. The user has independent control over the style (e.g., stationary bars, a moving bar, and flashes or tones of different pitch and presentation frequency) and location (left or right) of the stimulus for Go and NoGo. Stimulus presentation is followed by a user-controlled poststimulus delay [“Delay Period (s)”] before the lickspout is extended at the start of the response window. Prestimulus and poststimulus delays are set in three boxes (from left to right): minimum, maximum, and number of steps. The user can define a single delay by entering 1 as the minimum, 1 as the maximum, and 1 as the number of steps or an arbitrary number of delays between a minimum and maximum values (e.g., 1 as the minimum, 6 as the maximum, and 6 steps would produce delays of 1, 2, 3, 4, 5, and 6 s). Delays are separately drawn for Go and NoGo trials from the defined pool, randomly without replacement. When the pool is depleted, values are reshuffled and redrawn, ensuring equal sampling. A correct rejection of the NoGo stimulus leads to a shortened ITI (“Short ITI”) and can be followed by a Go trial (if the “CR rule ON” box is checked) or a random trial (“CR rule ON” box unchecked) with a distribution defined in the “Percentage of Go Trials” box. All other trials are followed by a “long-ITI.” All ITIs are defined in a range (minimum and maximum) and implemented with an exponential distribution. Only a correct choice (HIT) is rewarded by opening the solenoid valve and dispensing water (Fig. 7*A*).

Following habituation, mice can learn sensory discrimination (*d*′ > 1.5) in four to five sessions (Fig. 7*B*; a video clip of a mouse performing side discrimination can be seen in Extended Data 1: HERBs - GoBoGo example.mp4). To test the effect of various delays on sensory discrimination performance, the experimenter can introduce a set of delays. To showcase the functionality of the GUI, we set six delays to range from 0.8 to 4.8 s after the termination of a 200 ms stimulus [“Delay Period (s)” set to 1: 5: 6; “Stimulus Duration (ms)” set to 200]. We also reduced the volume of the auditory stimulus to ∼3 dB above ambient noise (training occurred at ∼15 dB above ambient noise). Mice considered expert in the discrimination task without delay showed diminishing performance with longer delays (Fig. 7*C*). Our moving spout hardware allowed us to test the innate performance of the animals across delays without any prior exposure to this new task variable and without the lengthy training typically necessary to train mice to withhold licking during the delays (Aruljothi et al., 2020; Gallero-Salas et al., 2021).

### Sensory stimulus presentation for baseline perceptual processing

**Figure 8.**
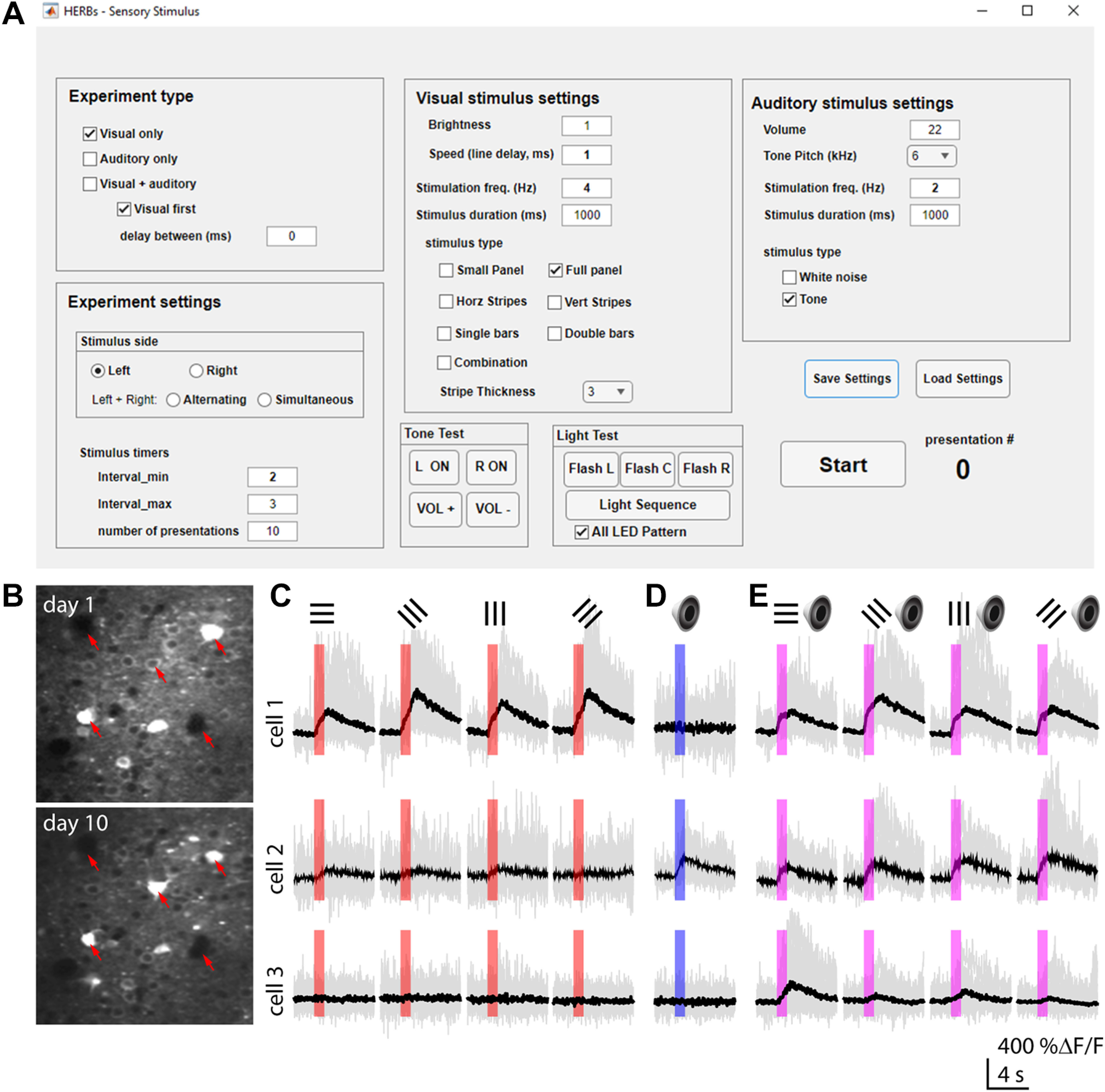
HERBs sensory stimulus presentation. ***A***, GUI for sensory stimulus presentation. ***B***, Example images of two-photon imaging of GCaMP6-expressing neurons in the PPC. ***C***, Example traces showcasing responses to visual stimulation in PPC layer 2/3 neurons. ***D***, Example traces showing auditory responses in PPC layer 2/3 neurons. ***E***, Example traces from the same cells as in ***C*** and ***D*** showing multimodal responses.

Our goal was to provide a simple GUI to display any of the stimuli used in our 2AFC and GNG tasks (Fig. 8*A*). To start the GUI add (with subfolders) the “Behavior_GUIs” folder to your MATLAB path (or cd to the “Behavior_GUIs” folder in MATLAB, launching the GUI will automatically add the relevant folders to the path) and type:

≫HERBs_Sensory_Stimulus

into the MATLAB command line and hit enter.

This GUI will allow users to measure neuronal responses to the stimuli used in behavioral tasks without training protocols or rewards, amounting to passive observation of the stimuli (Pho et al., 2018). Through the GUI, the experimenter can set the location, style, and frequency of the same stimuli, the interstimulus interval and the number of presentations (see detailed description in Extended Data 1: Software Documentation, section 10). Stimulus timing is sent to the data acquisition boards as square pulse waveforms to allow precise alignment of stimulus timing with neuronal recordings. The only requirement before an experiment is habituation to head fixation and the hardware. Following habituation, responses form the same field of view can be followed across multiple sessions (Fig. 8*B*). The GUI allows for measuring neuronal responses to various visual stimuli or a sequence of orientations (0°, 45° left, 45° right, and 90°) presented randomly (by checking the “Combination” box under “Visual stimulus settings”; Fig. 8*C*). Responses to auditory stimuli (Fig. 8*D*) or simultaneous visual plus auditory multisensory stimuli (Fig. 8*E*) can also be measured. For multisensory stimulation, the sequence of auditory and visual stimuli can be determined via the “Visual first” checkbox with an option to set a delay between the two using the “delay between (ms)” box.

## Discussion

Here we present a comprehensive, highly customizable, and affordable solution to run 2AFC or GNG behavioral tasks, or to present visual or auditory stimuli to head-fixed rodents. The system is equipped with three lick ports to facilitate active trial initialization (Marbach and Zador, 2017; Musall et al., 2019; Najafi et al., 2020) in 2AFC tasks, and motorized movement of the lick spouts for precise temporal segregation of behavioral phases. We demonstrate that mice can be efficiently trained in tasks on this system for psychometric measurements of categorical decision-making or delayed sensory discrimination. Every aspect of the tasks can be logged using commercially available data acquisition systems to aid precise alignment with neuronal recordings. A further advantage of our design is the use of affordable, off-the-shelf parts, with minimal (or no) need for custom manufacturing. We also provide a full, open-source software package to run 2AFC, GNG, or sensory stimulus experiments, coupled with a detailed description of the inner workings of the system for those who wish to modify it. HERBs was developed in MATLAB primarily because of the mature and reliable serial communication routines provided by the Instrument Control Toolbox. We recognize that MATLAB is not free software, which may limit accessibility compared with free programming environments like Python. However, the control of MATLAB over version compatibility and the enclosed nature of the environment make it superior to Python, where unpredictable module updates can cause issues in the future for inexperienced users. To mitigate the cost associated with using MATLAB, we also provide compiled code that allows HERBs to run via executables without purchasing MATLAB (see Extended Data 1: Software Documentation, section 1).

We are cognizant that HERBs is only one of a myriad available solutions for rodent behaviors. Perhaps the most versatile commercially available product similar to ours is the Bpod system (Sanworks). This is a modular, highly versatile solution, that will allow the user to build a behavioral apparatus that is similar to the one we presented (Marbach and Zador, 2017; Solari et al., 2018). The cost of the Bpod control system for an auditory, three-lickspout 2AFC paradigm in 2022 would be almost 60% more expensive than our complete system, and it will not include visual stimulation options, enclosure, moving spouts, mounting, and other necessary hardware (Table 1). Additionally, to operate the Bpod ecosystem, the user must familiarize themselves with the BControl environment (no programming skills needed; https://brodylabwiki.princeton.edu/bcontrol). This is a broadly used and highly recommended solution, but it is not a turnkey system by any means. Setting up experiments using Bpod will require significant time investment. Another highly recommended, open-source control solution would be using the bonsai visual programming language (https://bonsai-rx.org/); this, however, remains untested for most applications.

**Table 1:** Comparison of currently available solutions for head-fixed rodent behaviors

	HERBs	IBL setup	Bpod	PhenoSys	Neurotar	VR (Harvey Laboratory)
Capabilities						
State machine	Yes	Yes	Yes	Yes**	Yes**	Yes
Visual stimulation	Yes	Yes	Possible	Yes	Available*	Yes
Auditory stimulation	Yes	Yes	Yes	Available*	Available*	No
Reward	Yes	Yes	Yes	Available*	Available*	Yes
One spout	Yes	Yes	Yes	Available*	Available*	Yes
Three spouts	Yes	No	Yes	No	No	No
Moving spouts	Yes	No	No	Available*	No	No
Punishment	Yes	Yes	Yes	Available*	Available*	Yes
Data logging	Yes	Yes	Yes	Available*	Included**	Yes
Programming required (software base)	No# (Arduino, MATLAB)	No# (BControl, Bonsai, Alyx, Python)	No# (BControl)	OEM software** (unknown)	OEM software** (unknown)	No# (ViRMEn, MATLAB, Arduino)
Flexibility	High	Moderate	Very high	Moderate	Limited	High
Build instructions	Available	Available	No***	No***	No***	Available
Custom parts	Few/none	Few	All***	All***	All***	Few
Building time	∼40 h	Unknown	Unknown	Unknown***	Unknown***	Unknown
Cost (approximate)	$2600	$6500	$4100	$46,000	$30,000	$4000##
Cost includes mechanical parts	Yes	Yes	No	Yes	Yes	Yes
Cost includes PC	No	Yes	No	Yes	No	No

IBL, Image-based lighting; VR, virtual reality.

*Available from the manufacturer at additional cost.

**Manufacturer provides proprietary software for operation, experimental planning, and data logging.

***Manufacturer sends parts or preassembled products. Assembly, on-site installation, and/or training may be available at additional cost.

#All necessary software and installation guides are provided/available online. Installation, troubleshooting, or modifications might require competence in the noted languages.

##Cost estimated based on 2018 pricing.

More complex commercially available systems include rodent virtual reality setups (e.g., https://www.phenosys.com/products/virtual-reality) or the mobile home cage from Neurotar (https://www.neurotar.com/product/mobile-homecage). Some of these systems can massively expand experimental options (e.g., with virtual reality) but also come at costs in the range of tens of thousands of US dollars (Table 1). There are detailed instructions available online to DIY (do-it-yourself) build virtual reality systems (Thurley and Ayaz, 2017; Liu et al., 2021), including detailed instructions from the Harvey laboratory (https://github.com/HarveyLab/mouseVR; Pettit et al., 2022) and the Dombeck laboratory (http://www.dombecklab.org/wp-content/uploads/2021/01/Instruction-Manual-for-the-Smellevision.pdf; Radvansky et al., 2021). The complexity of these virtual reality systems, however, may deter those not well versed in DIY projects (Table 1). Another notable solution is created by the International Brain Laboratory consortium with extensive documentation on how to build and operate their apparatus (https://www.internationalbrainlab.com/tools; Aguillon-Rodriguez et al., 2021). Most of these solutions, however, will require considerable expertise in programming and electrical engineering, and potentially even access to a machine shop to produce custom parts. Furthermore, most DIY or commercial systems will limit the user to a specific task. Overall, there is currently no other solution for head-fixed rodent behaviors that is as comprehensive, easy to build, and operate, and as affordable as the one presented here.

We recognize and acknowledge that most systems neuroscience laboratories have already designed and built similar behavioral systems. All these solutions are highly capable, have already produced truly visionary experiments, and yielded insightful and critically important contributions to our understanding of the brain. Our goal was not to diminish these prior achievements. Rather, we present a solution suitable for newly starting laboratories or for those wanting to venture into the realm of systems neuroscience but who were thus far held back by the complexity or cost associated with the ecosystem necessary to run such experiments. We hope that disseminating an open-source and affordable solution will help overcome such barriers of entry and expand our community.

## Synthesis

Reviewing Editor: Mark Laubach, American University Decisions are customarily a result of the Reviewing Editor and the peer reviewers coming together and discussing their recommendations until a consensus is reached. When revisions are invited, a fact-based synthesis statement explaining their decision and outlining what is needed to prepare a revision will be listed below. The following reviewer(s) agreed to reveal their identity: Balazs Hangya.

Your manuscript has been reviewed by two experts in the field, and their reviews are given below in full. Please revise your manuscript to address all points that were raised. Most important, please provide source code, not the compiled Matlab functions, for the project. The paper cannot be considered for publication without access to the code. Also, it was reported that the GUI did not work for one of the reviewers. Please ensure that the programs run as expected across multiple operating systems and computers before posting the revised code. Thank you for sending your work to eNeuro.

Reviewer 1

First of all, I would like to congratulate the authors for the initiative of making their tool freely available to the community. We are currently facing significant challenges with reproducibility in experimental neuroscience. So, I understand that increasing transparency regarding protocols, software, hardware, and codes for data analysis must be a priority.

In the present manuscript, the authors describe a package for studying different aspects of rodent behaviors using a head-fixed approach. The authors claim that their package can be built at a relatively low cost and requires no program experience for the installation and usage of the graphical user interface. Besides, according to the authors, the hardware part of the package is comprehensive to assemble and handle during experiments.

The manuscript is well-written and, as I said, the initiative is very important within our current context. For this first round of revisions, I suggest the following modifications:

1-Make a new figure 1 providing carefully all the details of the step-by-step hardware assembling process.

2-Build a table to compare (side-by-side) their recently developed package with previously described tools. Use this table to improve the last 3 paragraphs of the discussion. Please add details of the costs of each tool in comparison with the new package.

3-In the introduction is very important to mention the problems of reproducibility our field is facing and how their package can benefit the community in this sense.

4-I suggest that the authors bring results of inter-laboratories reproducibility using the same package.

5-Regarding the comprehensive aspect of the hardware assembling, please provide more details about how time-consuming each available tool is in comparison with the recently developed package.

6-I suggest that the authors add some movies showing the software and hardware working.

7-Finally, I suggest that the authors give a name for the package and create a forum for users to receive feedback and solve questions. By the way, I found the software manual in the supplementary materials but did not find any additional material or tutorial for the hardware.

Please, discuss if making the GUI in Python instead of MATLAB would fit better (or not) in the author’s long-term goal (“We hope that disseminating an open-source and affordable solution will help overcome such barriers of entry and expand our community”).

Reviewer 2

In this manuscript, the Authors describe an easy-to-use and affordable behavioral unit capable of controlling three paradigms: 2-alternative forced choice, go-no-go and passive stimulus presentation. The Authors thoroughly demonstrate the use of the GUI-based behavioral testing unit. A valuable novel addition compared to previous open source solutions is a servo-mounted lickspout, which can be moved closed to and away from the mouse, thus separating trial phases and reducing a strong lick withholding component present in many implementations, which may otherwise complicate the interpretation of the results. Another addition is visual stimulus presentation using an LED panel, which inherits the easy and fast stimulus presentation by LEDs while also capable of featuring complex visual stimuli. These benefits make this setup valuable to publish. I have some comments to make the hardware-software package more accessible to the Readers.

Major points

1. I see the moving lickspout as one of the main benefits. A more thorough description of the feature would be desirable: how and with which type of sensor is the registration of licks carried out? Are all individual licks detected? Is this confirmed by grand truth video data? How does the lick spout move and at what speed? Does this make a sound? Does it interfere with recording and/or imaging? How can the moving of the lickspout be modified?

2. How are behavioral and recording/imaging data synchronized? Does this really take NI cards (which largely kills the ‘affordability’ argument)?

3. Figure 2: Detailed instructions for wiring are required if this setup is meant to be accessible for neuroscientists without special electric engineering expertise.

4. Original Matlab code should be provided and not only compiled mlapp files if the system is to be called ‘open source’.

Minor points

1. How was analgesia performed during surgery? Isoflurane has weak analgesic effect by itself, so it is recommended to use it with in the mixture of N2O and O2.

2. GUI parameters could also be saved in .xls on top of the .txt files to improve user experience.

3. Figure 1F: How was the sound attenuation performance calculated?

4. The Authors should test the system under different OS’s or remove the somewhat offhanded statement ‘we have no reason to believe that the system would have issues on Mac OS or Linux’.

5. Figure 5D: The representative fluorescent microscopy image of the injection site in the posterior parietal cortex needs to be described in more details, e.g. what are the different colors, where is the point of the injection exactly.

6. Figure 5E: What is the unit of measurement? A t-test on n of 3 is dubious statistically speaking; I suggest increasing the sample size or removing the test.

7. Can the distribution of the pre-stimulus delay period be controlled, like the post stimulus delay period?

8. Line 453, I don’t understand 1:6:6. Was it meant to be 1:1:6?

9. Figure 6B: It is unclear what happens after FA, hit and miss. Was the animal never rewarded? The ITI should probably follow from the behavioral feedback.

10. In the 2AFC paradigm, does licking of the middle spout always result in a reward? Or can this function be turned off?

11. How is the volume of reward calibrated?

12. Is sound intensity calibrated for auditory stimuli?

13. One does not need to code BControl in order to operate BPod: remove this statement.

14. 3D printing is not a real limitation, since this service can be ordered at affordable rates from companies (e.g. Shapeways) across the globe: please remove this argument. (At the same time, Amazon does not deliver to all countries while others may ban Amazon orders due to billing issues, so what the Authors consider ‘off the shelf’ may be very country-specific.)

15. Typos: Figure 5A, trail - trial; use μl instead of ul.

## Author Response

Synthesis Statement for Author:

Your manuscript has been reviewed by two experts in the field, and their reviews are given below in full. Please revise your manuscript to address all points that were raised. Most important, please provide source code, not the compiled Matlab functions, for the project. The paper cannot be considered for publication without access to the code. Also, it was reported that the GUI did not work for one of the reviewers. Please ensure that the programs run as expected across multiple operating systems and computers before posting the revised code.

Thank you for sending your work to eNeuro.

Response: We thank the Editor and our Reviewers for the timely turnaround on our manuscript and for their insightful comments. Here, we are including a copy of the revised manuscript will all changes highlighted (in red font) and a separate clean copy. All revisions have been completed as requested, please see our detailed responses below.

Reviewer 1

First of all, I would like to congratulate the authors for the initiative of making their tool freely available to the community. We are currently facing significant challenges with reproducibility in experimental neuroscience. So, I understand that increasing transparency regarding protocols, software, hardware, and codes for data analysis must be a priority.

In the present manuscript, the authors describe a package for studying different aspects of rodent behaviors using a head-fixed approach. The authors claim that their package can be built at a relatively low cost and requires no program experience for the installation and usage of the graphical user interface. Besides, according to the authors, the hardware part of the package is comprehensive to assemble and handle during experiments.

The manuscript is well-written and, as I said, the initiative is very important within our current context. For this first round of revisions, I suggest the following modifications: 1-Make a new figure 1 providing carefully all the details of the step-by-step hardware assembling process.

Response: We thank the Reviewer for highlighting this, it is a fair point. A comprehensive guide for building our system would be a very large figure, spanning multiple pages. Instead of including all that in Figure 1 as suggested, we added two new documents in the Extended Data accompanying the manuscript that contain detailed instructions on how to build the entire system (separate instructions for mechanical hardware and for electronics).

2-Build a table to compare (side-by-side) their recently developed package with previously described tools. Use this table to improve the last 3 paragraphs of the discussion. Please add details of the costs of each tool in comparison with the new package.

2

Response: We thank the Reviewer for this suggestion, we produced a comparison table and included it in our manuscript.

3-In the introduction is very important to mention the problems of reproducibility our field is facing and how their package can benefit the community in this sense.

Response: We now mention the importance of reproducibility in our introduction.

4-I suggest that the authors bring results of inter-laboratories reproducibility using the same package.

Response: While this is a very good suggestion, we do not have the resources to distribute and test our system in other laboratories prior to publication. We are actually working on this within UCI, but it will take some time until our colleagues build and test our system as it is of lower priority on their end. We do hope that publication of this manuscript will lead to more cross-laboratory testing.

Anecdotally, we work closely with the McNaughton lab here at UCI, where behavioral systems are built by the same engineer, using the same components. This indicates that the system should transfer well across laboratories.

5-Regarding the comprehensive aspect of the hardware assembling, please provide more details about how time-consuming each available tool is in comparison with the recently developed package.

Response: This is a very good point. We provide an estimate of building time for our rig in the comparison table requested by the Reviewer. Unfortunately, precise estimation of building time of other solutions would require us to purchase and build these other systems, which is not feasible in practice. Thus, we cannot provide estimates for building systems other than ours. Some manufacturers do provide system building service at a fee but whether these solutions are truly turn-key or require extensive debugging is unknown. Again, anecdotal evidence suggests that building a new system will be quite time consuming unless detailed instructions and pre-tested code are supplied, like in our case.

6-I suggest that the authors add some movies showing the software and hardware working.

Response: We have produced two video clips (found in the Extended Data) showing the three-spout (2AFC) and the single-spout (Go-NoGo) systems in operation.

7-Finally, I suggest that the authors give a name for the package and create a forum for users to receive feedback and solve questions.

3

Response: We thank the reviewer for this suggestion. We named our system HERBs for “Head-fixed Environment for Rodent Behaviors.

The Github page included with the package has a separate “issues” section. Posts in this section automatically send an email to our group so we can address issues in a timely manner. We will point this out in the manuscript.

By the way, I found the software manual in the supplementary materials but did not find any additional material or tutorial for the hardware.

Response: We have included a detailed building tutorial for both the mechanical and electrical components, in response to the first comment.

Please, discuss if making the GUI in Python instead of MATLAB would fit better (or not) in the author’s long-term goal (“We hope that disseminating an open-source and affordable solution will help overcome such barriers of entry and expand our community”).

Response: The primary reason for using MATLAB is the Instrument Control Toolbox providing a mature and reliable foundation for designing this system. Cross-version compatibility and toolbox updates are better controlled in MATLAB than in Python where dependence on a large number of libraries and modules can cause serious issues (e.g. regular updates to numpy within the Anaconda distribution can render an entire software package unusable, as we frequently experienced with software like Suit2P). These issues can be managed through the use of dedicated environments, but this might be beyond the abilities of inexperienced users. That being said, the free and open-source Python is a considerable advantage. To counter this potential upfront cost (if the user’s institution does not provide MATLAB access), we created executable files that can run the system without any need for MATLAB installation (only the MATLAB runtime environment is needed, which is provided freely by MathWorks and also included in our package). Naturally, we would be happy to assist any efforts to translate our system to Python in the future. We included this in our discussion.

Reviewer 2

In this manuscript, the Authors describe an easy-to-use and affordable behavioral unit capable of controlling three paradigms: 2-alternative forced choice, go-no-go and passive stimulus presentation. The Authors thoroughly demonstrate the use of the GUI-based behavioral testing unit. A valuable novel addition compared to previous open source solutions is a servo-mounted lickspout, which can be moved closed to and away from the mouse, thus separating trial phases and reducing a strong lick withholding component present in many implementations, which may otherwise complicate the interpretation of the results. Another addition is visual stimulus presentation using an LED panel, which inherits the easy and fast stimulus presentation by LEDs while also capable of featuring complex visual stimuli. These benefits make this setup valuable to publish. I have some comments to make the hardware-software package more accessible to 4 the Readers.

Major points 1. I see the moving lickspout as one of the main benefits. A more thorough description of the feature would be desirable: how and with which type of sensor is the registration of licks carried out? Are all individual licks detected? Is this confirmed by grand truth video data? How does the lick spout move and at what speed? Does this make a sound? Does it interfere with recording and/or imaging? How can the moving of the lickspout be modified?

Response: We completely agree with the Reviewer’s assessment. In response, we added a description of the touch sensor to the revised manuscript with more detailed build instructions in the Extended Data. We also set up a camera to collect “ground truth” lick data, which we compared to what was registered via our electronics and found good correspondence between the two, suggesting that each individual lick is detected with high accuracy.

We thoroughly tested the system with two-photon calcium imaging and found no interference from the electronics. Our lab currently does not employ in vivo electrophysiology, so we did not directly test whether the lick detection interferes with electrophysiology or not. Anecdotally however, capacitive lick sensors do not work well for electrophysiology recordings, generating a substantial artefact. This has, in fact, been tested with the same lick detection circuit in the McNaughton lab here at UCI (the same engineer develops their behavioral systems based on our designs) where they found that the capacitive lick detectors are not ideal for tetrode recordings. This potential shortfall is acknowledged in the revised manuscript, and we recommend using optical lick detectors that are available commercially.

We also added a detailed description of the moving spouts, including the speed, operational modes, necessary power sources, maintenance considerations, and the sounds generated by the two different options.

All the above-mentioned additions are now included in the revised manuscript including additional panels in Figure 1 (G and H).

2. How are behavioral and recording/imaging data synchronized? Does this really take NI cards (which largely kills the ‘affordability’ argument)?

Response: This is a great point. Our system does not specifically require NI boards, any data acquisition system will work. In our updated parts list, we now include the USB-1208FS PLUS device from Measurement Computing (also available from Digikey and other vendors), which is a great, affordable alternative for ∼$200. We opted for the NI Cards as they work well with our Wavesurfer acquisition software but the USB-1208FS PLUS also has free recording software (MCC DAQ Software) that will work just as well. It is also possible to integrate USB-1208FS PLUS with MATLAB but it is quite cumbersome so our recommendation would be to use the supplied MCC DAQ software instead.

5

3. Figure 2: Detailed instructions for wiring are required if this setup is meant to be accessible for neuroscientists without special electric engineering expertise.

Response: We completely agree with the Reviewer’s point. In response, we created two new supplementary documents that detail the building process for the mechanical hardware and the electrical components.

4. Original Matlab code should be provided and not only compiled mlapp files if the system is to be called ‘open source’.

Response: We have included .m file versions of the .mlapp applications (Soure_code_for_APPs folder in the main folder of the code in the Extended Data and on GitHub), which should make the code truly open-source. Additionally, to mitigate the potential upfront cost of having to purchase a MATLAB license (if the user doesn’t have it already), we created executable files that can run the system without the need for MATLAB installation (only the MATLAB Runtime environment is needed, which is provided freely by MathWorks and also included in our package).

Minor points

1. How was analgesia performed during surgery? Isoflurane has weak analgesic effect by itself, so it is recommended to use it with in the mixture of N2O and O2.

Response: We apologize for omitting this detail, analgesia was provided by 5 mg/kg meloxicam.

We included this detail in the revised text.

2. GUI parameters could also be saved in .xls on top of the .txt files to improve user experience.

Response: We added functionality to the package that saves all settings in a separate file in .xls format.

3. Figure 1F: How was the sound attenuation performance calculated?

Response: We apologize for omitting this detail, we included details of the measurement in the

Methods section. We also included similar details for measuring the sound of the linear actuators.

4. The Authors should test the system under different OS’s or remove the somewhat offhanded statement ‘we have no reason to believe that the system would have issues on Mac OS or Linux’.

6

Response: The Reviewer is of course correct, we should have never made such a statement without testing. As it turned out, MACs use a different routine to operate the communication port and we did not find an easy way to make the software cross-platform compatible.

Consequently, we removed the statement in question. We did test the system on 7 different PCs and laptops with different chip sets (multiple Intel and AMD platforms) to ensure that the program runs smoothly on Windows PCs (both Windows 10 and 11).

If the Reviewer intends to test the package, it should be noted that upon startup the program looks for the presence of each component. Consequently, running any of the GUIs without all major components present (eg. without connecting the light panels) will create an error and will not load the GUI.

5. Figure 5D: The representative fluorescent microscopy image of the injection site in the posterior parietal cortex needs to be described in more details, e.g. what are the different

colors, where is the point of the injection exactly.

Response: We included the requested details in the figure legend. Canula locations are disclosed in the Muscimol injection section of the Methods.

6. Figure 5E: What is the unit of measurement? A t-test on n of 3 is dubious statistically speaking; I suggest increasing the sample size or removing the test.

Response: We appreciate the Reviewer’s insight and agree with the comment about statistics.

Regrettably, we are currently not set up to increase sample size on this experiment, so we removed the statistical comparison. Figure 5E displays the fitting coefficients (“β” for slope and “l” for lapse rate, as described in the Analysis of behavioral data section of our Methods) measured in these experiments and consequently have no unit.

7. Can the distribution of the pre-stimulus delay period be controlled, like the post stimulus delay period?

Response: Yes, controllable pre-stimulus delay for 2AFC experiments was included in our original submission and it is controlled by the “Variable Stim Start Delay” box in the GUI, described in the last paragraph of the 2AFC section in Results. In the revised manuscript (and software), we have added new functionality to the Go-Nogo GUI to enable variable pre-stimulus intervals that work identically to the post-stimulus delay. Detailed description of how these work are included in the manuscript and in the Extended Data: Software documentation.

8. Line 453, I don’t understand 1:6:6. Was it meant to be 1:1:6?

Response: We understand the Reviewer’s confusion. We revised this section in the manuscript and in Extended Data: Software Documentation with a more straight forward description of 7 what the boxes mean and what numbers should go in there.

9. Figure 6B: It is unclear what happens after FA, hit and miss. Was the animal never rewarded? The ITI should probably follow from the behavioral feedback.

Response: We thank the Reviewer for noticing this omission. We have updated the flowchart in Figure 6B to reflect the reward structure of the task.

10. In the 2AFC paradigm, does licking of the middle spout always result in a reward? Or can this function be turned off?

Response: Whether licks on the Center Spout are rewarded or not is controlled by the

“Percentage of Center Rewards” box where the user can set the probability of rewarding licks on the Center Spout. We included this in the description of the GUI in the 2AFC section of the

Results.

11. How is the volume of reward calibrated?

Response: We simply use a P10 pipettor to measure the dispensed water reward, we have added a brief description of this process in the revised manuscript.

12. Is sound intensity calibrated for auditory stimuli?

Response: At the moment sound calibration is up to the user. This can be done quite easily with a sound level meter or a microphone. We included a brief description of this in the revised manuscript.

13. One does not need to code BControl in order to operate BPod: remove this statement.

Response: As requested, we removed this statement.

14. 3D printing is not a real limitation, since this service can be ordered at affordable rates from companies (e.g. Shapeways) across the globe: please remove this argument. (At the same time, Amazon does not deliver to all countries while others may ban Amazon orders due to billing issues, so what the Authors consider ‘off the shelf’ may be very country-specific.)

Response: We agree with the reviewer’s assessment and removed the statement regarding 3D printing as a limiting factor.

15. Typos: Figure 5A, trail - trial; use μl instead of ul.

Response: We thank the Reviewer for noticing these, we have corrected the typos.
