# Supplementary material for: A Comprehensive, Affordable, Open-Source Hardware-Software Solution for Flexible Implementation of Complex Behaviors in Head-Fixed Mice
Source: eNeuro. 2023 Jun 26;10(6):ENEURO.0018-23.2023. doi: 10.1523/ENEURO.0018-23.2023 (PMC10306125; doi:10.1523/ENEURO.0018-23.2023)

HERBs electrical parts assembly instructions

This document is a detailed guide for assembling the electrical components of your HERBs behavioral rig.

The approximate build time for the electrical parts of this setup is around 20-30 hours depending on experience.

Tools you will need

- Allen key set
- Soldering station
- Drill and drill bits (specified below)
- Screwdrivers
- Needle nose pliers
- Wire cutter
- Wire stripper
- A vise can be handy but not mandatory

Circuit diagram for the electronics.

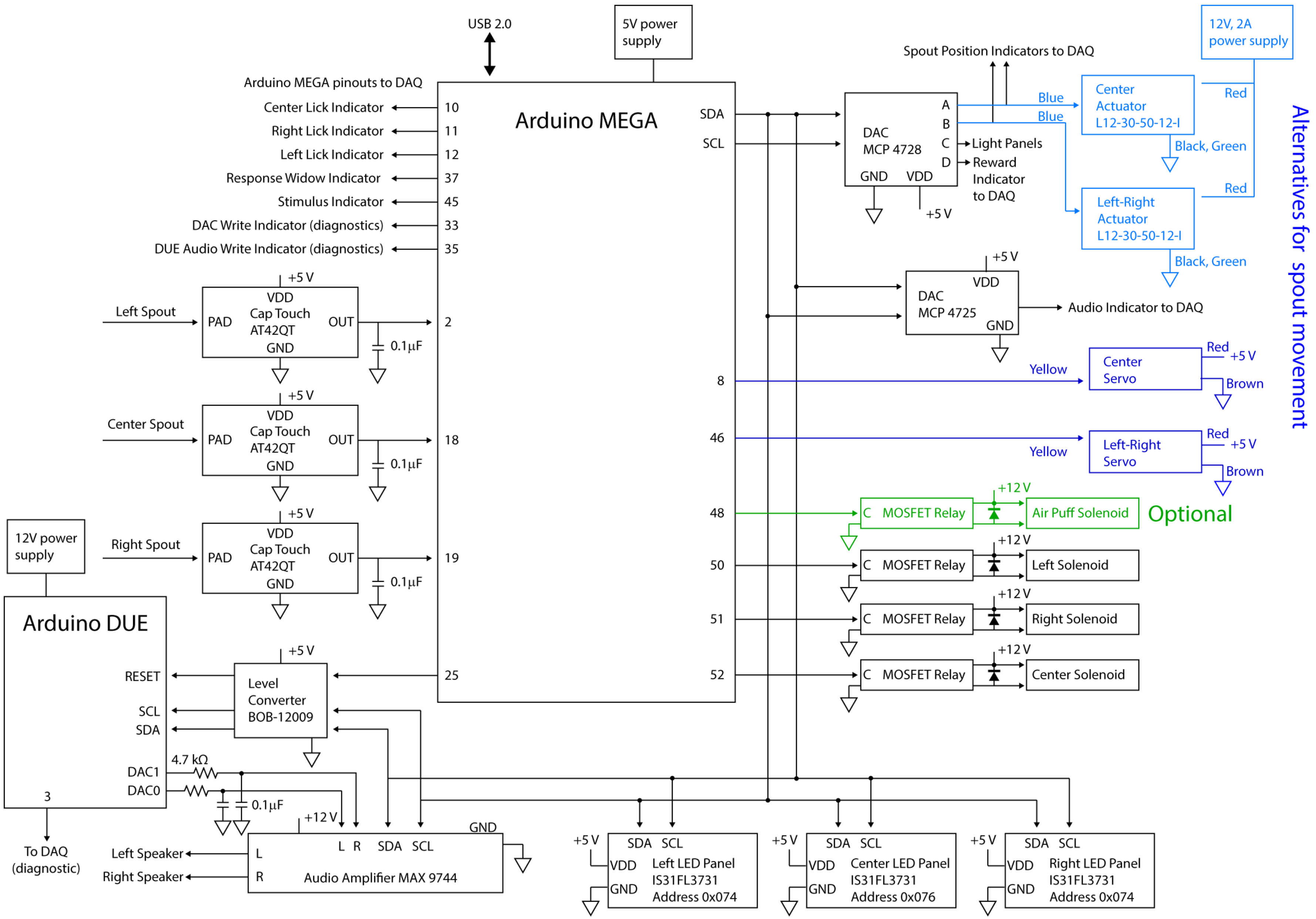

Initial board layout

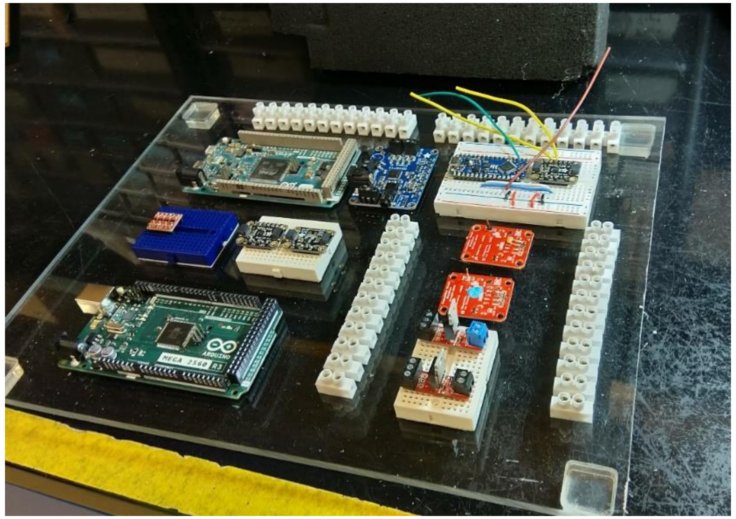

Preliminary layout shown with the parts on the plexiglass before drilling mounting holes.

Note that there are more than one way to create a good layout. As a general rule, it is advisable to try to keep wires as short as possible. However, this is not possible in all instances.

Any mounting surface will work for this, and mounting is not strictly necessary, but makes maintenance easier.

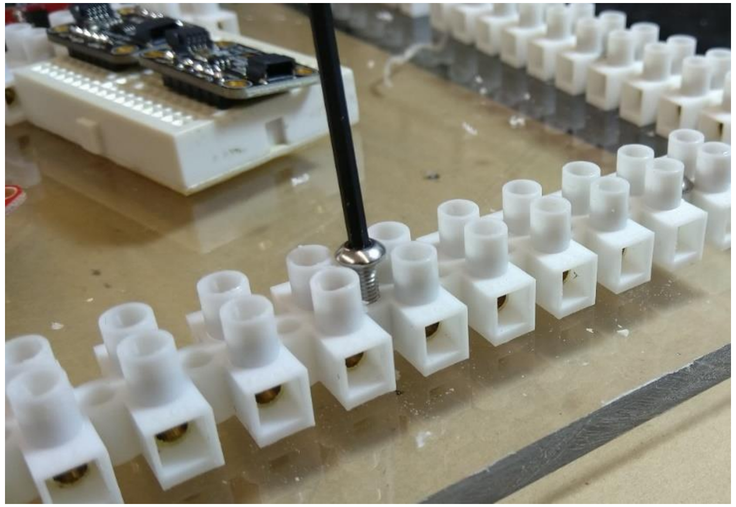

Mounting a connector block with an M3 screw into a predrilled hole.

Connector terminal blocks are not mandatory but using them makes both wiring and maintenance easier.

Attaching components to the base board

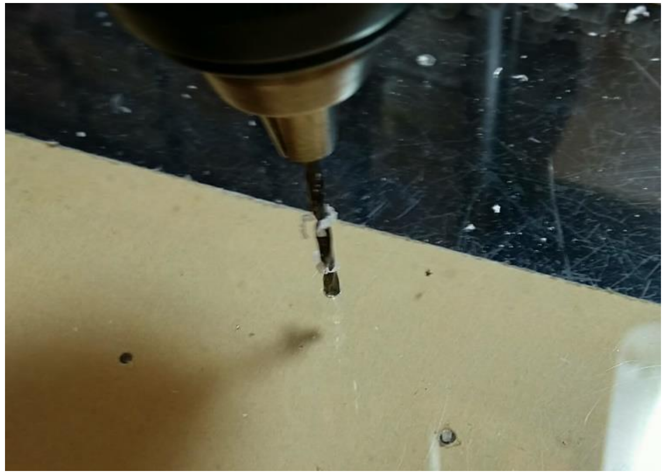

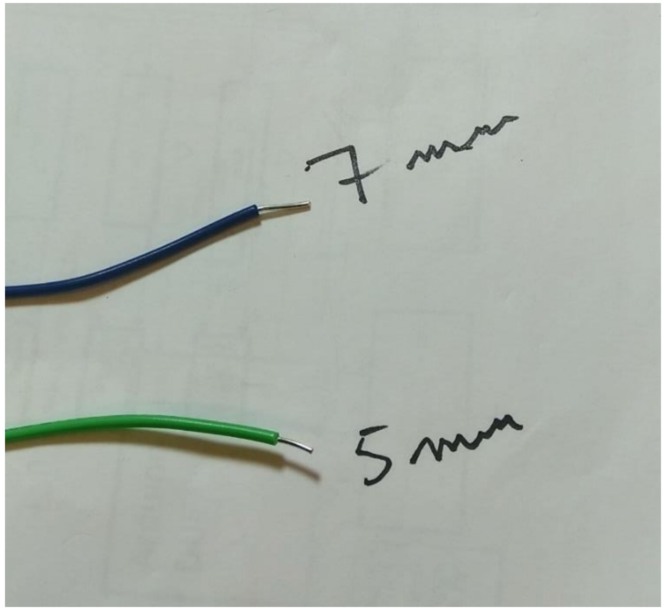

Screw types:
M3 Use 7/64 inch drill (about 2.7 mm)
M2.5 Use gauge 44 or 43 (about 2.1mm)

It is important to use correct sizes when drilling the holes into the plexiglass. This makes it possible to just insert the screws into the holes and have them “self thread” when screwing them in.

Use M3 screws for connector blocks and M2.5 for everything else.

Use 20 gauge solid wire. This makes it possible to make a solid connections to Arduinos by simply inserting the wire into the connector pinouts without soldering. Strip about 7mm of insulation off the end of the wire as shown in the picture. Be careful not to nick the wire, otherwise the wire may break when twisted.

Strip about 4-5mm of insulation off the wires that will be connected to the connector blocks. When inserting the wire into the connector block try to push the wire only to the point where the edge of insulation touches the edge of the internal metal part of the connector block. This is particularly important when two wires share the same connector on the same side. After inserting the wire(s) and tightening the screw use needle nose pliers and gently try to pull the wire out to ensure that all wires are seated properly.

Mounting power connectors

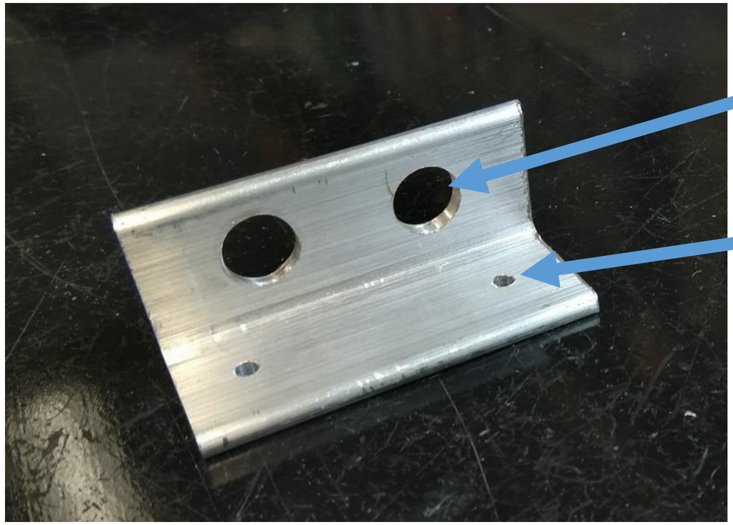

Power jack mount made from L-shaped aluminum bar for the 12V and 5V power supplies. Again, the assembly will work without this, but it makes installation easier.

Note the two mounting holes at the bottom for M3 screws

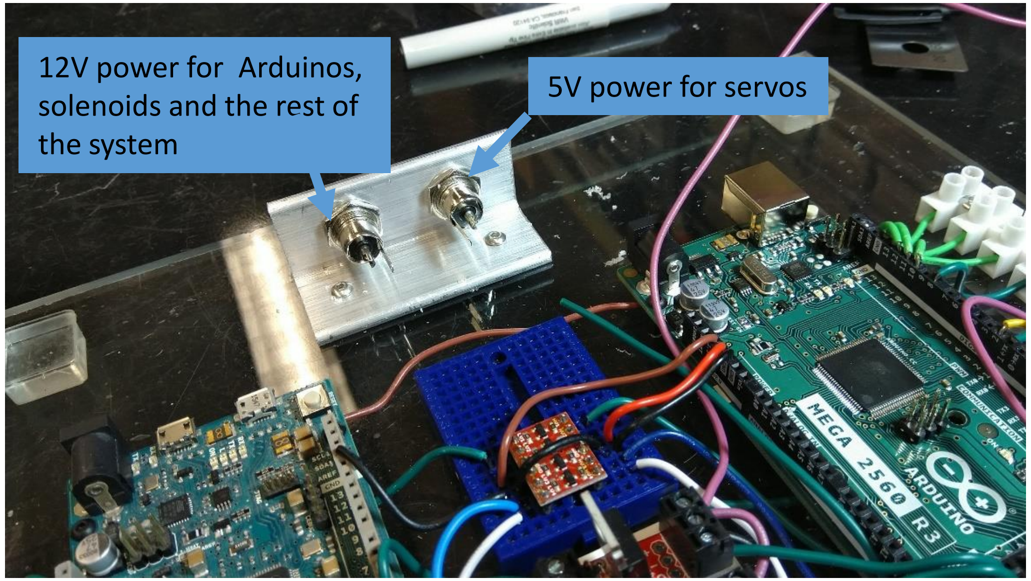

Power jack mount installed on the board before wiring has been completed.

It will be critical to label the two connectors: connecting the 12V power supply to the 5V system will fry the 5V electronics and damage some components irreparably.

Wiring up the power connectors

The power plug going into Arduino DUE
The black wire is ground, the red wire is the +12V

The 12V power plug that powers both Arduinos and the rest of the system with the exception of the servos

The 5V power plug that power the servos

The power plug going into Arduino MEGA
The black wire is ground, the red wire is the +12V

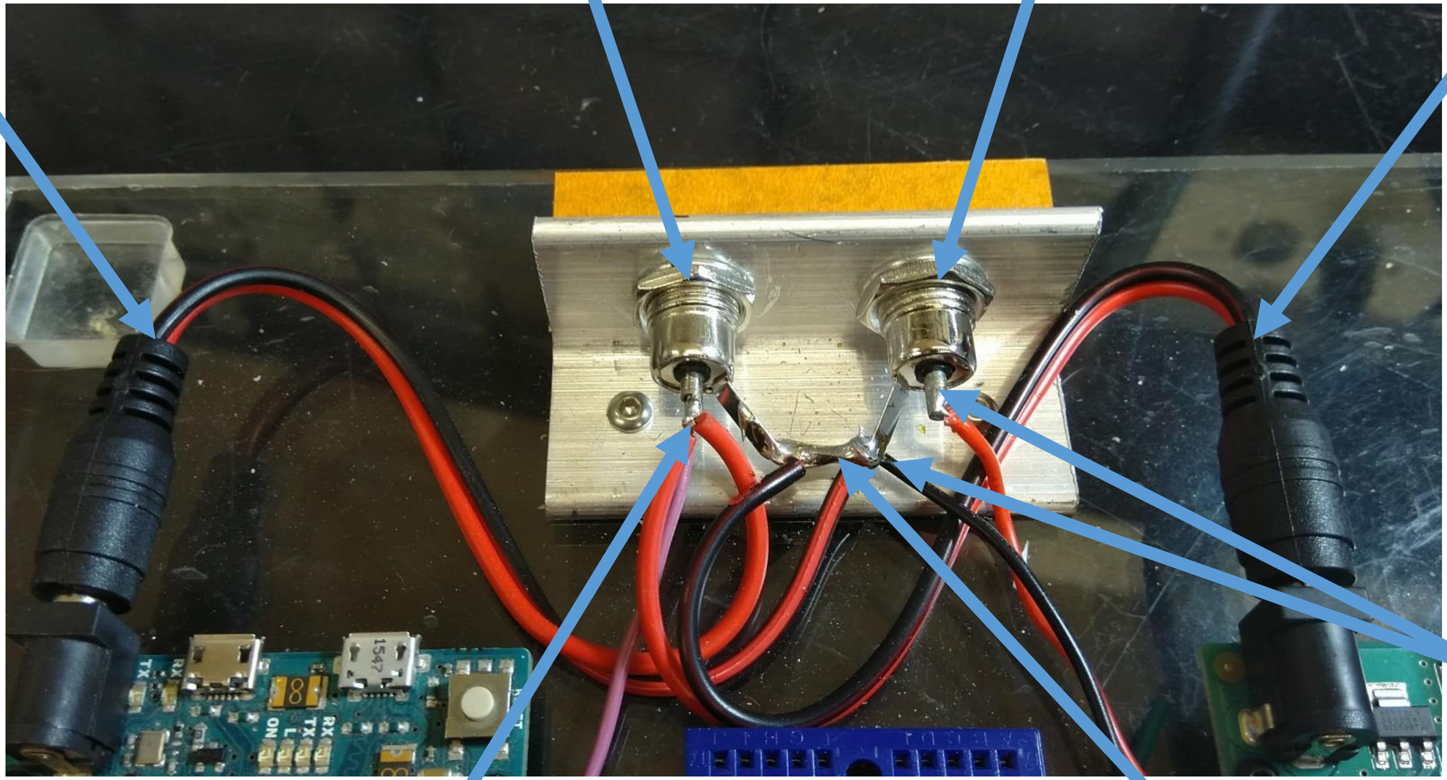

The positive (red) wires of the power plugs soldered to the positive terminal of the 12 V power jack.

The grounds from both power supplies soldered together here with the negative (black) wires of the power plugs

The power wire to the servos soldered here to the positive terminal of the 5 V power jack. The ground to the servos soldered to the common ground.
The +5V and the ground wires are connected to a connector block

Arduino MEGA pinouts

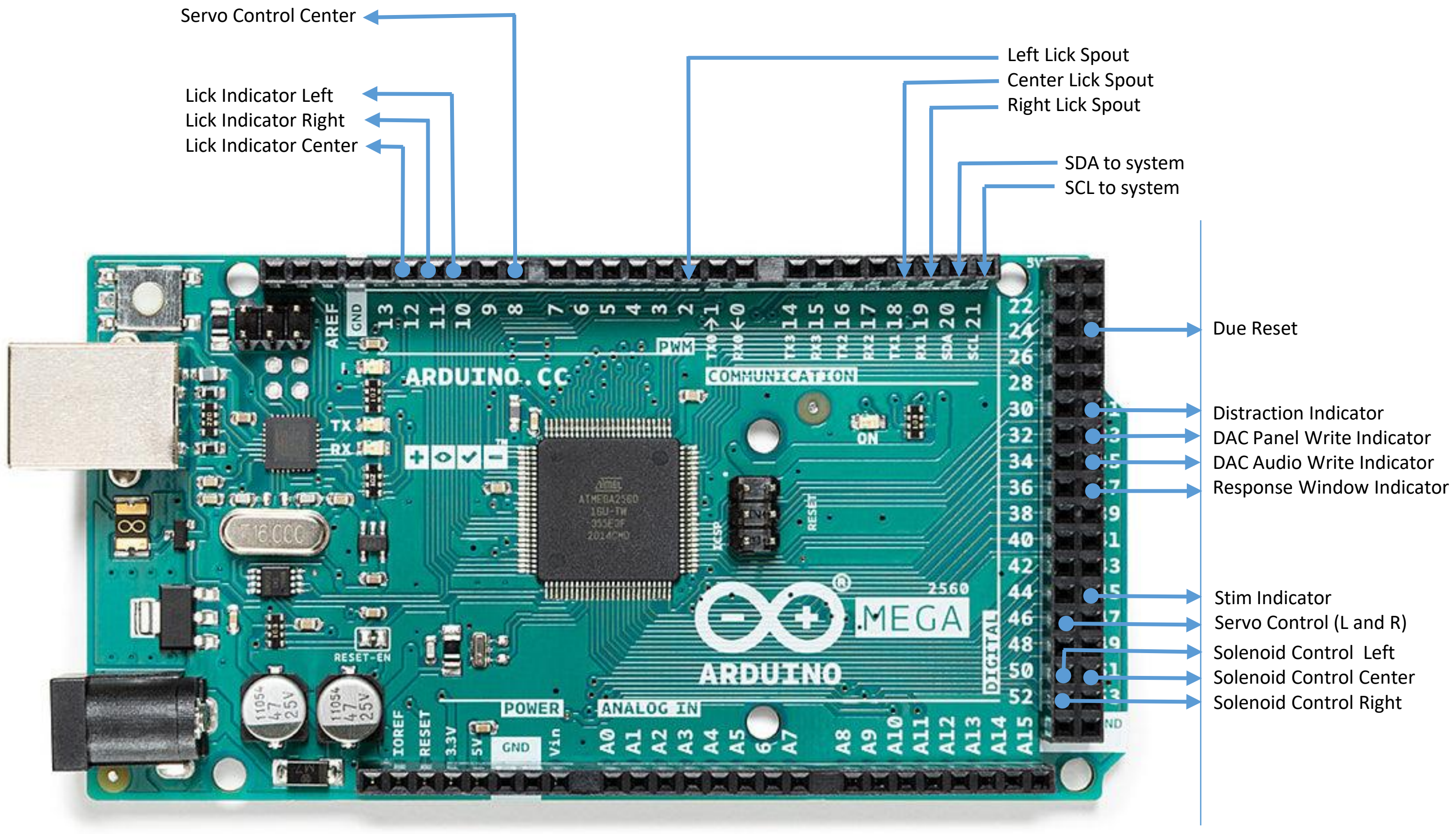

Arduino MEGA is the main input/output controller of the system.

It communicates through the USB-interface with the MATLAB program running on the PC.

It controls the servos, LED panels and audio operations and provides various status outputs to the DAQ.

Arduino DUE pinouts

Arduino DUE is used to generate the audio signals through its digital to analog converters (DACs). The DUE receives commands from the MEGA via SCL/SDA interface.

The DAC outputs are fed into the audio amplifier through a first-order low-pass filter.

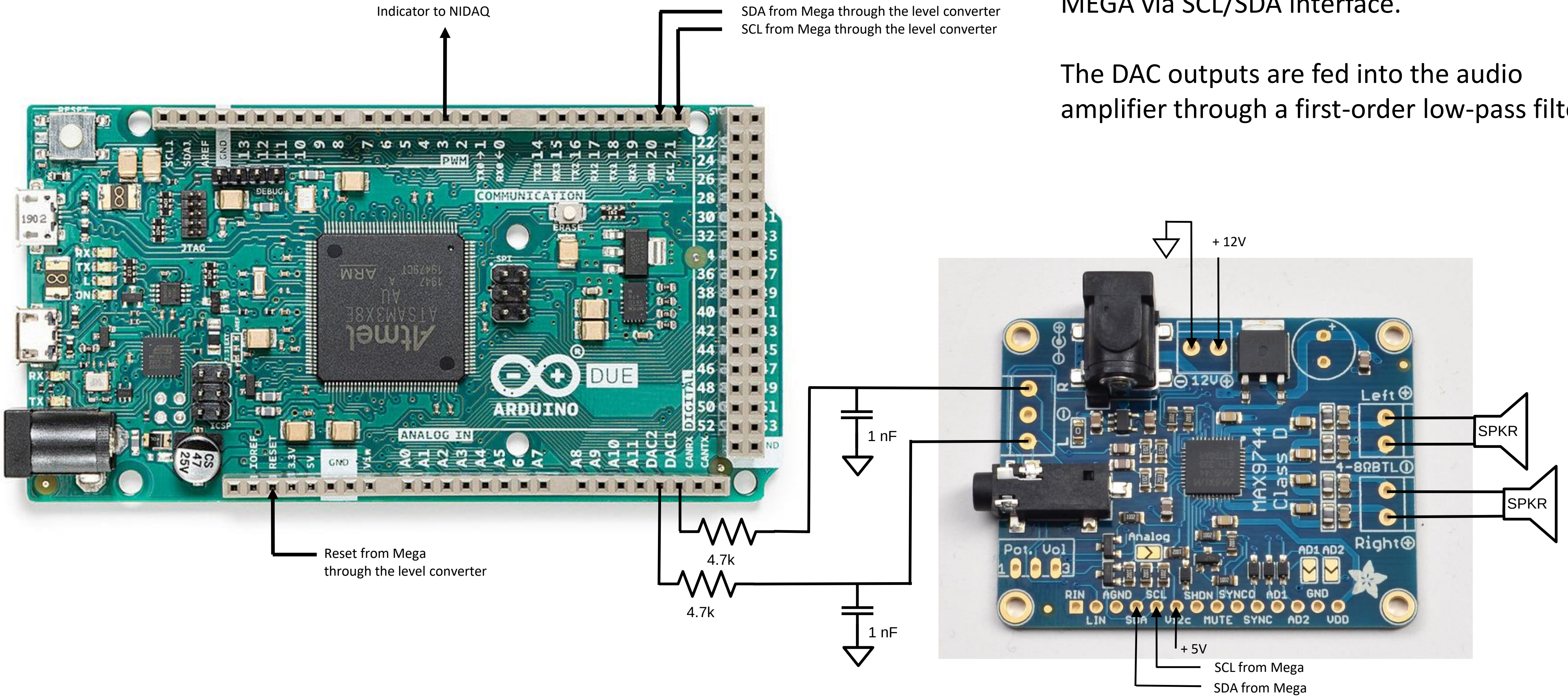

Wiring of the Audio amplifier (MAX9744)

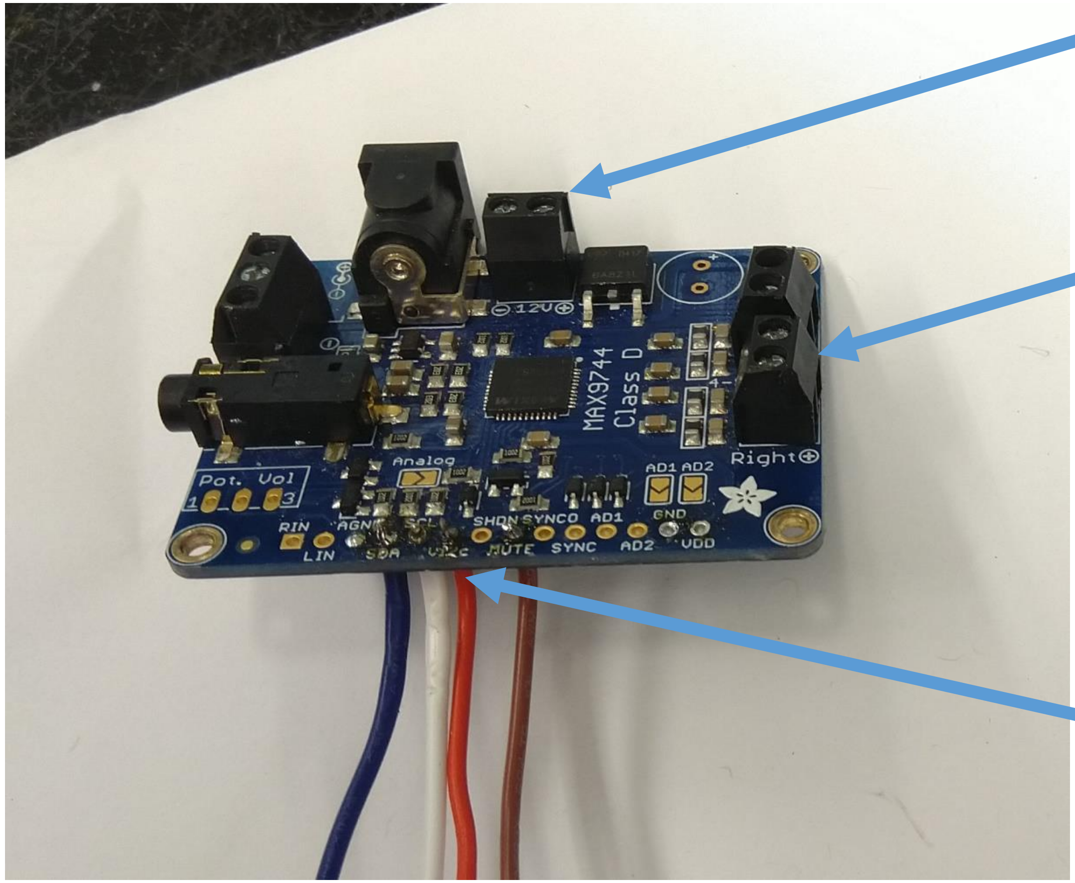

The ground and power (+12V) are connected through the screw connector

Left and Right speakers are connected here

The wires soldered to the audio amplifier board before mounting:

Blue SDA signal
White SCL signal
Red +5V for V2IC
Brown mute signal (to be used in the future)

Low-pass filter for audio

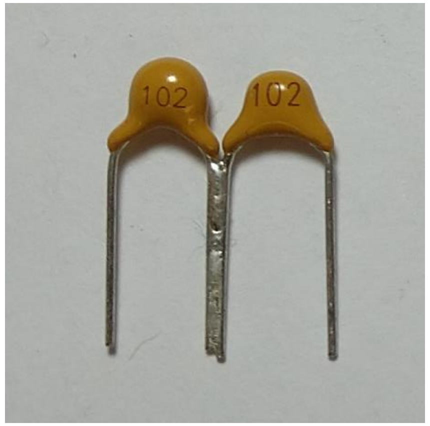

Two 1000 pF (1 nF) capacitors soldered together to be inserted into the input of the audio amplifier with two 4.7 k resistors to form a first order low-pass filter.

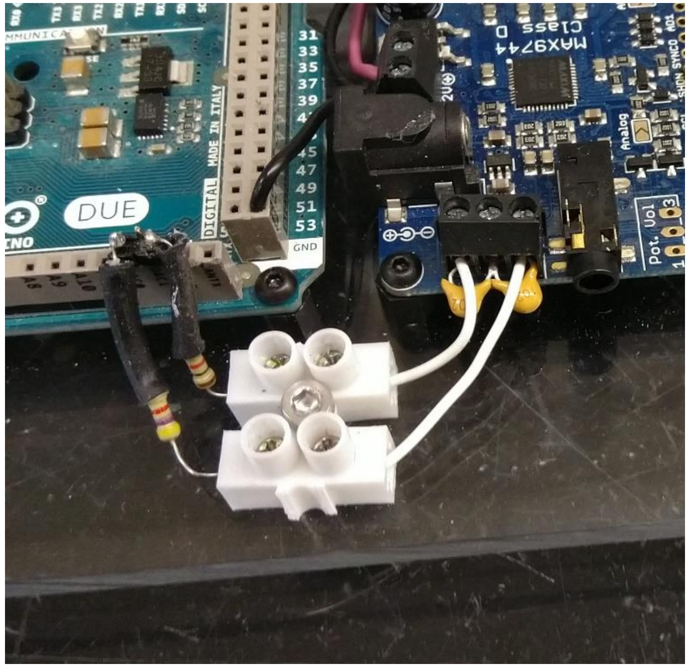

The two DAC outputs of the Due are each connected to the audio amplifier through a low-pass filter consisting of a resistor and a capacitor.

Level converter (BOB-12009) wiring

The Level Converter allows for communication between the 5V output of the Arduino MEGA and the 3.3V input of the DUE

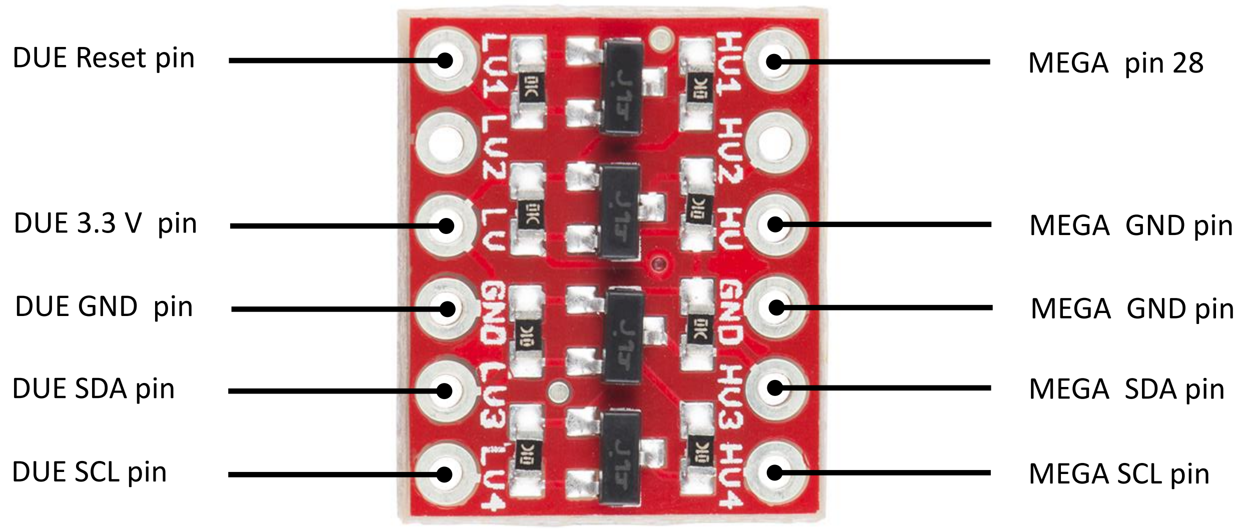

Inserting wires into the breadboards / Arduinos

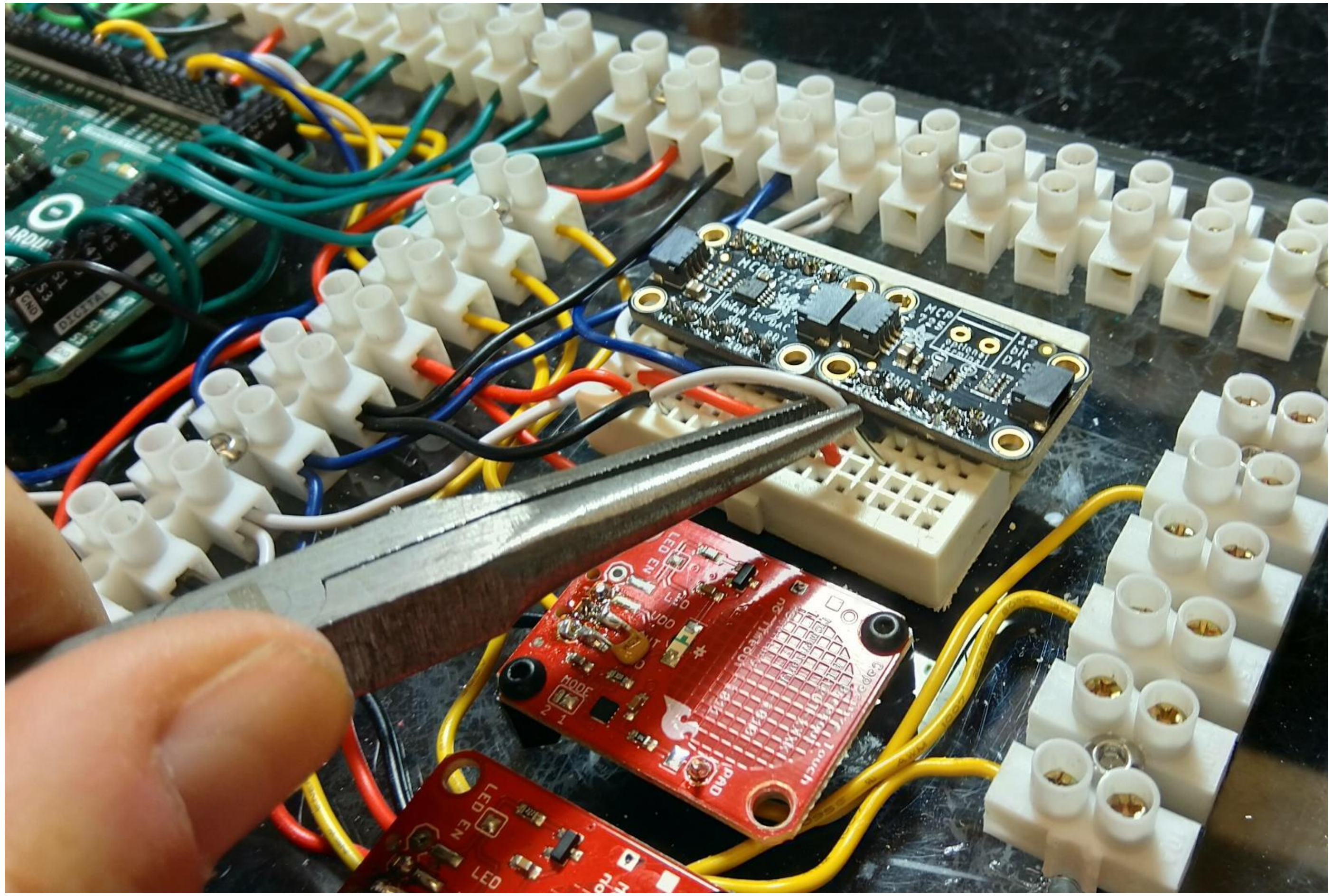

A good way to insert wires into breadboards and Arduinos is to use needle nose pliers as shown in the picture

Capacitate Lick detector (AT42QT-1010) wiring

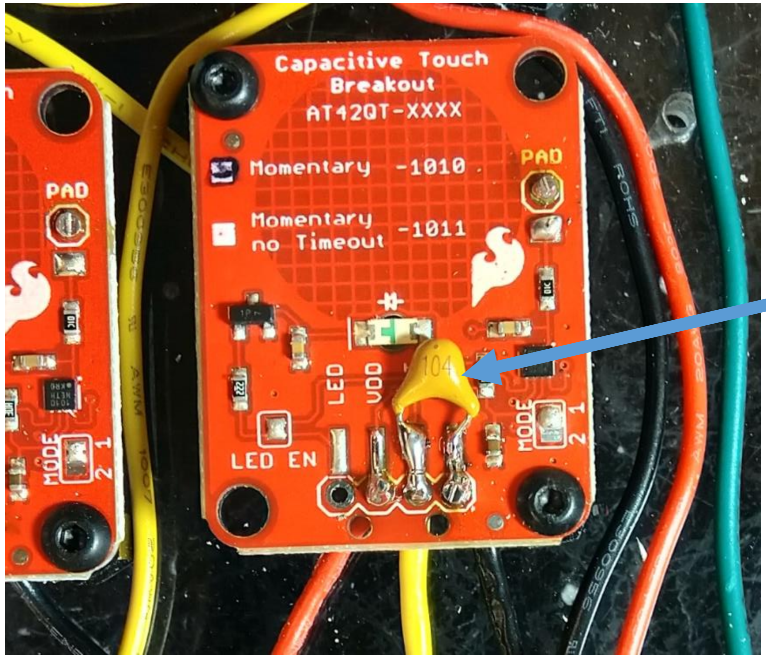

0.1 uF capacitor soldered between the ground and output pins of the capacitive lick detector board. The capacitor is needed to suppress repeated detection pulses that occur if the duration of the lick exceeds a certain time

Capacitate Lick detector (AT42QT-1010) wiring (continued)

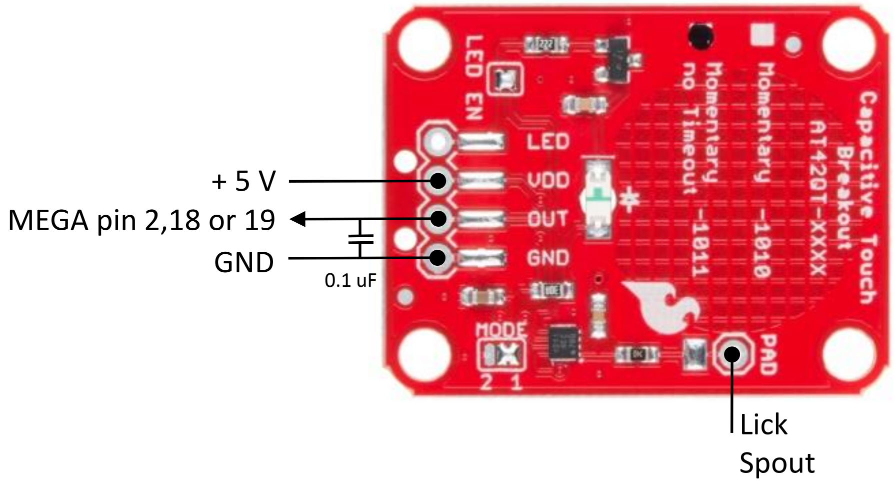

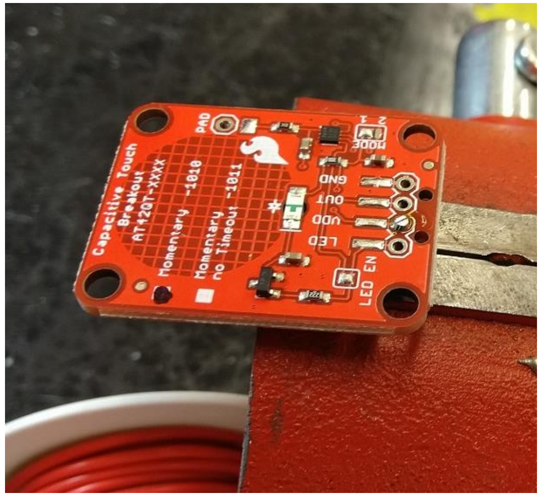

The +5V wire soldered using a vise to hold the wire

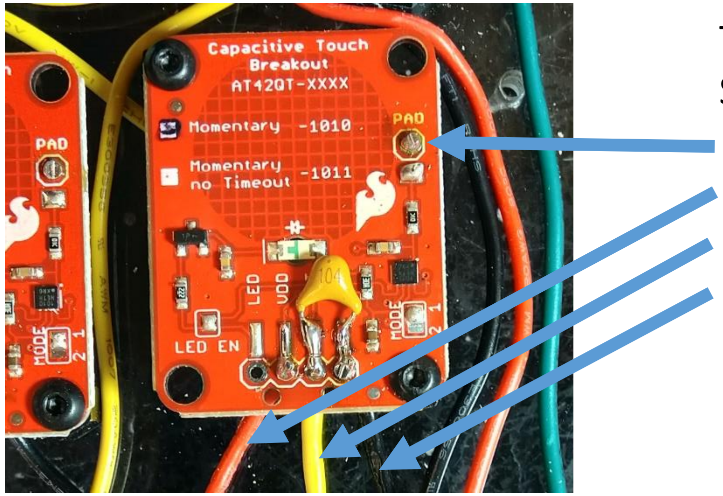

The 0.1 uF capacitor soldered between the ground and the output pin.
See the wires soldered to the board from below:

- Pad wire runs to the lick spout via the connector block.
- + 5V (red)
- Output (yellow)
- Ground (black)

MOSFET Switch wiring

The original power control kit from Sparkfun (COM-12959) is currently unavailable. If the user manages to buy the original part, the below detailed wiring applies but it is managed via screw terminals on the power control kit, making it a bit easier to assemble. Here we show how to build the switches from available parts (which is also the cheaper option).

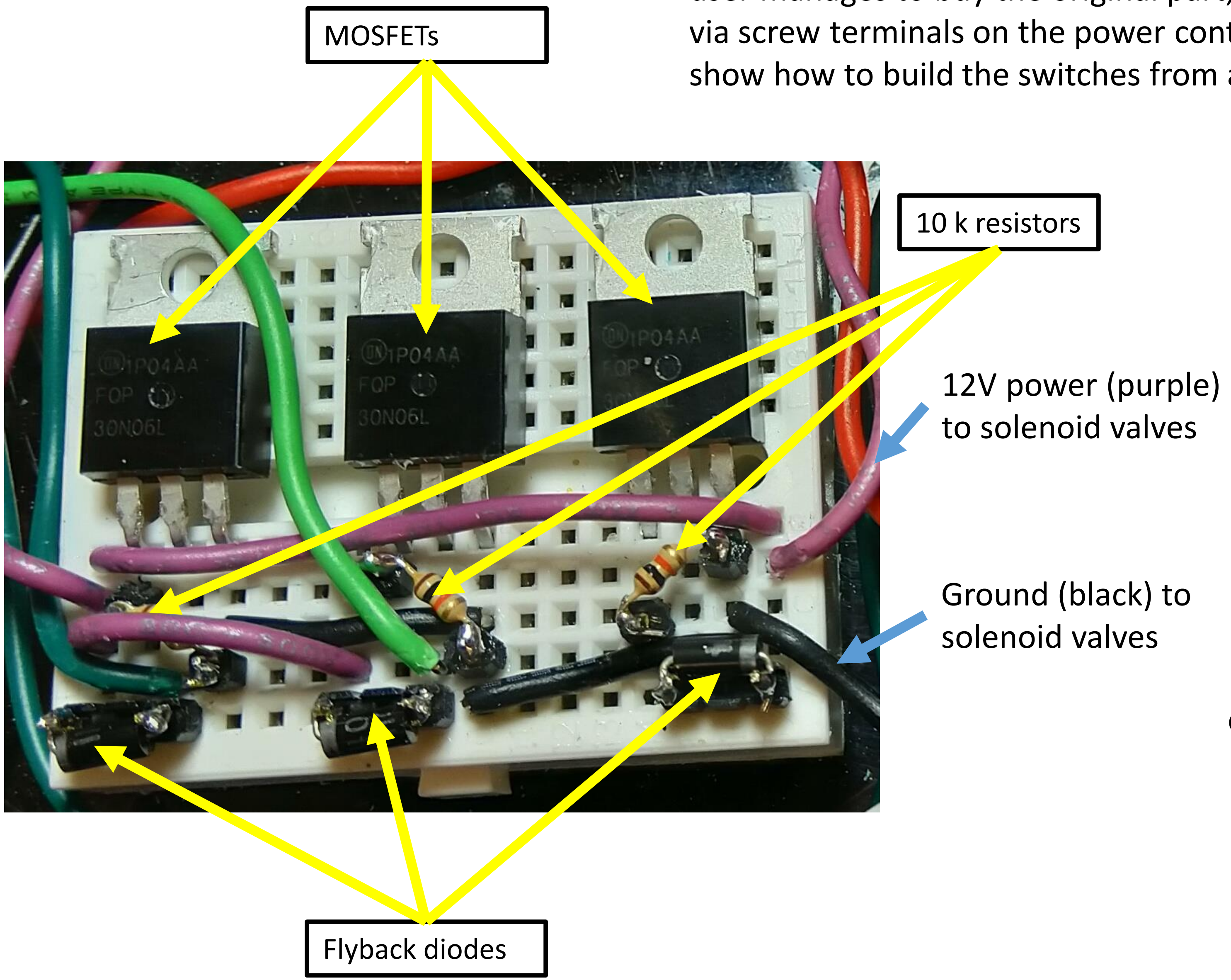

MOSFET Switch Circuit (single)

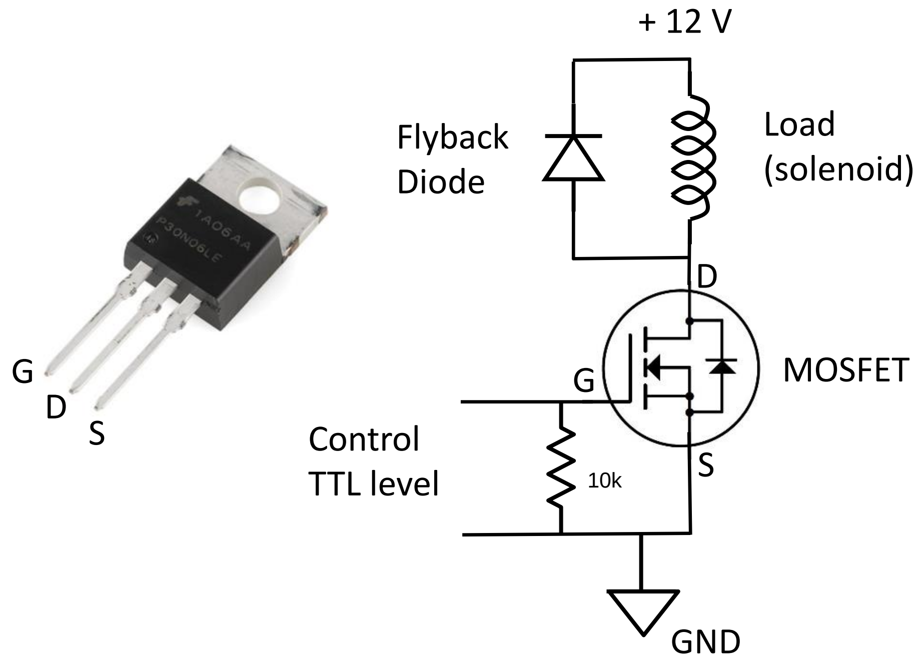

Digital to analog converter (DAC) wiring

Digital Analog Converters (DACs) are used for controlling the linear actuators (if the user decides to go with these over rotary servos) and to generate behavioral outputs sent to the DAQ board for monitoring.

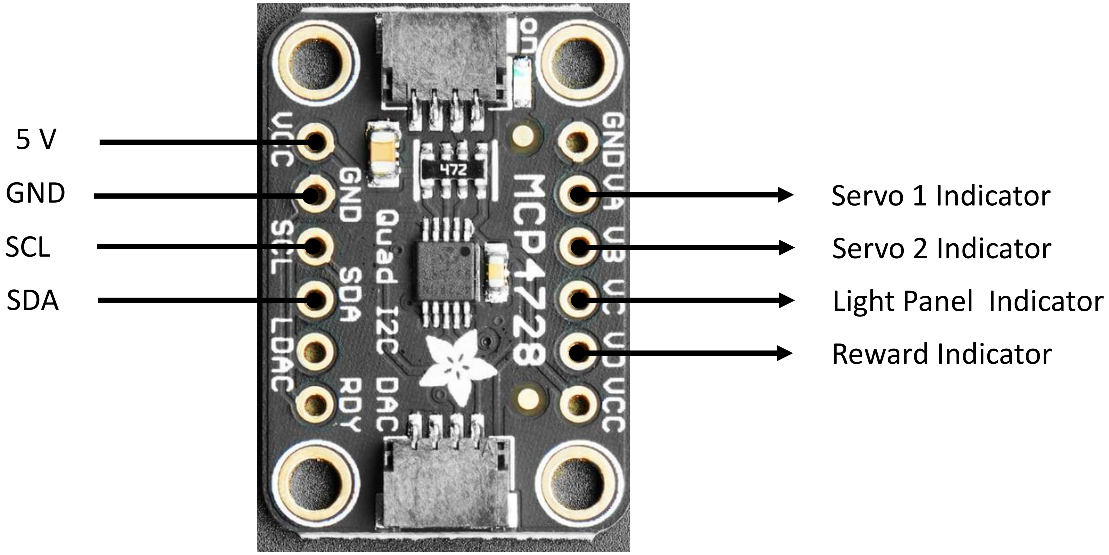

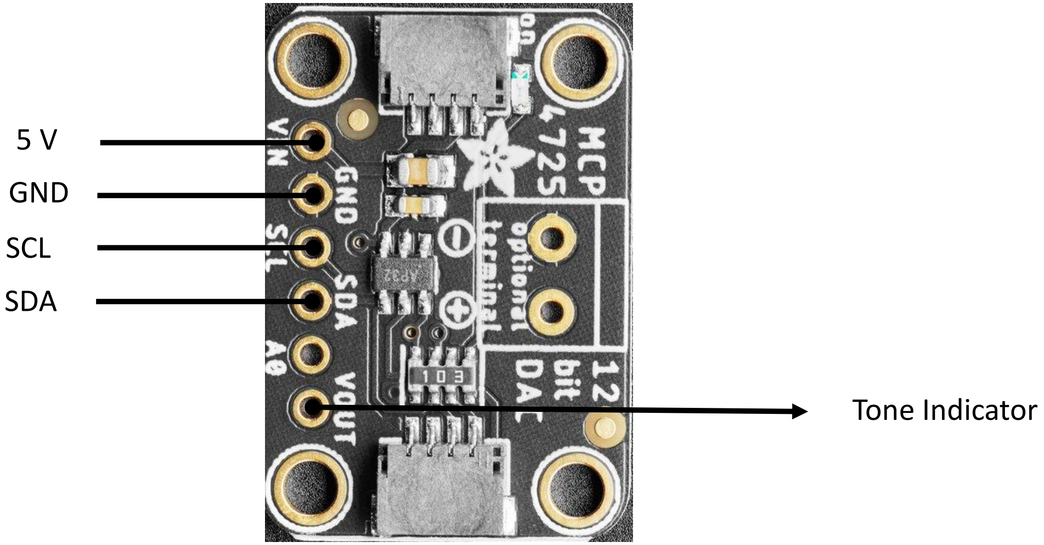

Final Board Layout

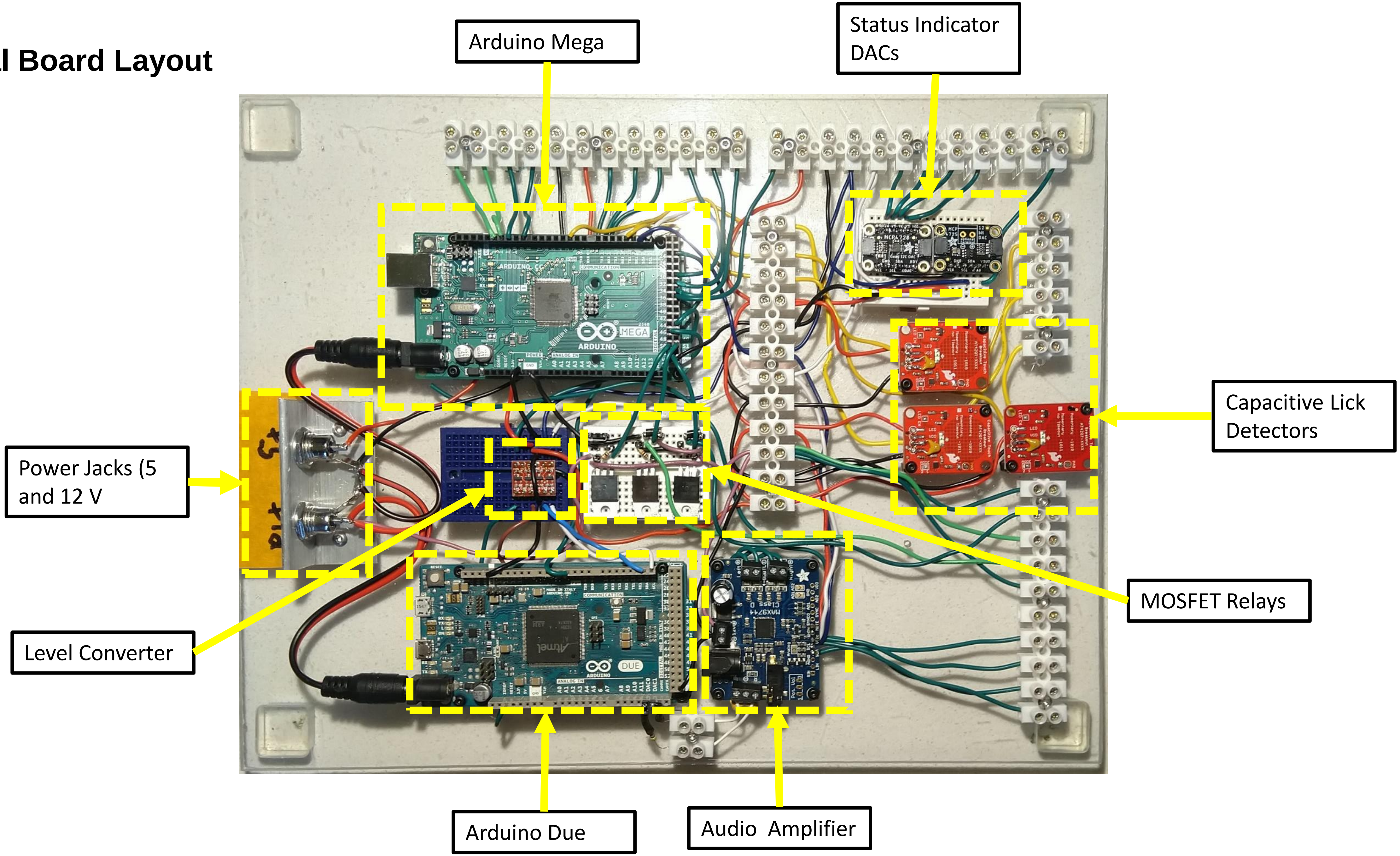

Supplement: Extended Data 1 — Extended Data containing 3D files, all necessary code, example video clips, parts list, detailed build instructions and full software documentation. Download Extended Data 1, ZIP file. [file enu-eN-MNT-0018-23-s02.zip › HERBs electrical hardware build instructions.pdf]
