# Supplementary material for: A Comprehensive, Affordable, Open-Source Hardware-Software Solution for Flexible Implementation of Complex Behaviors in Head-Fixed Mice
Source: eNeuro. 2023 Jun 26;10(6):ENEURO.0018-23.2023. doi: 10.1523/ENEURO.0018-23.2023 (PMC10306125; doi:10.1523/ENEURO.0018-23.2023)

HERBs mechanical parts assembly instructions

This document is a detailed guide for the assembly of the mechanical components of your HERBs behavioral rig.

The approximate build time for the mechanical parts of this setup is around 6-8 hours.

Tools you will need

- Allen key set
- A Dremel tool or saw (unless you buy your D-profile shaft pre-cut to size)
- Soldering station
- Superglue and/or 5-minute epoxy and applicator (tootpick)
- Drill and drill bits
- Screwdrivers
- Servo tester
- Needle nose pliers
- Wire cutter
- Wire stripper
- Zip ties
- A vise can be handy but not mandatory

Running disc

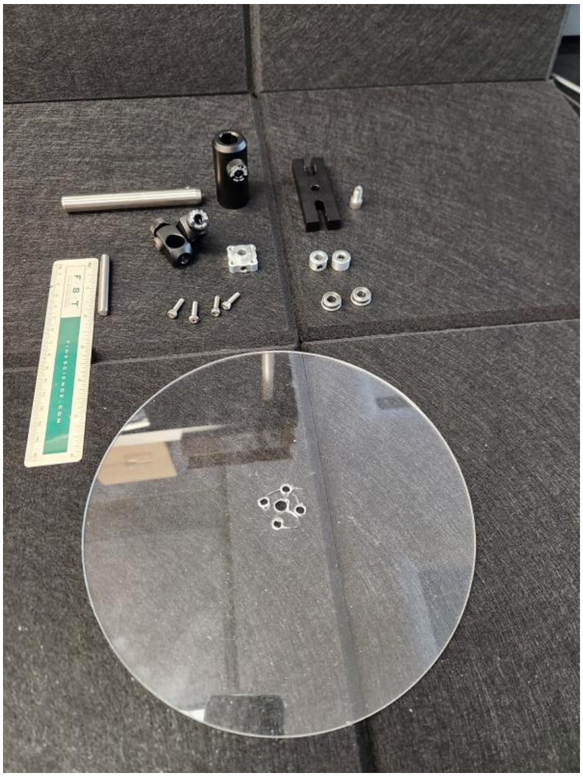

1. Parts
    8-inch diameter acrylic disc
    Axle is ¼ inch D-shaft
    Screws are 6-32
    Drill a hole in the center of the wheel

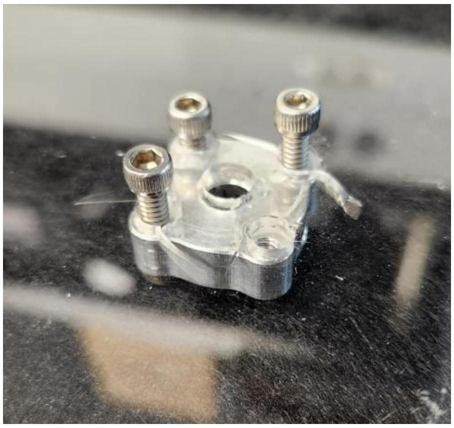

2. Use the hub to mark and drill the holes for the screws around the center. Attach the hub, leaving the screws lose until the axle goes in makes assembly easier

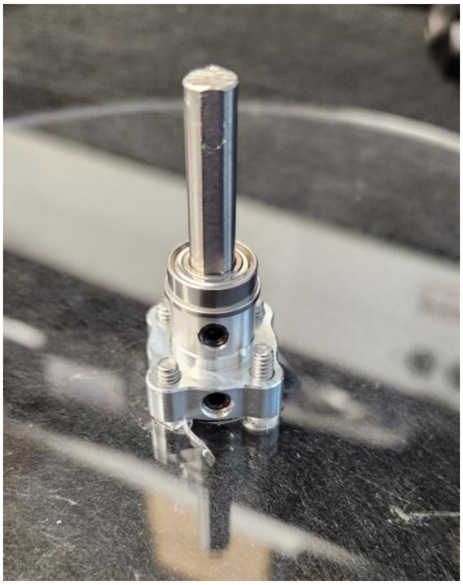

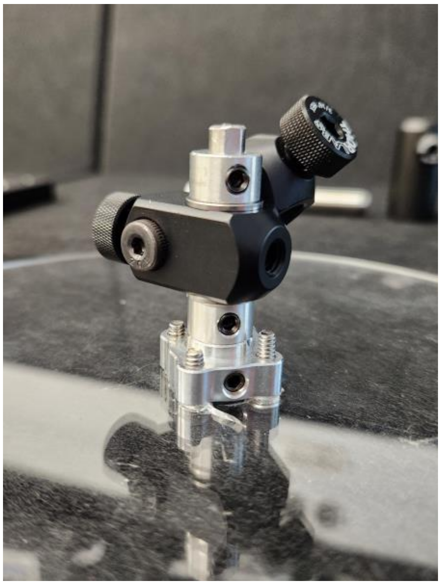

3. Insert the shaft, tighten the setscrew and the hub screws, then add a shaft collar and flanged ball bearing. Add the rotating clamp and the second ball bearing and close off with the second shaft collar.

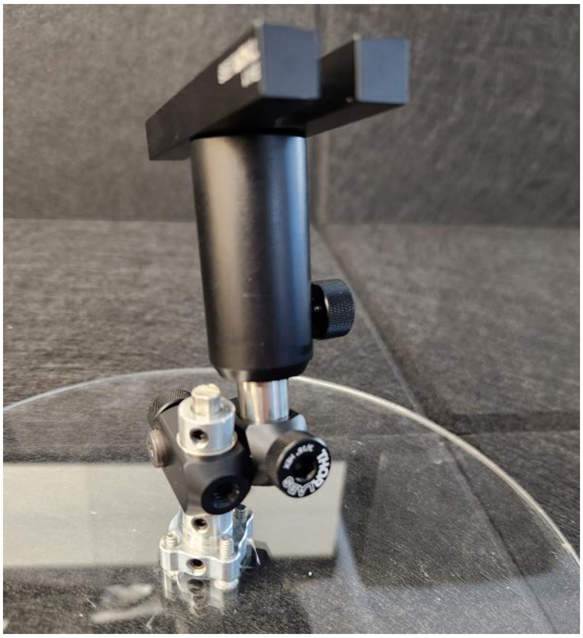

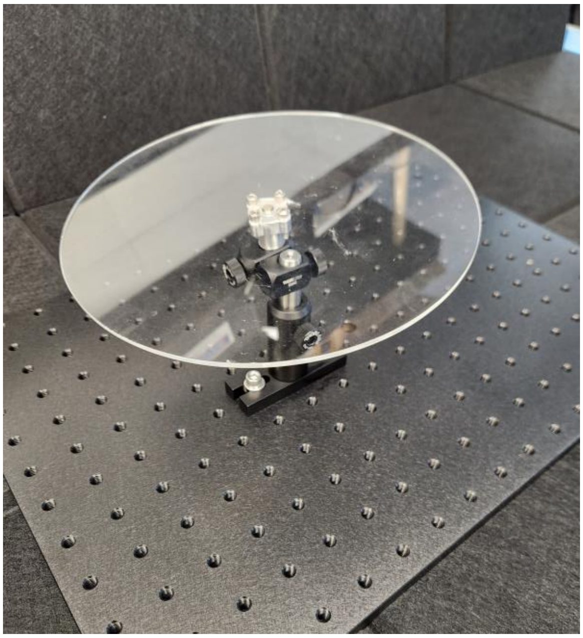

4. Assemble the post holder and secure it in the rotating clamp. Place and secure disc assembly on the breadboard with a ¼ 20 screw

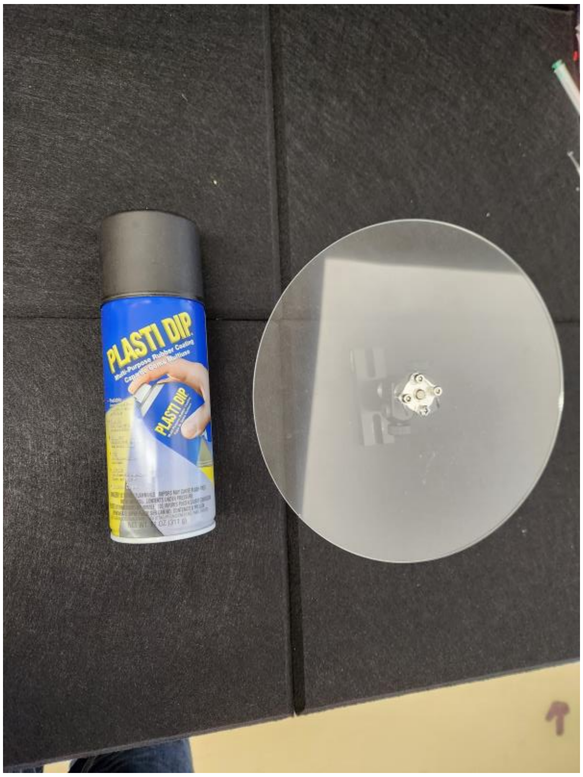

5. Coat the disc with Plasti Dip (or your choice of any surface coat. Apply 2-3 coats, with 4-5 hours of wait time (see manufacturers specifications) at least between each layer. The purpose of this is to make the surface less slippery for the animal.

For the purposes of this manual, we will not coat the disc yet to allow visibility of parts under the wheel on photographs.

Headplate holders

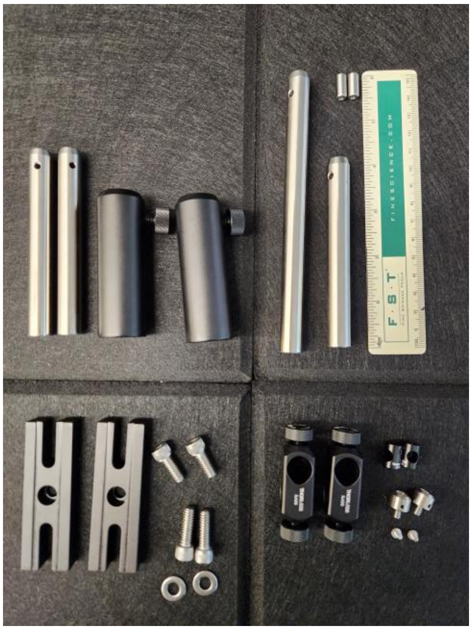

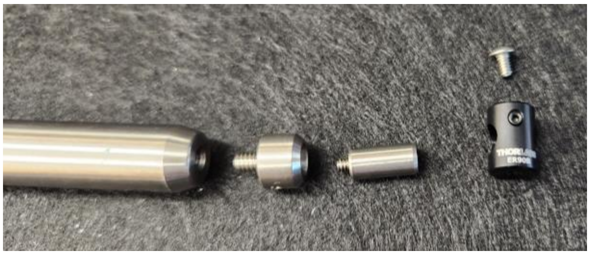

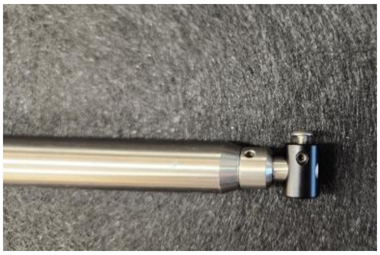

1. Parts: standard optomechanical parts from Thorlabs

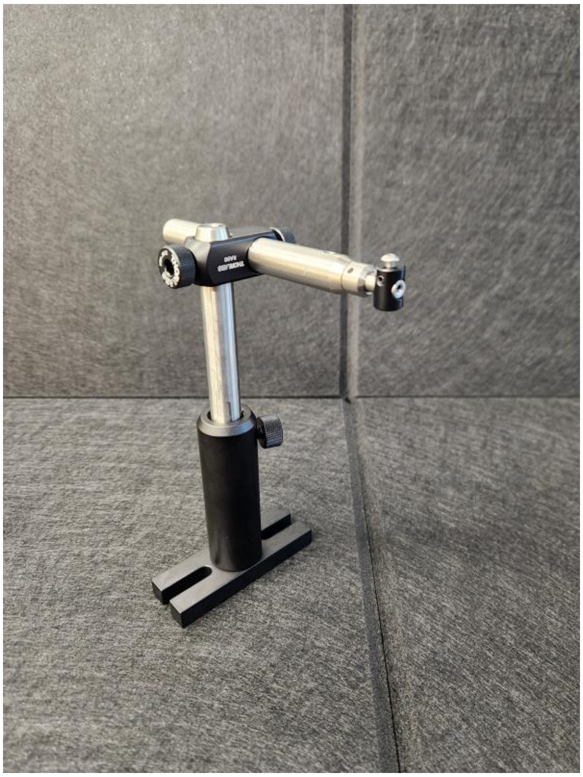

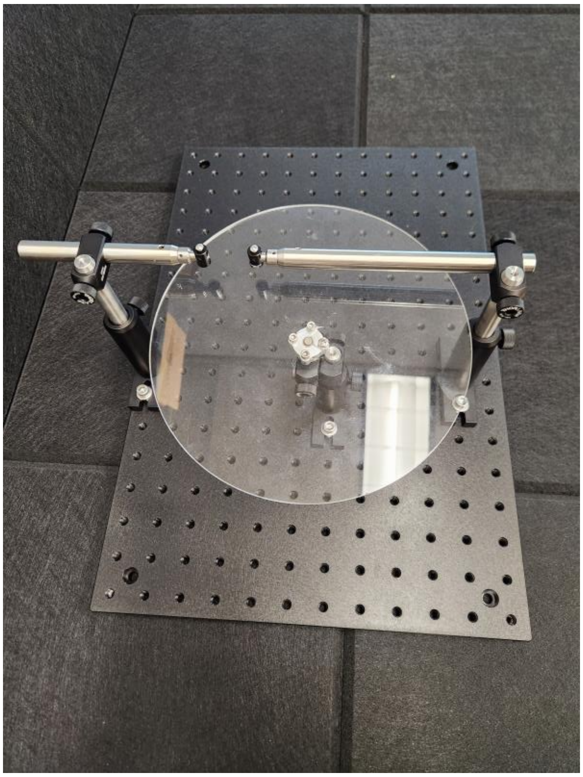

2. After assembly, secure the holders to the breadboard.

Visual Stimulus – LED panels

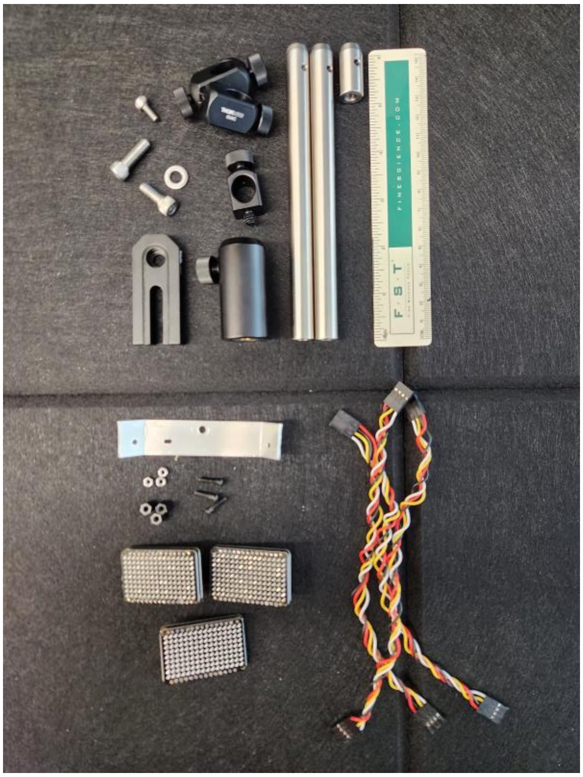

1. Parts
    - LED panels
    - optomechanical parts
    - bent aluminum or 3D printed LED panel holder
    - Jumper wires

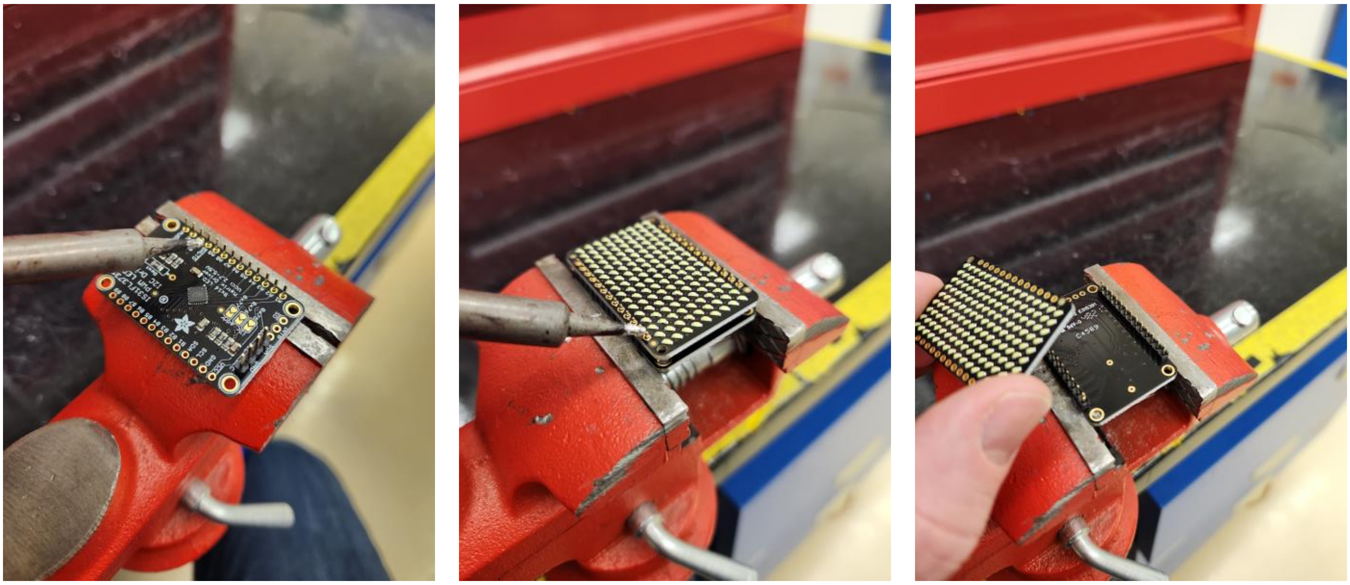

2. Solder the LED panel to the driver using the provided pins

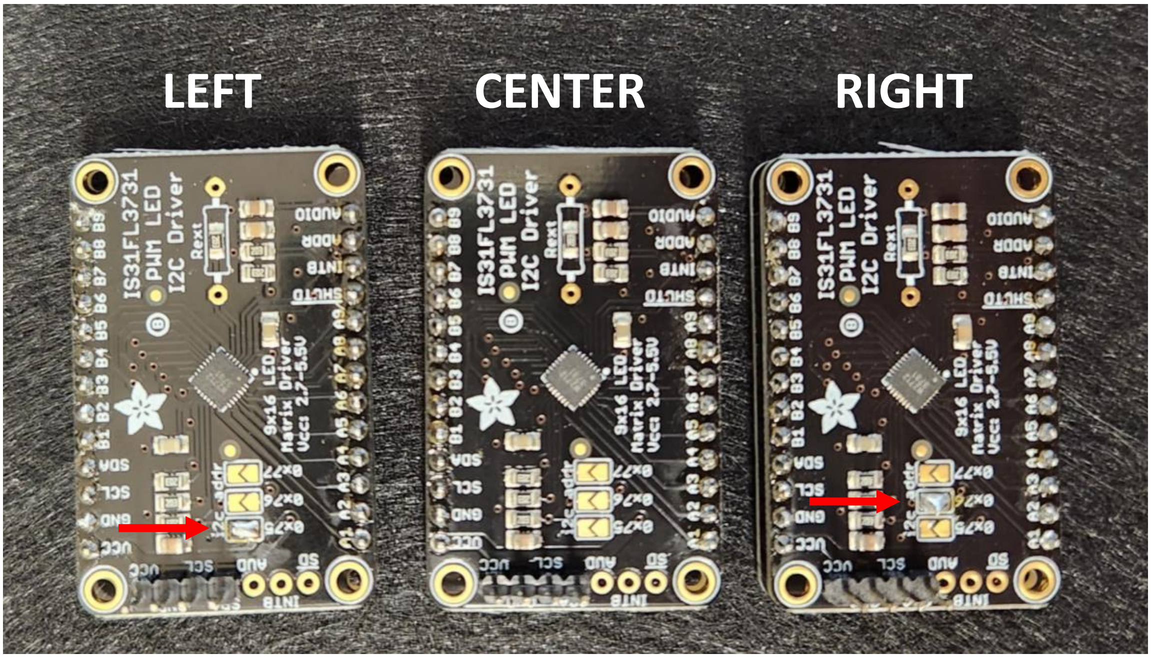

3. Solder the address panels on the driver (red arrows)

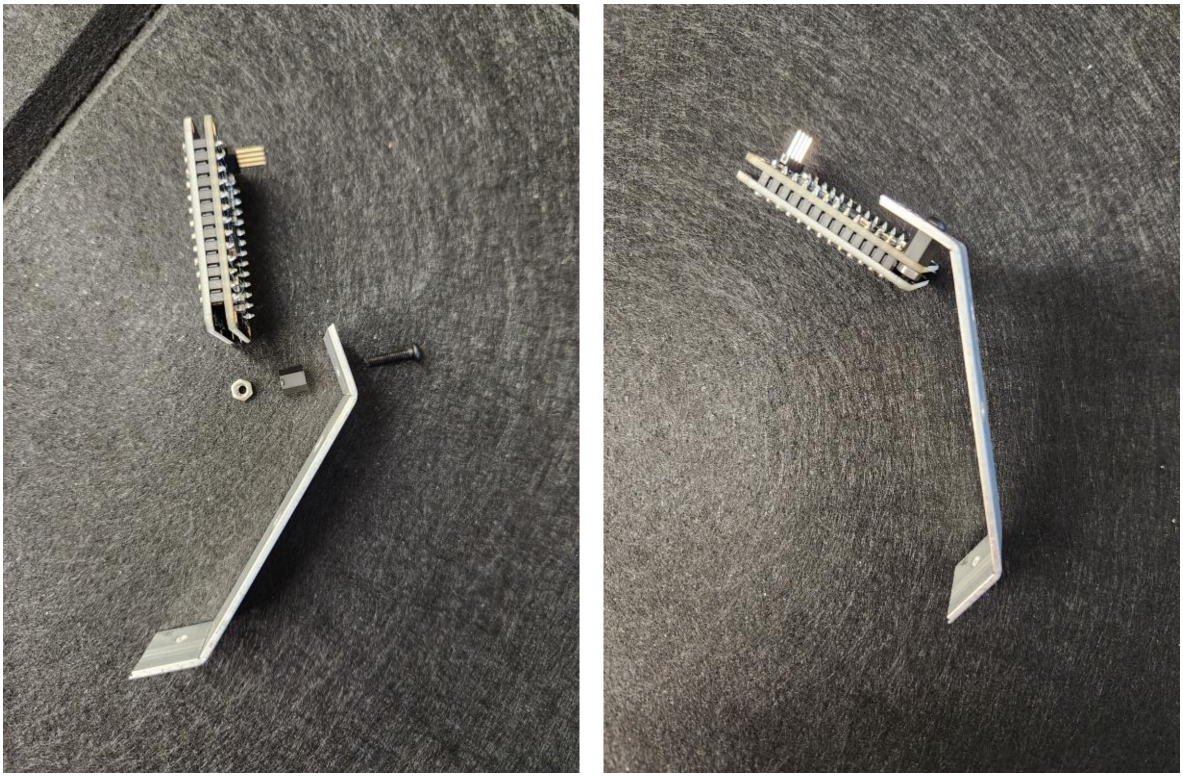

4. Attach the panels to the aluminum or printed plastic holder

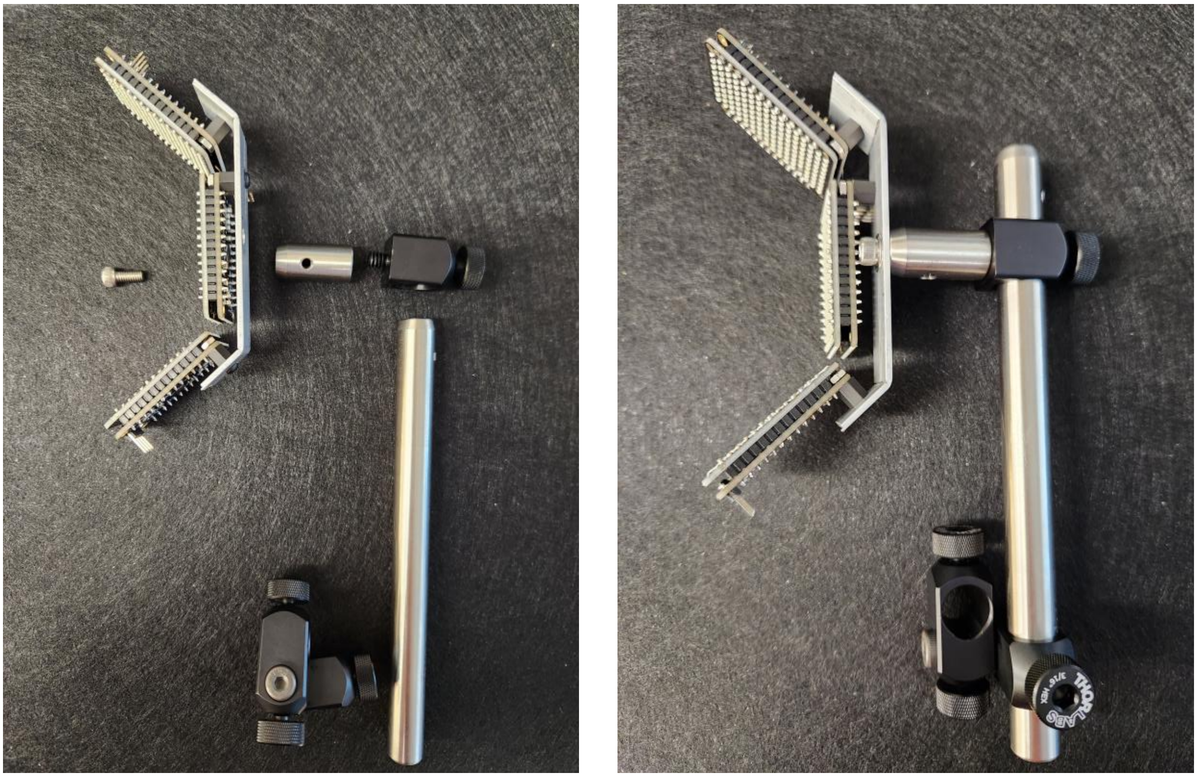

5. Attach the holder to the optomechanic posts with an 8-32 screw

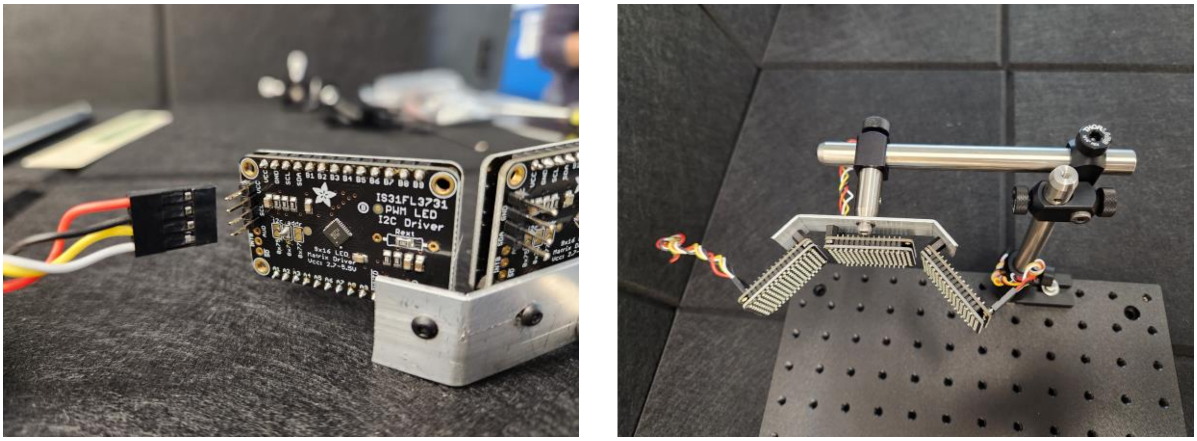

6. Plug in an extension cord (standard jumper wires will work but a 4x pin female to female jumper is more convenient to use

Auditory Stimulus – Speakers

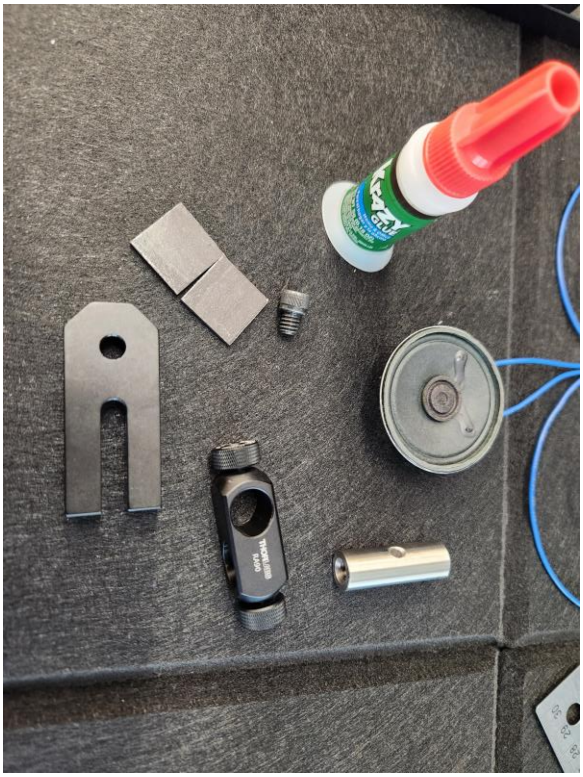

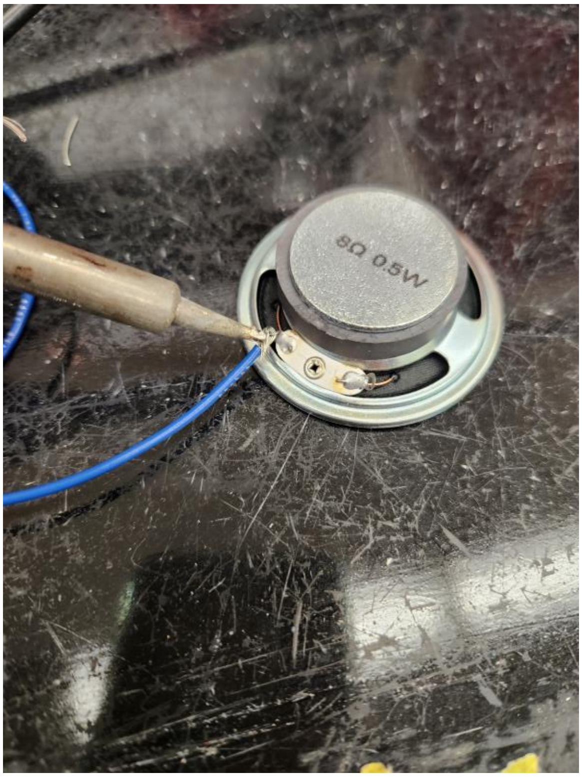

1. Parts: self adhesive magnet squares (any other magnet will work), speakers, wires, post assembly. Solder the wire to the input of the speakers.

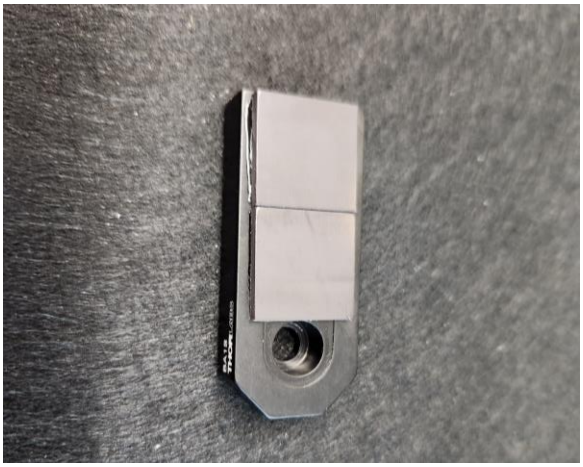

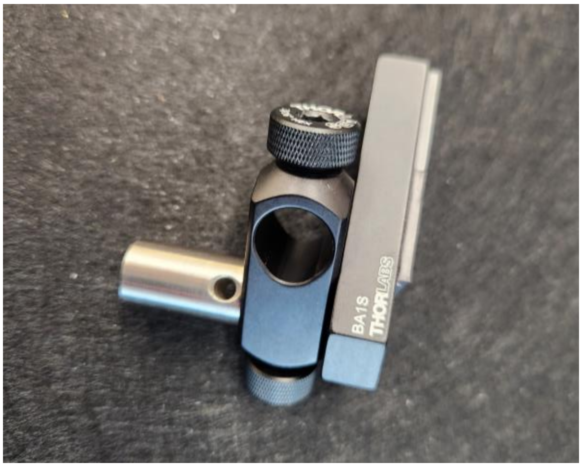

2. Attach the magnets to a post base and assemble the holder

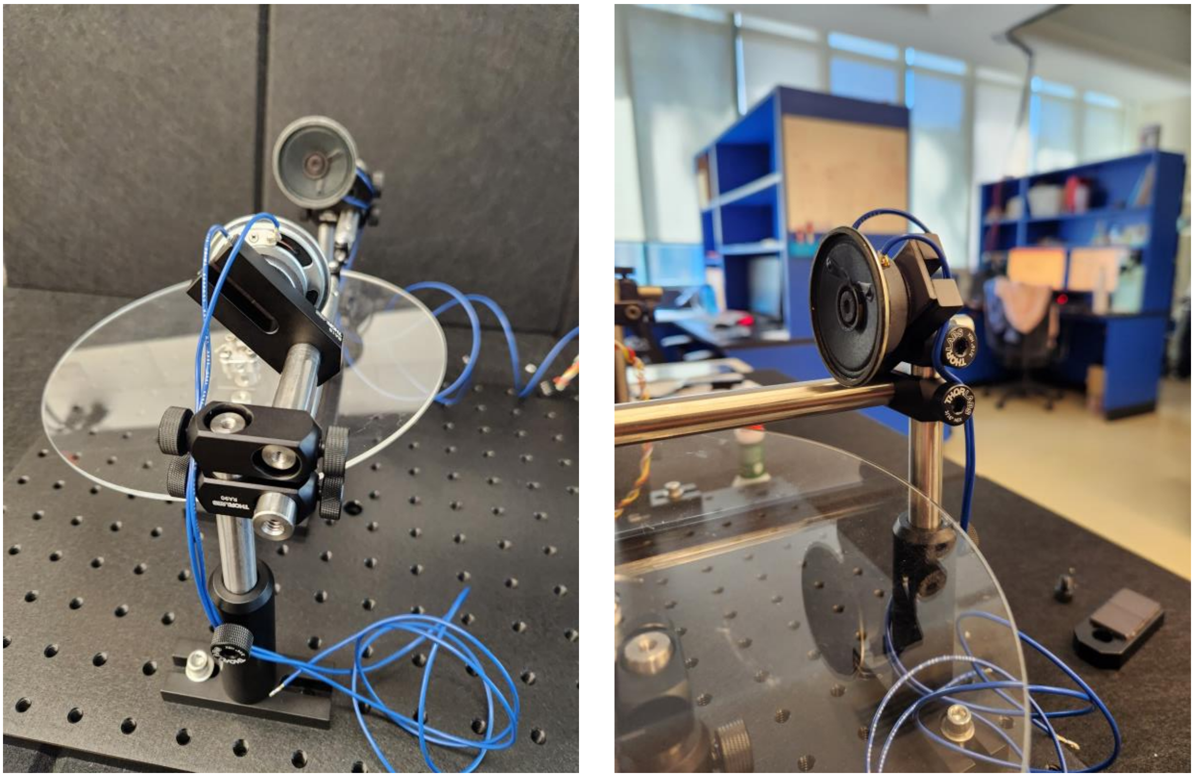

3. Add the speaker holder to the headplate holder post

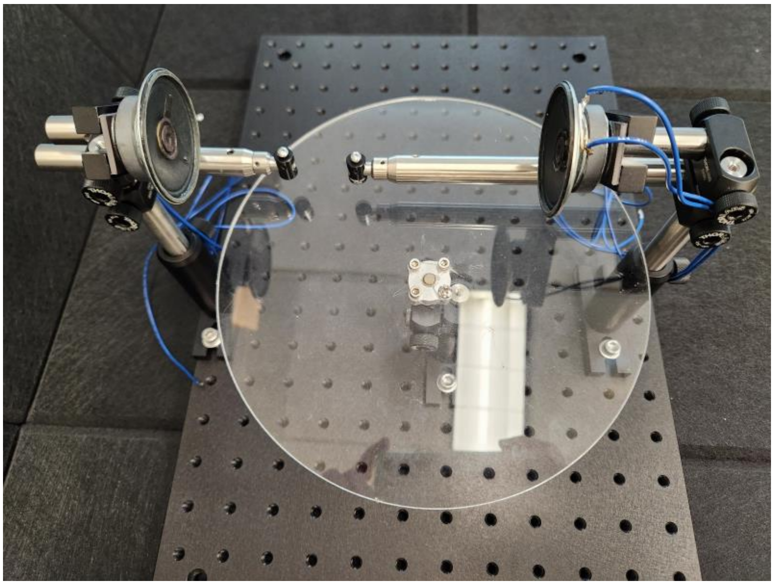

4. Adjust the distance so the two speakers are equidistant from the animal’s head (right side will need longer post than pictured)

Moving spout assembly

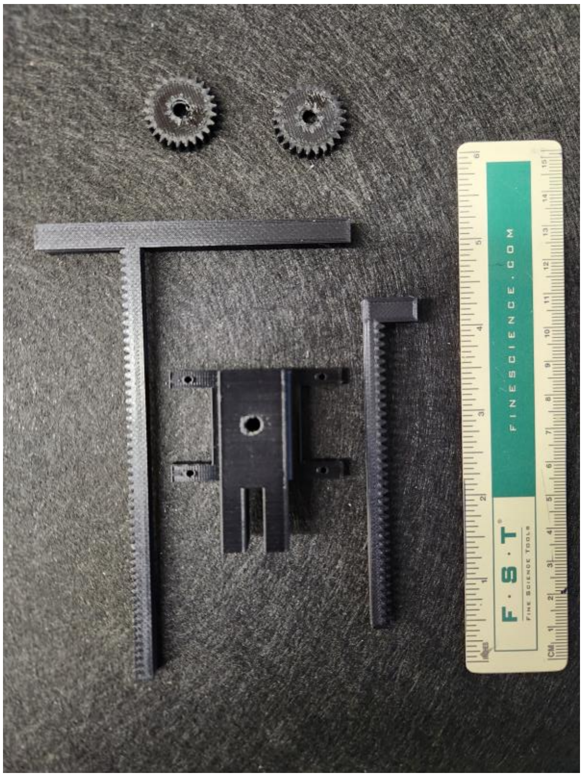

1. 3D print the parts for the servo to linear motion translator. This solution is recommended over the linear actuators if there is access to a 3D printer because the servos are much easier to replace than the linear actuators. Servos and actuator will both break down during use (typical lifetime is 3-6 months of daily use).

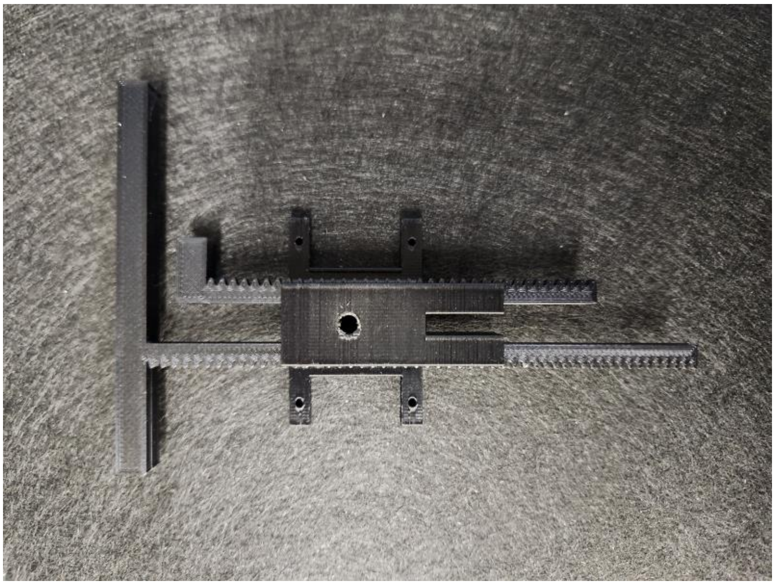

2. Assemble the printed parts and make sure it moves with ease

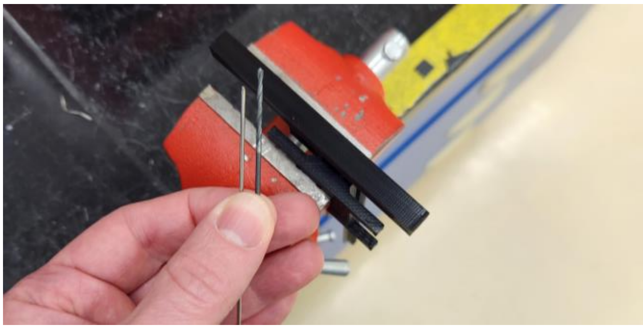

3. A 1/16 bit will be appropriate for an 18G lick spout tube

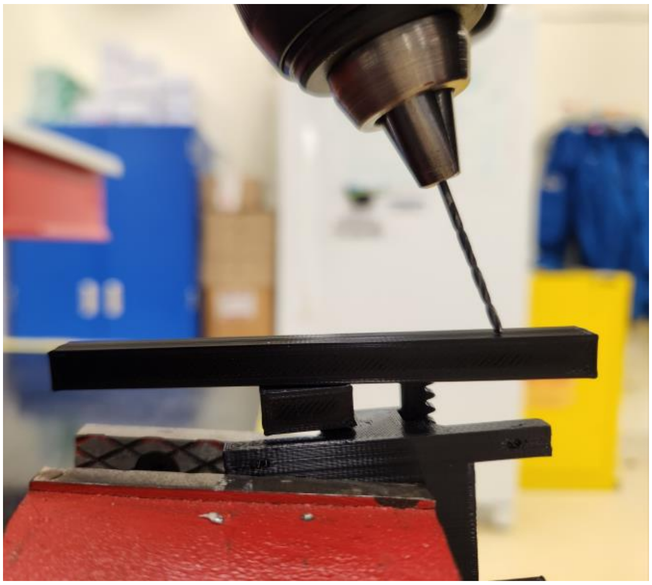

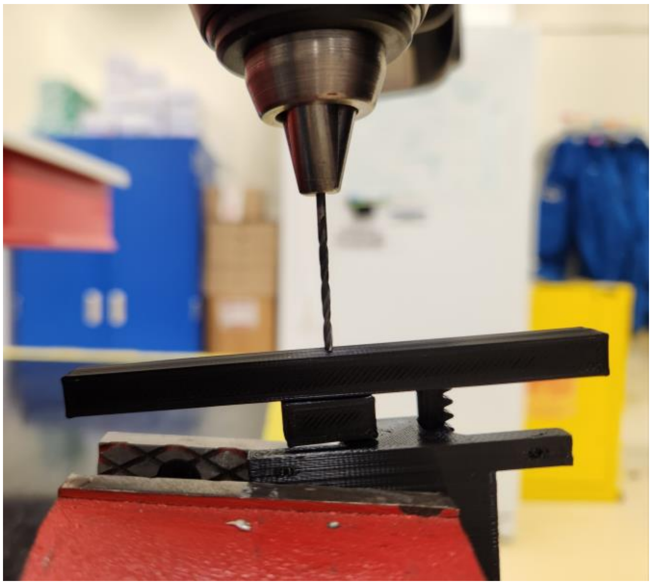

4. Drill the holes. Side spout holes are approximately at 30 degrees. Drill slightly larger holes than necessary to allow fine adjustments.

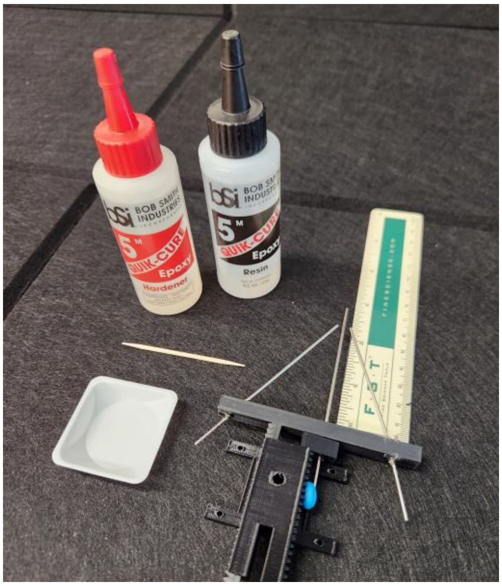

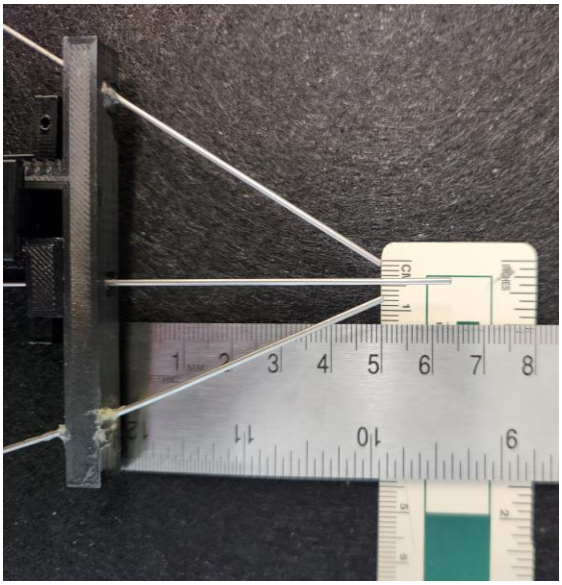

5. Use 5-minute epoxy to secure the spouts to the plastic parts

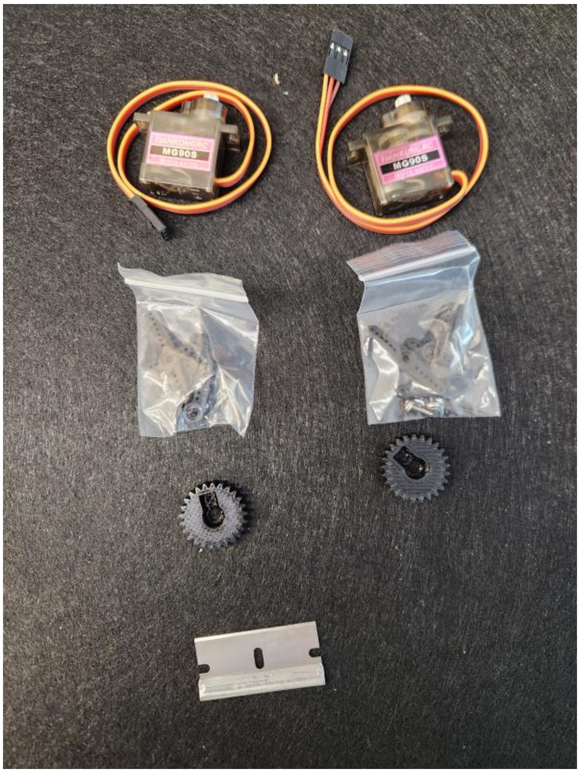

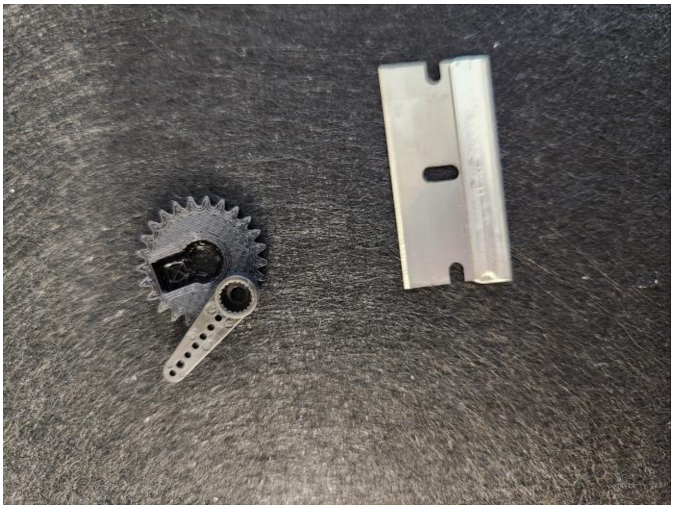

6. The MG90S servos come with standard attachments, one of these needs to be cut to fit in the slot on the printed gears.

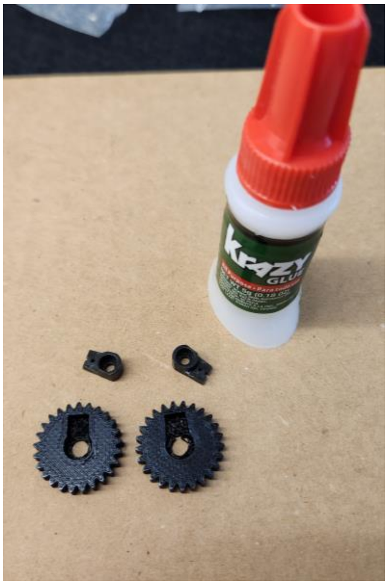

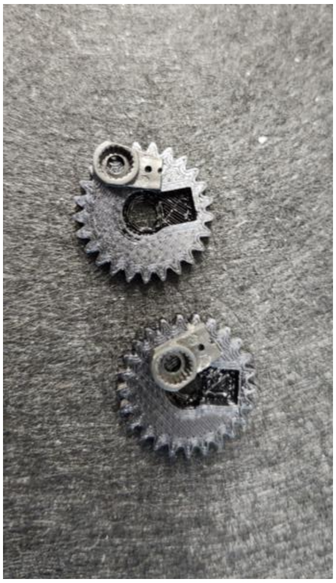

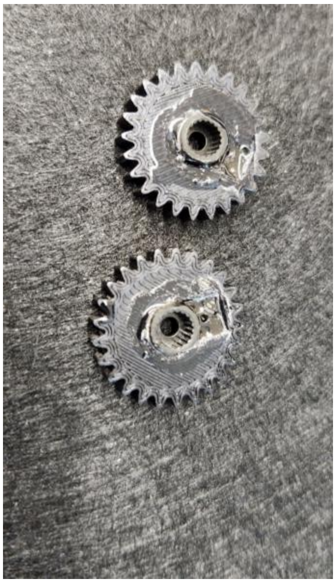

7. Use 5-minute epoxy or superglue to permanently insert the servo attachment piece to the printed gear.

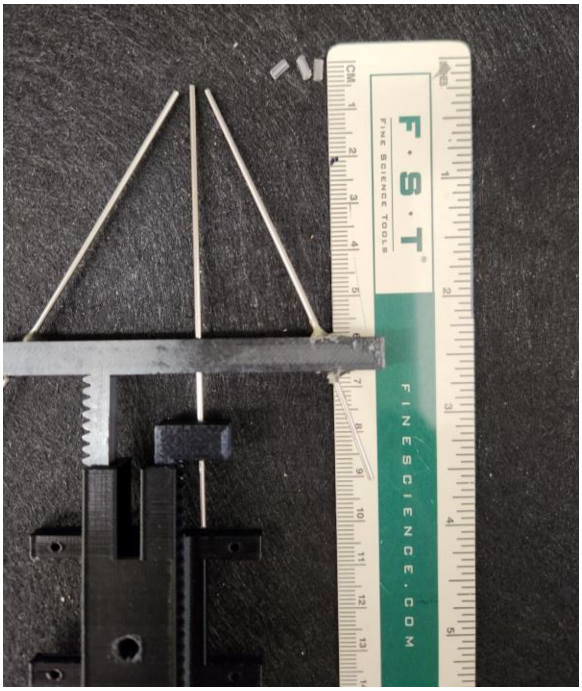

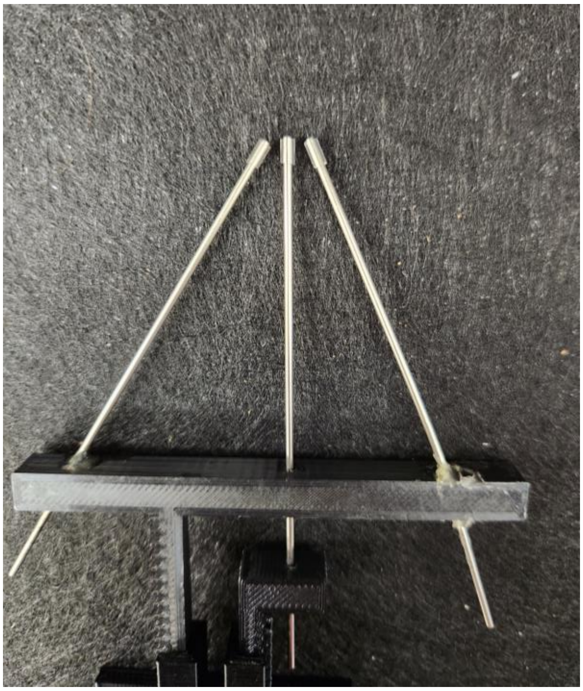

8. Cut three small (2-3mm) pieces of Tygon tubing (1/25 ID fits the 18G stainless tube well) and pull it over the lick spout tubes. This will support the formation of small droplets at the end of the spout.

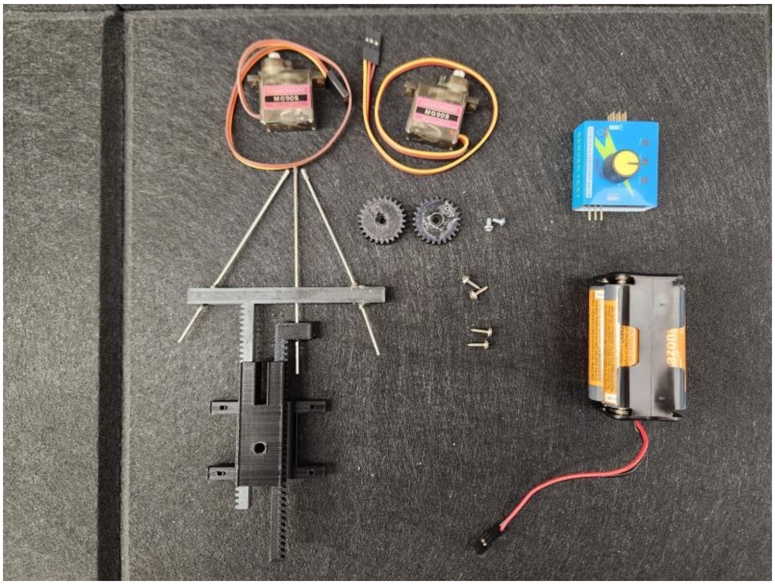

9. Parts needed to mount servos to the spout assembly

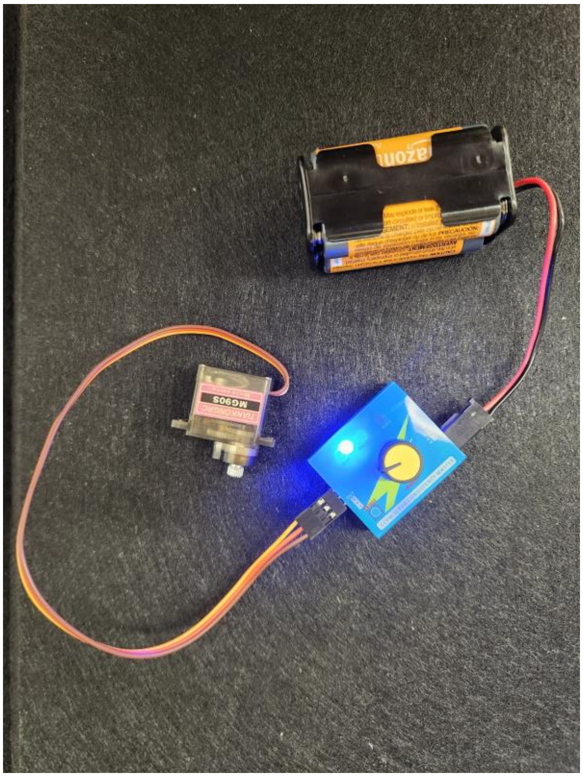

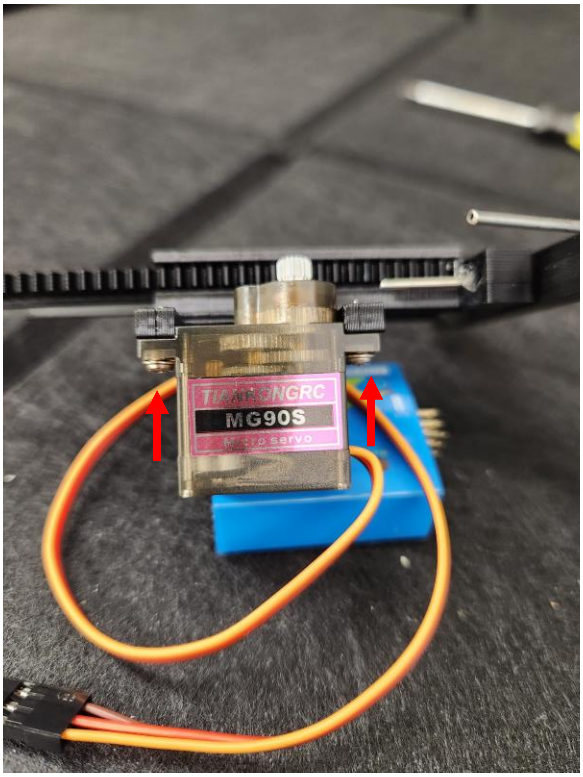

10. Use a manual servo controller to set servos to their end point and attach them to the spout assembly using the screws supplied with the MG90S (red arrows).

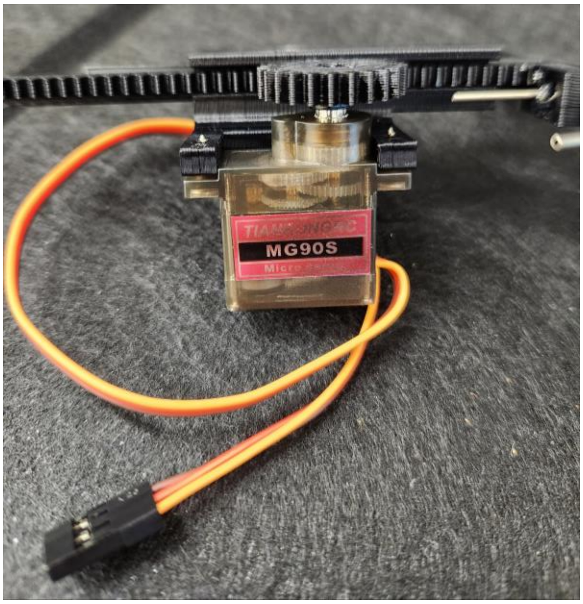

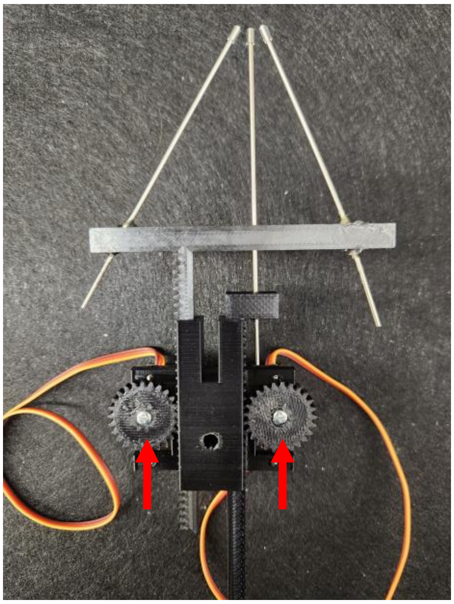

11. Set the spout holder arms to their full retract position and add the gears to the servo, secure the grear with the screw supplied with the servo

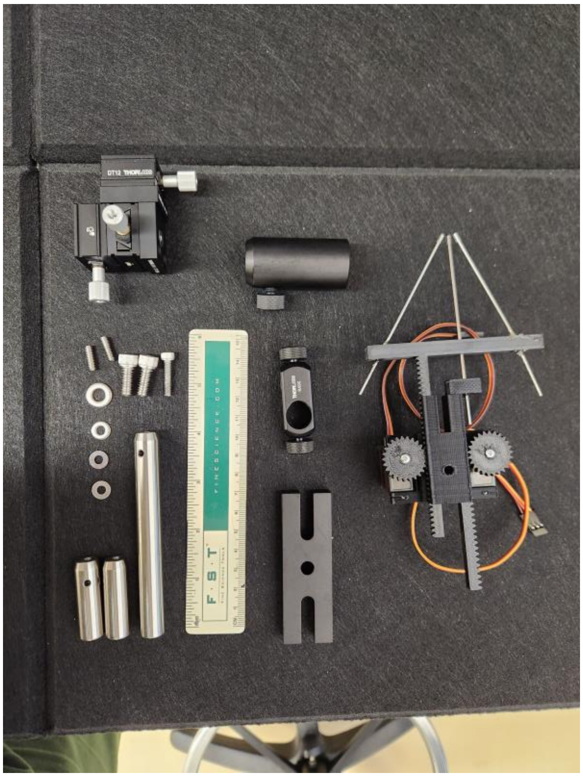

12. Parts for attaching the spout assembly to the 3D stage and post.

Thorlabs 3D stage DT12

Basic optomechanic components

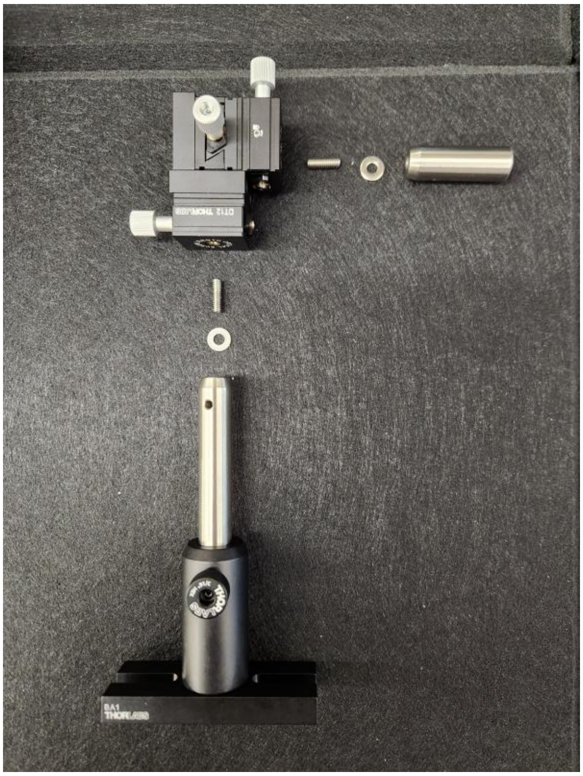

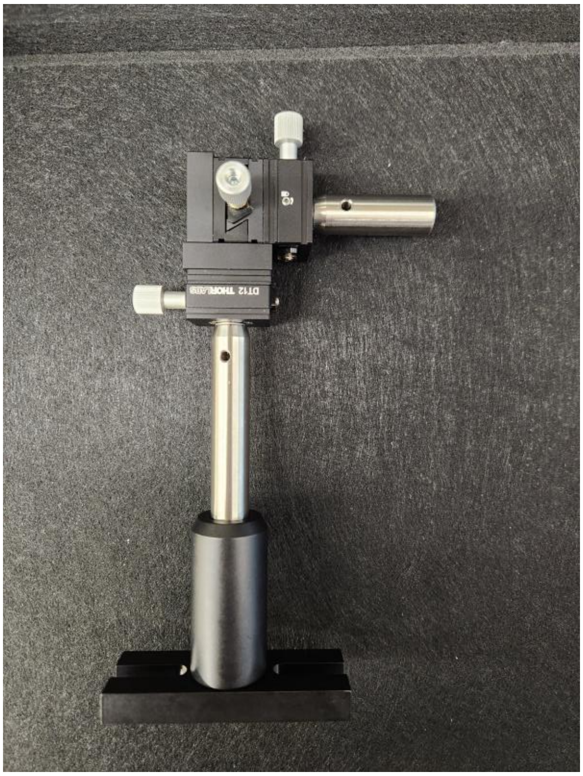

13. Assemble the 3D stage with the posts

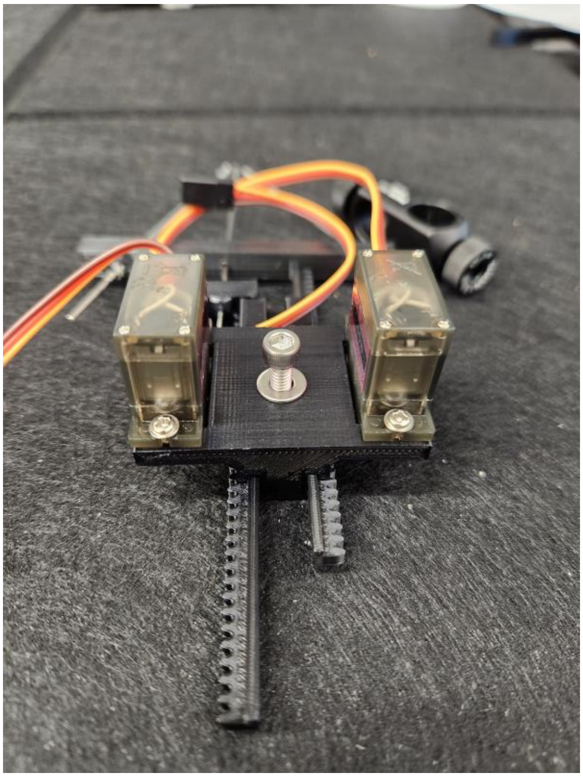

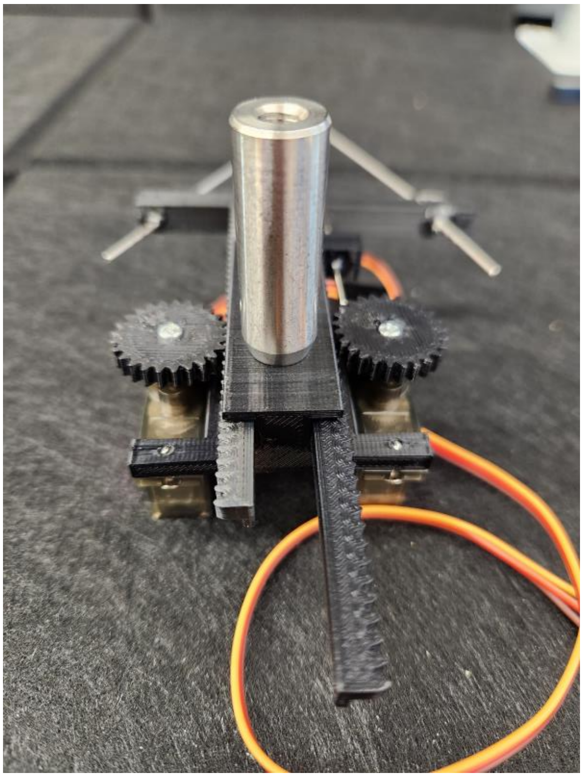

13. Secure a short (1.5-2 inch) post to the spout assembly using an 8-32 screw

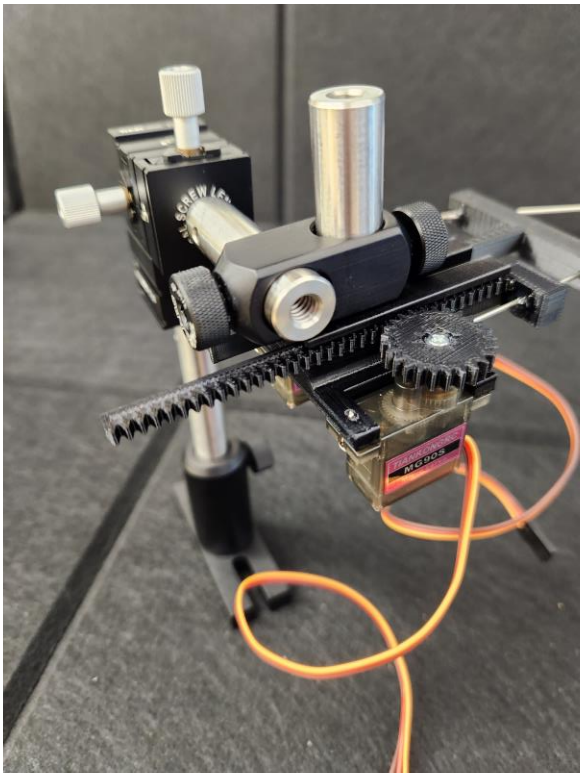

14. Add the spout assembly to the 3D stage with a right angle.

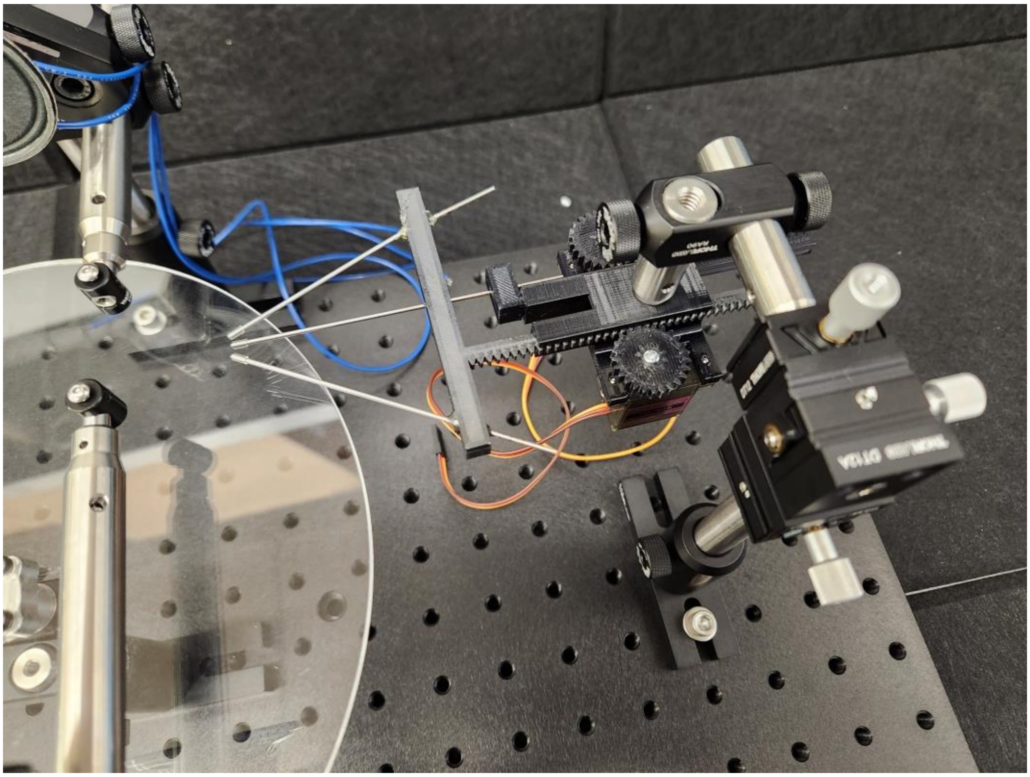

15. Mount the full spout assembly on the breadboard at a distance where the spouts can reach the animal's snout.

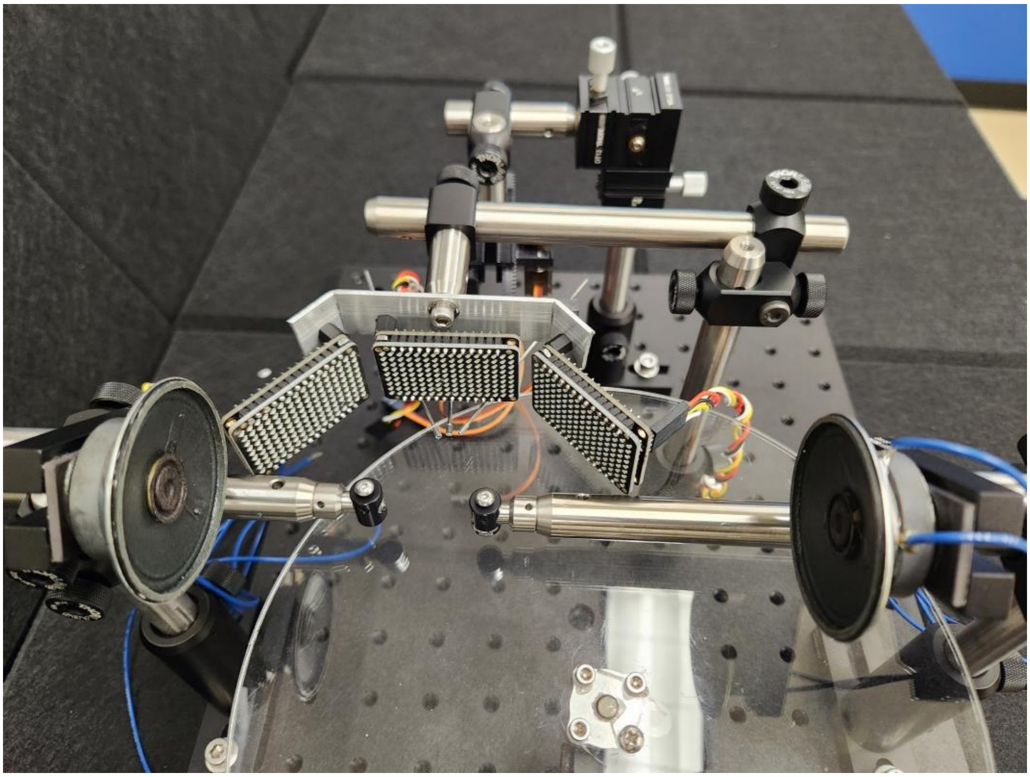

16. Position the LED crescent above the spouts. Make sure the center spout is centered under the center panel

Solenoid Valves

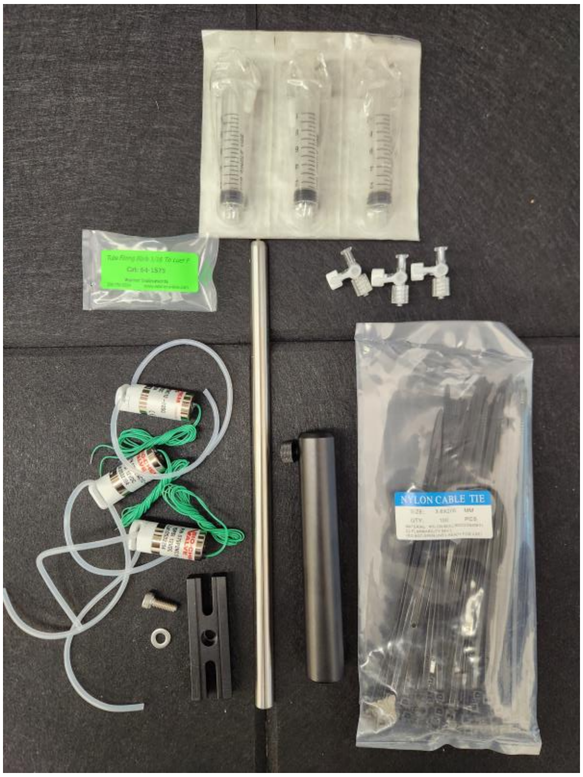

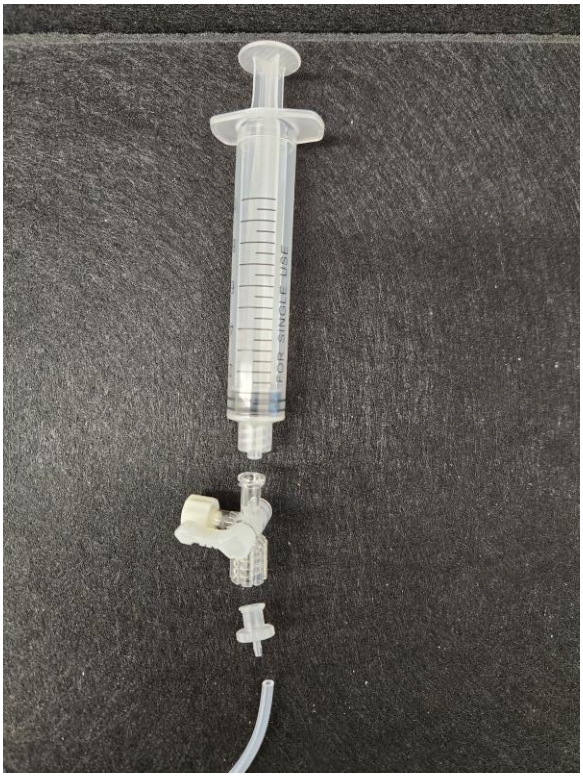

1. Parts for mounting reward valves (2-way stopcock is better). Add stopcock and barbed (1/16 inch) Luer lock connection.

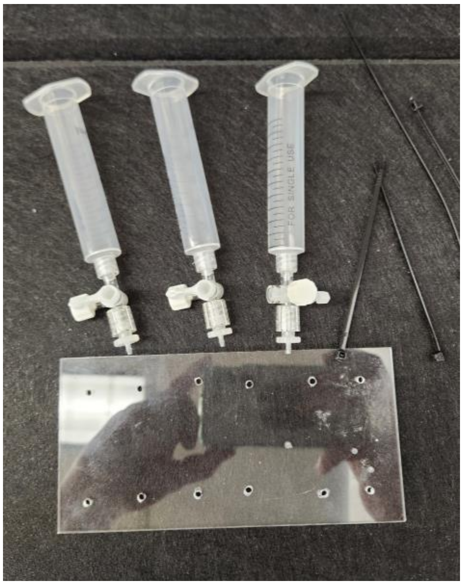

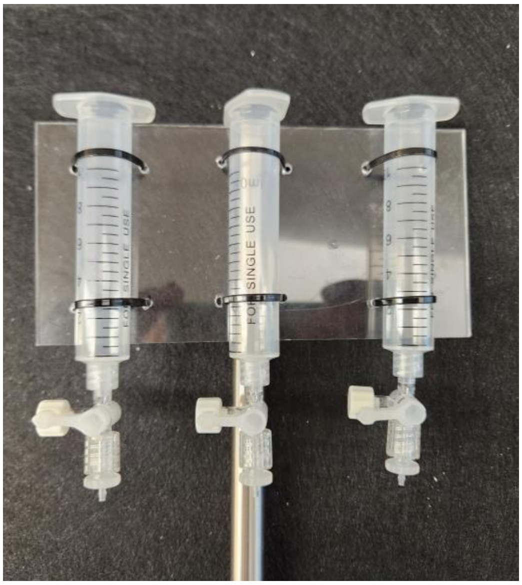

2. Mount the syringes to a piece of acrylic with zip-ties (piece of cardboard works just as well), and mount on a post.

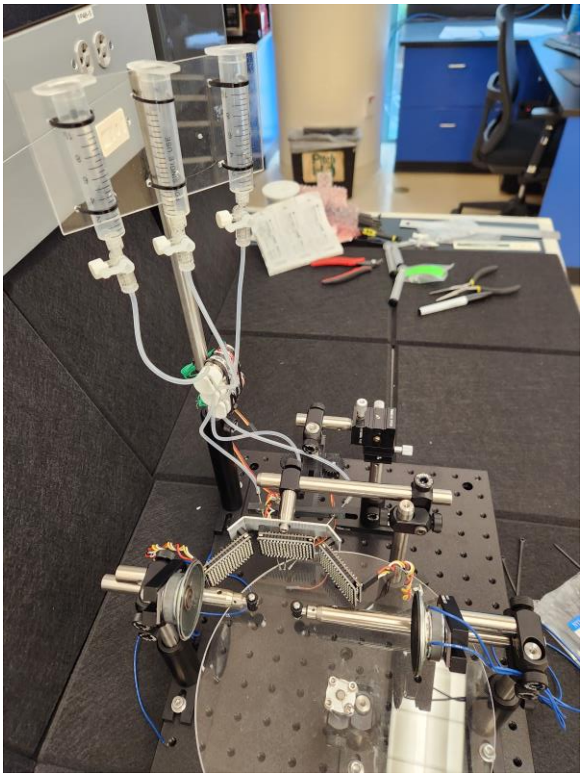

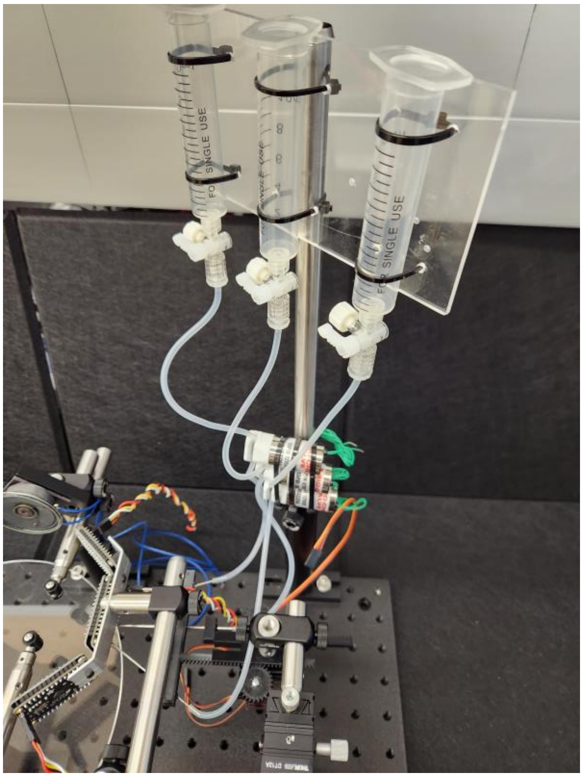

3. For demonstration purposes we mounted the water reservoirs (syringes) and valves on the breadboard. A better position for these is outside of the sound isolation cubicle. Mounting hardware and solutions may vary by application and environment.

Cable management

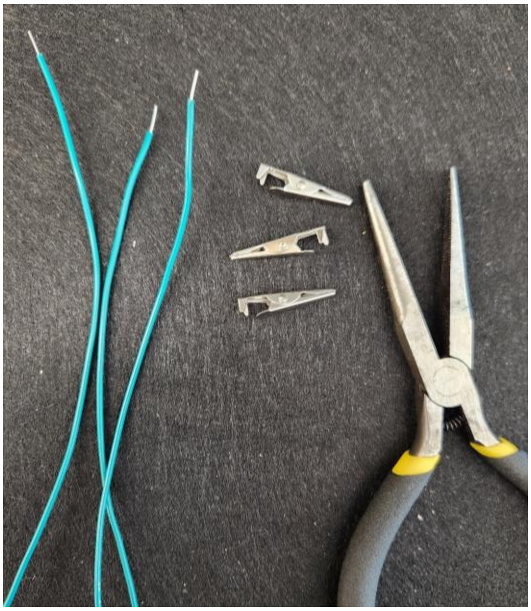
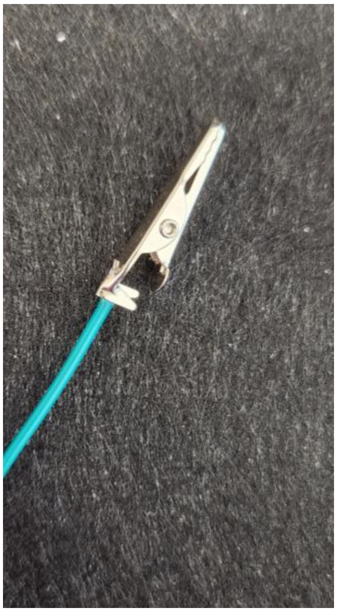

1. Cut the wires for the lick detection to the appropriate length, strip both ends and attach a metal alligator clip to one end of each wire

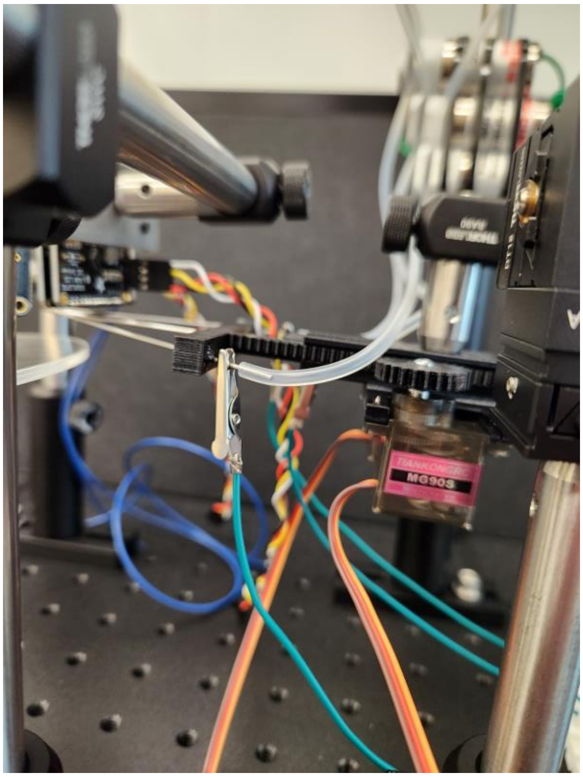

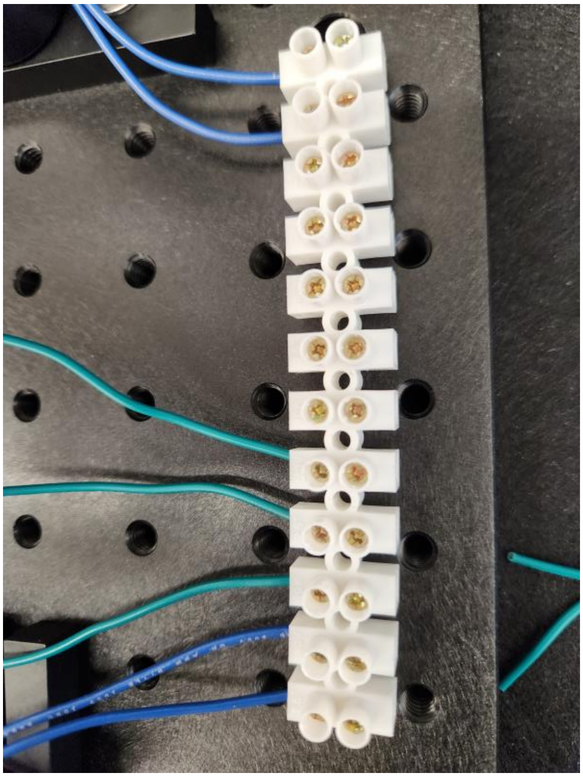

2. Attach the alligator clips to the lick spouts and secure the other ends to the screw terminal block

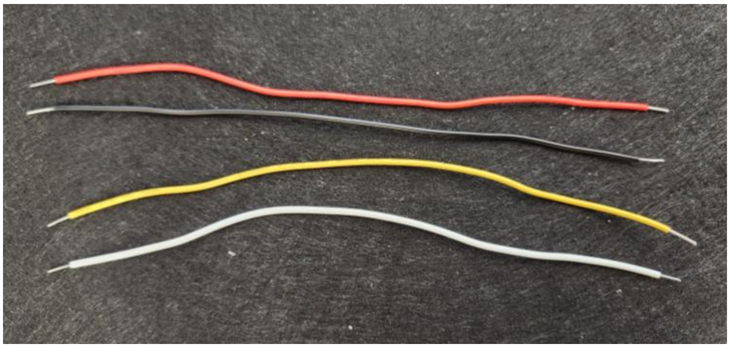

3. Cut three sets of wires of four different colors to the appropriate length to reach the terminal block, strip both ends.

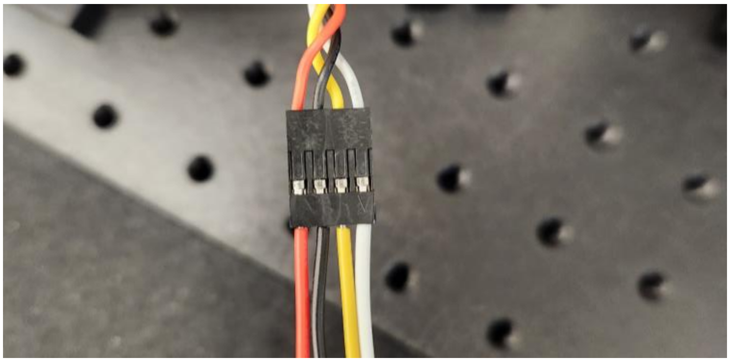

4. Connect the wires to the jumper cable going to the LED panels. 20G wires will give a secure fit

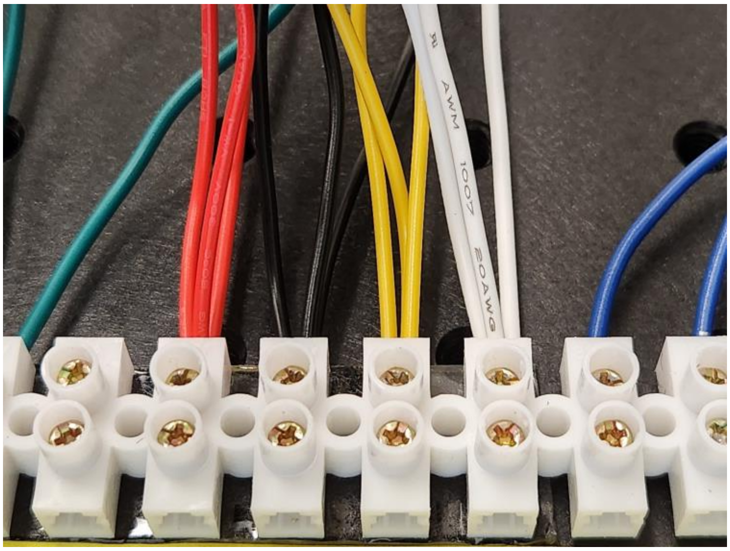

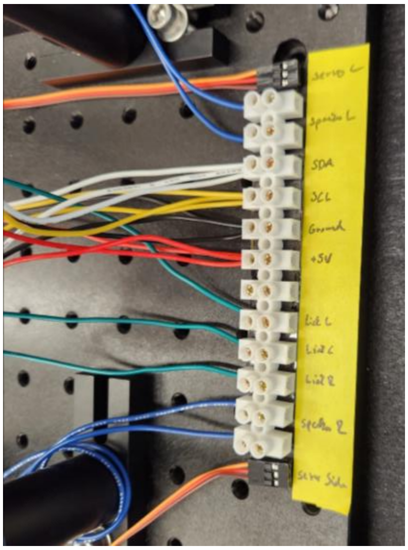

5. Secure the wires in the screw terminal. You can fit three wires to the same termination point making cable management easier.

Your mechanical parts are ready to connect to the electronics.

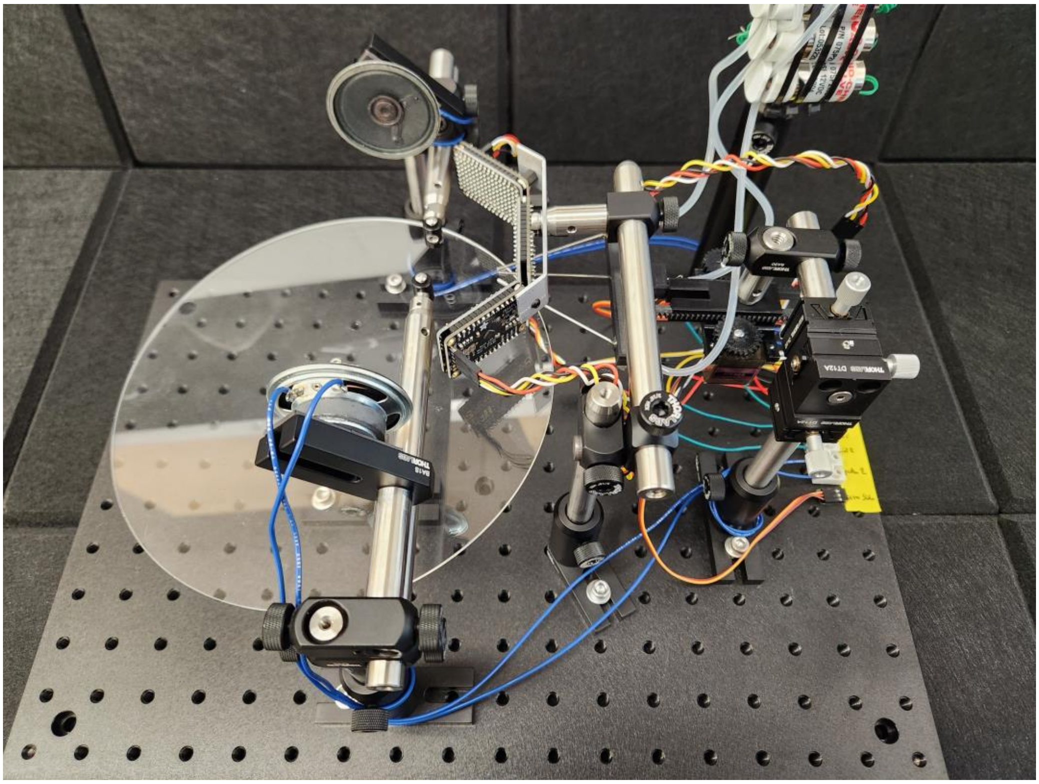

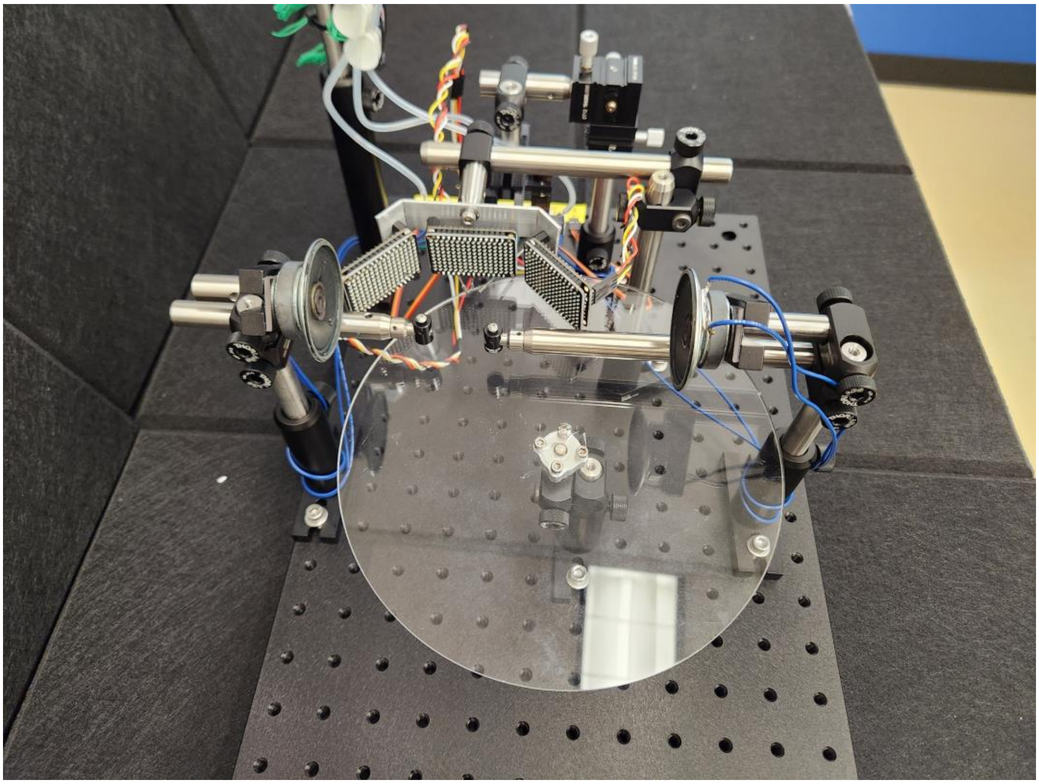

Supplement: Extended Data 1 — Extended Data containing 3D files, all necessary code, example video clips, parts list, detailed build instructions and full software documentation. Download Extended Data 1, ZIP file. [file enu-eN-MNT-0018-23-s02.zip › HERBs mechanical hardware build instructions.pdf]
