# Supplementary material for: A Comprehensive, Affordable, Open-Source Hardware-Software Solution for Flexible Implementation of Complex Behaviors in Head-Fixed Mice
Source: eNeuro. 2023 Jun 26;10(6):ENEURO.0018-23.2023. doi: 10.1523/ENEURO.0018-23.2023 (PMC10306125; doi:10.1523/ENEURO.0018-23.2023)
Supplement: Extended Data 1 — Extended Data containing 3D files, all necessary code, example video clips, parts list, detailed build instructions and full software documentation. Download Extended Data 1, ZIP file. [file enu-eN-MNT-0018-23-s02.zip › Software Documentation.pdf]

A comprehensive, affordable, open-source hardware-software solution for flexible implementation of complex behaviors in head-fixed mice.

Software Documentation

1. MATLAB version and dependencies

MATLAB 9.12 (2022a)
Dependencies:

- Instrument Control toolbox for serial communication

To run the software without having to install MATLAB, first install the MATLAB Runtime library for version (R2022a (9.12)) freely available here: https://www.mathworks.com/products/compiler/matlab-runtime.html. We also included an installer for the Runtime library in our package (Executables/Runtime_installer), launching this application will install the necessary libraries in the Program Files of the computer. After installing Runtime, the executable file corresponding to the desired experimental framework can be run to launch the GUI (Executables/exe_files). Launching the GUIs in this manner will take somewhat longer but the result is identical to using MATLAB.

2. Arduino MEGA libraries

- MsTimer2.h
- TimerThree.h
- Wire.h
- Adafruit_BusIO
- Adafruit_GFX.h
- Adafruit_IS31FL3731.h
- Adafruit_MCP4728.h
- Adafruit_MCP4725.h
- Servo.h
- Servo Hardware PWM

3. Arduino DUE libraries

- Wire.h
- Tone.h

4. Example MATLAB to Arduino MEGA communication

To instruct the Arduino Mega to open Valve C use the following command:

```
write(arduino,['k' 1 1] "uint8");
```

This writes 3 bytes to the Arduino serial port:

‘k’ to indicate that this is a valve operation
1 to indicate that it is the Center valve
1 to indicate that the valve is to be opened

The Arduino first receives the ‘k’ and decodes it and reads two more bytes (temp_byte and temp_byte1). temp_byte determines which of the three valves is to be operated on, then temp_byte1 determines whether the valve is to be opened or closed.

When Arduino Mega receives serial data from Matlab it decodes the first byte of the data as follows. Depending on the byte it proceeds with further reads and processing.

```
// valve control
            if (inChar == 'k')
            {
              while (!Serial.available()) {}
              temp_byte = Serial.read();
              while (!Serial.available()) {}
              temp_byte1 = Serial.read();

              if (temp_byte == 0)   // 0 is left valve
              {
                if (temp_byte1 == 1)
                  digitalWrite(RewardL, HIGH);
                  else
                  digitalWrite(RewardL, LOW);
              }
              else if (temp_byte == 1)   // 1 is center valve
              {
                if (temp_byte1 == 1)
                  digitalWrite(RewardC, HIGH);
                  else
                  digitalWrite(RewardC, LOW);
              }
              else     // 2 is right valve
              {
                if (temp_byte1 == 1)
                  digitalWrite(RewardR, HIGH);
                  else
                  digitalWrite(RewardR, LOW);
              }
              return;
            }
```

5. Arduino LED panel operation

The system uses three Adafruit_IS31FL3731 LED light panels. These panels contain 16 rows and 9 columns of LED’s. Each LED can be turned on or off individually. The panels contain 8 pages of memory, i.e. 8 grids of patterns can be saved and controlled as pages of patterns.

Here is one example of how to initialize the left LED panel for use.

```
////left panel
  write_panel_pageL(6,9,ystart,ystop,0,light_pattern_brightness);
  write_panel_pageL(12,15,ystart,ystop,1,light_pattern_brightness);
  write_panel_pageL(0,15,0,ystop,2,0);
  write_panel_pageL(5,9,3,6,pg_small,0); // small panel
```

```
  write_panel_pageL(0,15,0,ystop,pg_full,light_pattern_brightness);
  write_panel_pageL(0,3,ystart,ystop,5,light_pattern_brightness);
  write_panel_pageL(0,15,0,ystop,6,0); // small panel
  write_panel_pageL(0,15,0,ystop,pg_dark,0); //dark
```

Where:
light_pattern_brightness contains the brightness value
pg_small = 3, the page number for a small pattern
pg_full = 4, the page number for a full pattern (all turned on)
pg_dark = 7, the page number for the dark page (all turned off)
ystart = 0, the start y column for filling the patterns
ystop = 8, the stop y column for filling the patterns

this code creates the following 8 pages for the panel:
page0 pixels inside columns 6-9 and rows from 0-8 to brightness value
page1 pixels inside columns 12-15 and rows from 0-8 to brightness value
page2 pixels inside columns 0-15 and rows from 0-8 to 0 (all pixels off)
page3 pixels inside columns 5-9 and rows from 3-6 to brightness value
page4 pixels inside columns 0-15 and rows from 0-8 to brightness value (all pixels on)
page5 pixels inside columns 0-3 and rows from 0-8 to brightness value
page7 pixels inside columns 12-15 and rows from 0-8 to 0 (all pixels off)
page8 pixels inside columns 12-15 and rows from 0-8 to 0 (all pixels off)

Once the panels have been initialized, they can easily be controlled with simple commands
For example:

ledmatrixL.displayFrame(pg_dark);	turn off all pixels in the left panel
ledmatrixR.displayFrame(pg_full);	turn on all pixels in the right panel
ledmatrixL.displayFrame(pg_small);	turn on pixels (5-9,3-6) in the left panel

6. Auditory signal generation with Arduino DUE

The sine generation uses a 32-point lookup table created by the following code Arduino code:

```
SAMPLES = 32;
CEILING = 4096

void CreateSineWaveTable0() {
 for(int i = 0; i < SAMPLES; i++)
  {
       tfl =  CEILING/2 +
(CEILING/2)*sin(float(2*3.1416)*float(i+1)/SAMPLES);
       temp = round(tfl);
       if (temp>(CEILING-1))
          temp = CEILING-1;
      wav0[i] = temp;
  }
}
```

Two tables are generated, one for DAC0 and one for DAC1.
To produce a sinewave all the samples are sent to DAC0 or DAC1 at a certain rate (at about 64 kHz to produce 2 kHz tone and about 1 MHz to produce 32 kHz tone). that determines the final output frequency of the signal.

The table in graph form:

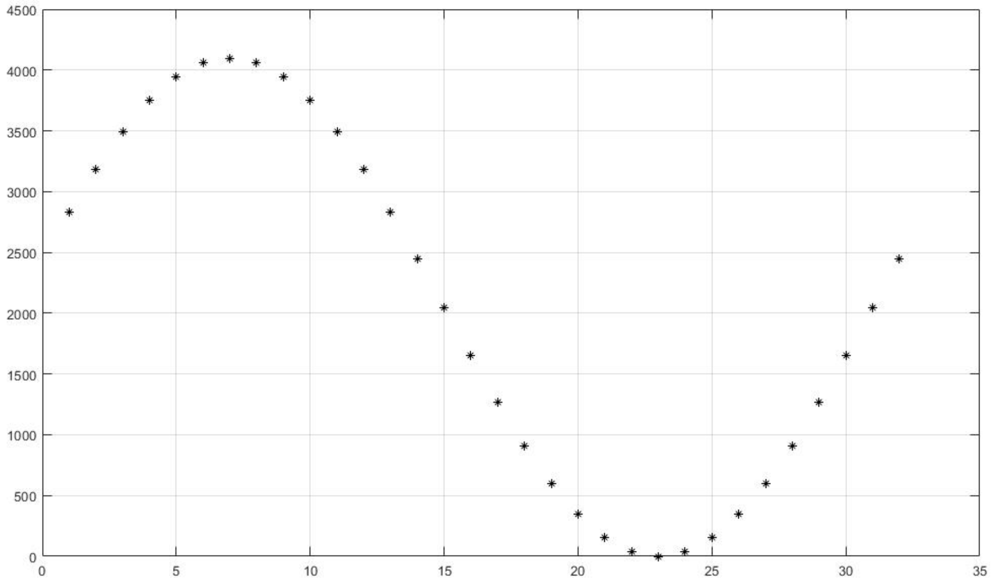

The samples are not explicitly written to the DAC but are transferred via DMA (Direct Memory Access). The DMA cycle (32 samples in this case) repeats and continues until it is stopped by a command.
Noise is produced in a similar fashion. The signal table is now larger, 8192 samples of random values and the sampling rate is set to 3 kHz. The noise signal repeats after every 8192 samples and as a result is periodic.

7. Arduino MEGA to DUE communication

Communication follows the logic below:

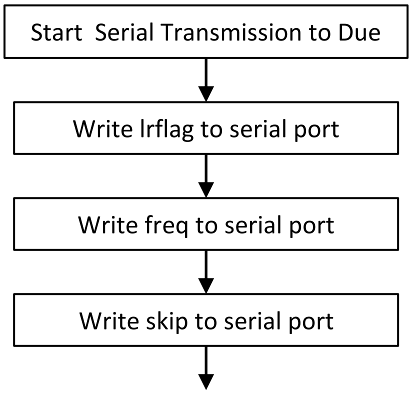

This is enacted by the following code in the Arduino MEGA:

```
Wire.beginTransmission(9); // transmit to device #9
Wire.write(lrflag);              // sends x
Wire.write(freq);              // sends y
Wire.write(skip);              // sends skip
Wire.endTransmission();    // stop transmitting
```

When serial transmission from MEGA is detected, the DUE follows the below logic:

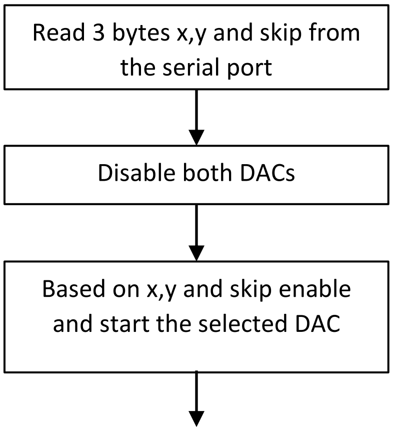

Using the following code:

```
x = Wire.read();        // read one byte
y = Wire.read();        // read one bye
skip = Wire.read();     // read one bye

If y = 2 and x = 1  noise on DAC0
If y = 2 and x != 1  noise on DAC1
If y != 2  and skip = 1  and x = 1    2kHz   on DAC0
If y != 2  and skip = 1  and x = 2    2kHz   on DAC1
If y != 2  and skip = 2  and x = 1    4kHz   on DAC0
If y != 2  and skip = 2  and x = 2    4kHz   on DAC1
If y != 2  and skip = 3  and x = 1    6kHz   on DAC0
If y != 2  and skip = 3  and x = 2    6kHz   on DAC1
If y != 2  and skip = 4  and x = 1    8kHz   on DAC0
If y != 2  and skip = 4  and x = 2    8kHz   on DAC1
If y != 2  and skip = 6  and x = 1    12kHz   on DAC0
If y != 2  and skip = 6  and x = 2    12kHz   on DAC1
If y != 2  and skip = 8  and x = 1    16kHz   on DAC0
If y != 2  and skip = 8  and x = 2    416Hz   on DAC1
If y != 2  and skip = 12  and x = 1    24kHz   on DAC0
If y != 2  and skip = 12  and x = 2    24kHz   on DAC1
If y != 2  and skip = 16  and x = 1    32kHz   on DAC0
If y != 2  and skip = 16  and x = 2    32kHz   on DAC1
```

8. Two-alternative forced choice (2AFC) GUI documentation

This GUI allows the user to customize stimuli, delays and task strategy, as well as check on a mouse's progress in real-time during a 2AFC task.

Control interface:

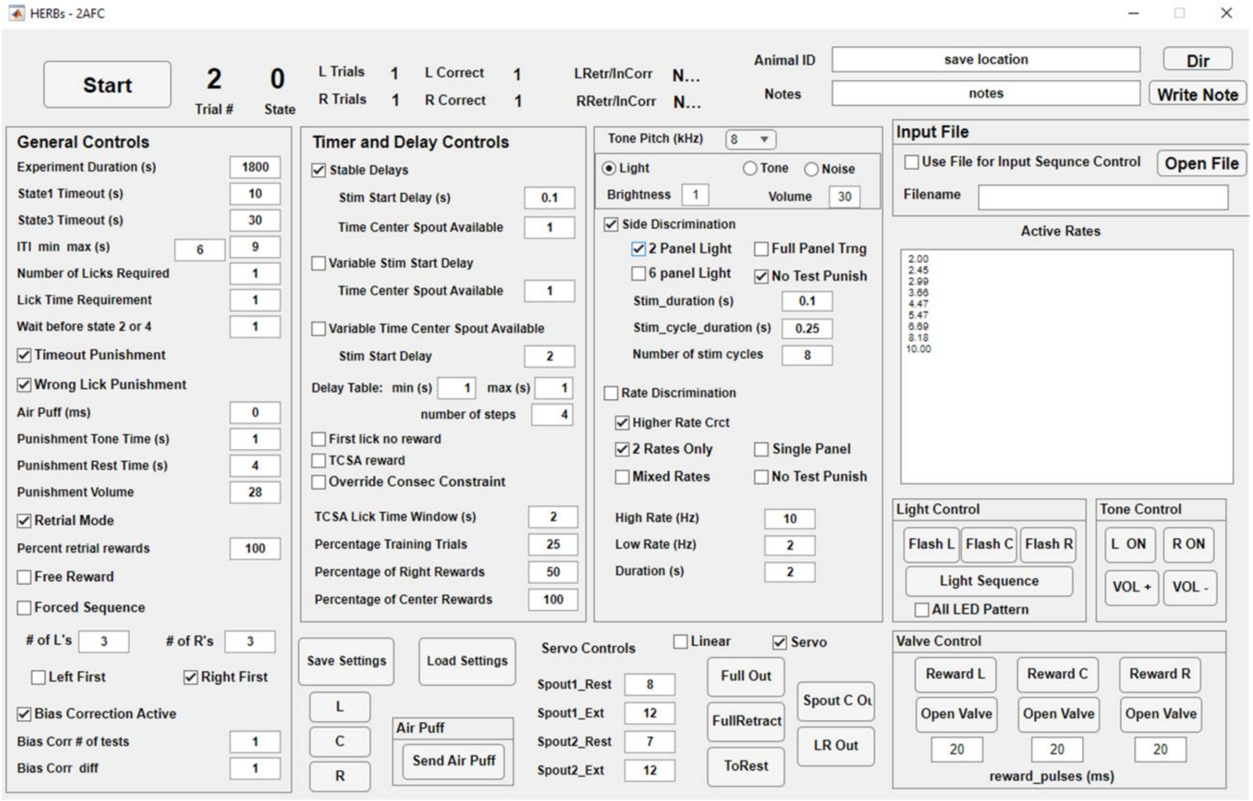

<u>Top panel:</u>

1. ***Start***: starts running the behavior
2. ***Trial #***: number of trials completed
3. ***State***: progress in the state machine (useful for troubleshooting)
4. ***L Trials***: total number of left trials completed
5. ***R Trials***: total number of right trials completed
6. ***L Correct***: number of correct left trials
7. ***R Correct***: number of correct right trials
8. ***LRetr/InCorr***: number of retrials / incorrect trials on the left
9. ***RRetr/InCorr***: number of retrials / incorrect trials on the right
10. ***Animal ID***: this can be a directory path to a saved data folder:
    a. Example: Z:\User\GNG\mouse1
11. ***Dir***: this allows the user to select the directory path to a saved data folder, and is used to prefill Animal ID (Item 2)
12. ***Notes*** bar: type in notes during the experiment
13. ***Write Note***: click to save timestamp for note, printed at the end of the experiment

Left Panel: General Controls

1. ***Experiment Duration(s)***: User inputs the length of the behavioral session in seconds.
    a. Example: A 5-minute session is 600s, so '600' would be typed.
2. ***State1 Timeout (s):*** The GUI stops the behavioral session if the mouse does not respond (ie, does not perform a lick) on the center spout to initiate the next trial.
3. ***State3 Timeout (s)***: The GUI stops the behavioral session if the mouse does not respond (ie, does not perform a lick) on one of the side spouts to make a decision.
4. ***ITI min max(s)***: the Inter Trial Interval length (in seconds), randomly selected in the given range
5. ***Number of Licks Required:*** the number of licks required to be counted as a correct response.
6. ***Lick Time Requirement:*** the amount of time (s) the lick requirement needs to be completed
7. ***Wait before state 2 and 4***: time allowed for the mouse to collect the reward before spout moves out of the way.
8. ***Timeout punishment***: check box if animal is to be punished for exceeding state time (given in #2 and 3).
9. ***Wrong lick punishment***: check box if animal is to get punishment for incorrect choice.
10. ***Air Puff (ms)***: if air puff is given as punishment set the opening time for air valve (0 if no air puff punishment)
11. ***Punishment Tone time (s)***: Length of punishment sound.
12. ***Punishment Rest time (s)***: Length of punishment timeout (including tone time).
13. ***Punishment volume***: Volume of punishment sound.
14. ***Retrial mode***: Check if animal is allowed to try again with the same stimulus after an incorrect choice.
15. ***Percent retrial rewards***: Defines what proportion of retrials are rewarded.
16. ***Free Reward***: This dispenses a water reward during the stim presentation.
17. ***Forced sequence***: Check this box if defined (not random) sequence of left and right trials is requested, number of left and right trials are defined in the boxes below. Check boxes determine if left or right trials start the sequence. Useful for bias correction. Sequences can be further refined by uploading a sequence file on the right side of the GUI in "***Input File***" section.
18. ***Bias Correction Active***: Check the box for automated bias correction. This looks at a given number of trials (set below) and if a given proportion of them are biased it automatically offers the opposite side. Number of tested trials and required difference are defined in the boxes below.

Center-Left panel: Timer and Delay Controls

1. ***Stable delays***: Check if delays throughout the experiment are meant to be unchanging.
    a. ***Stim Start Delay (s)***: sets the delay between advancing the center spout into reach and the start of the stimulus
    b. ***Time Center Spout Available***: Sets how long the center spout remains in reach before refracting and switching over to the side spouts.
2. ***Variable Stim Start Delay***: Check for the delay between center spout available and stimulus presentation is to be variable. Delays are set in the ***Delay Table*** below

    a. ***Time Center Spout Available***: Sets how long the center spout remains in reach before refracting and switching over to the side spouts.
3. ***Variable Time Center Spout Available***: Check for the delay between stimulus presentation and center spout retraction is to be variable. Delays are set in the ***Delay Table*** below
    a. ***Stim Start Delay (s)***: sets the delay between advancing the center spout into reach and the start of the stimulus
4. ***Delay Table***: sets the durations for variable delays. Range is defined as ***min*** and ***max***, in seconds, steps are defined as ***number of steps***.
5. ***First lick no reward***: Suppresses reward on the first lick to Center spout.
6. ***TCSA reward***: check if want to give a reward for continued licking during the ***Time Center Spout Available*** duration.
7. ***Override Consec Constraints***: overrides logic for maximum number of trials on the same side in a row.
8. ***TCSA Lick Time Window***: defines the time window for receiving TCSA reward
9. ***Percentage Training Trials***: Defines the proportion of training (easy, simple choice) trials during the experiment. Typically 60-70% of all trials should be "training trials" to maintain motivation.
10. ***Percentage of Right rewards***: Defines how are the left and right trials split, moving away from 50% could aid bias correction, 66% or above for one side requires ***Override Consec Constraints*** to be checked.
11. ***Percentage of Center rewards***: Defines what proportion of trial initiations on central port are rewarded.

<u>Center-Right panel: stimulus controls</u>

1. ***Tone Pitch (kHz)***: defines the pitch of the stimulus in kHz if sound is used
2. ***Light / Tone / Noise***: radio buttons select the type of stimulus
3. ***Brightness***: defines the brightness of the LED panels if visual stimulus is used
4. ***Volume***: defines the volume of the auditory stimulus if sound is used
5. ***Side discrimination***: select if the task is based on discriminating between left and right stimulus location
    a. ***2 Panel Light***: only the two side panels are used
    b. ***Full Panel Trng***: if checked, the whole panel lights up, option for training
    c. ***6 Panel Light***: randomly shows stimuli in one of 6 locations on the LED crescent for psychometric testing, works well with visual stim, would require additional speakers for auditory (auditory not tested)
    d. ***No Test Punish***: when checked, suppresses punishment on light location that are not the training locations
    e. ***Stim_duration (s)***: length of the stimulus
    f. ***Stim_cycle_duration (s)***: duration of a single stim cycle, allows precise control between stim on and off times
    g. ***Number of stim cycles***: defines how many stim cycles are ran. If a lick is detected, stimulus stops.

6. ***Rate Discrimination***: select if the task is based on discriminating between stimulus rates, mutually exclusive with ***Side discrimination***
    a. ***Higher Rate Crct***: when checked, the higher of the presented rates signals lick Right
    b. ***2 Rates Only***: only present two rates
    c. ***Single panel***: when checked, only one panel / speaker presents stimulus, default is left side for a right hemisphere recording
    d. ***Mixed rates***: select for psychometric testing of rate discrimination, rates are calculated on a log scale between the ***High Rate (Hz)*** and ***Low Rate (Hz)*** defined below, actual rates are displayed in the ***Active Rates*** window to the right of the settings.
    e. ***No Test Punish***: when checked, suppresses punishment on light location that are not the training locations
    f. ***High Rate (Hz)***: defines the high boundary of stimulus rates
    g. ***Low Rate (Hz)***: defines the low boundary of stimulus rates
    h. ***Duration***: defines how long is the stimulus presented

Right panel

1. ***Input file***: Allow the upload of a predetermined sequence of left and right trails as detailed above.
2. ***Light Control***: buttons test the light panels
    a. ***Flash L***: this allows the user to check the Left (L) side LED panel.
    b. ***Flash C***: this allows the user to check the Center (C) side LED panel.
    c. ***Flash R***: this allows the user to check the Right (R) side LED panel.
    d. ***Light Sequence***: this allows the user to run all the LED panels going (L, C, R).
3. ***Tone Control***: buttons test the speakers
    a. ***L ON***: this allows the user to check the L side speaker.
    b. ***Vol +*** (under L ON): user can change the volume for L ON check
    c. ***R ON***: this allows the user to check the R side speaker.
    d. ***Vol +*** (under R ON): user can change the volume for R ON check
4. ***Valve Control***: buttons test the reward solenoids
    a. ***Reward buttons***: dispense one reward from respective spout
    b. ***Open Valve***: continuously open respective valve (eg. to fill or empty lines)
    c. ***Reward_pulses (ms)***: sets solenoid valve opening times for each spout

Center-bottom panel

1. ***Save Settings***: save all GUI parameters to file
2. ***Load Settings***: load previously saved GUI parameters from file
3. ***Buttons L, C, R***: mimic a lick to respective spout (for debugging)
4. ***Send Air Puff***: send an air puff of duration defined in ***General Controls***
5. ***Servo controls***
    a. ***Linear/Servo***: chose spout movement system
    b. ***Spout1 Rest***: determines extension of center spout at rest
    c. ***Spout1 Ext***: determines extension of center spout at full extension

d. ***Spout2 Rest***: determines extension of side spouts at rest
e. ***Spout2 Ext***: determines extension of side spouts at full extension
f. ***Full out***: sends all spouts to full extension (as determined above)
g. ***FullRetract***: sends all spouts to fully retracted position
h. ***ToRest***: sends all spouts to rest position
i. ***C Out***: only sends central spout out
j. ***LR Out***: only sends side spouts out

Detailed state machine for the two-alternative forced choice task (2AFC):

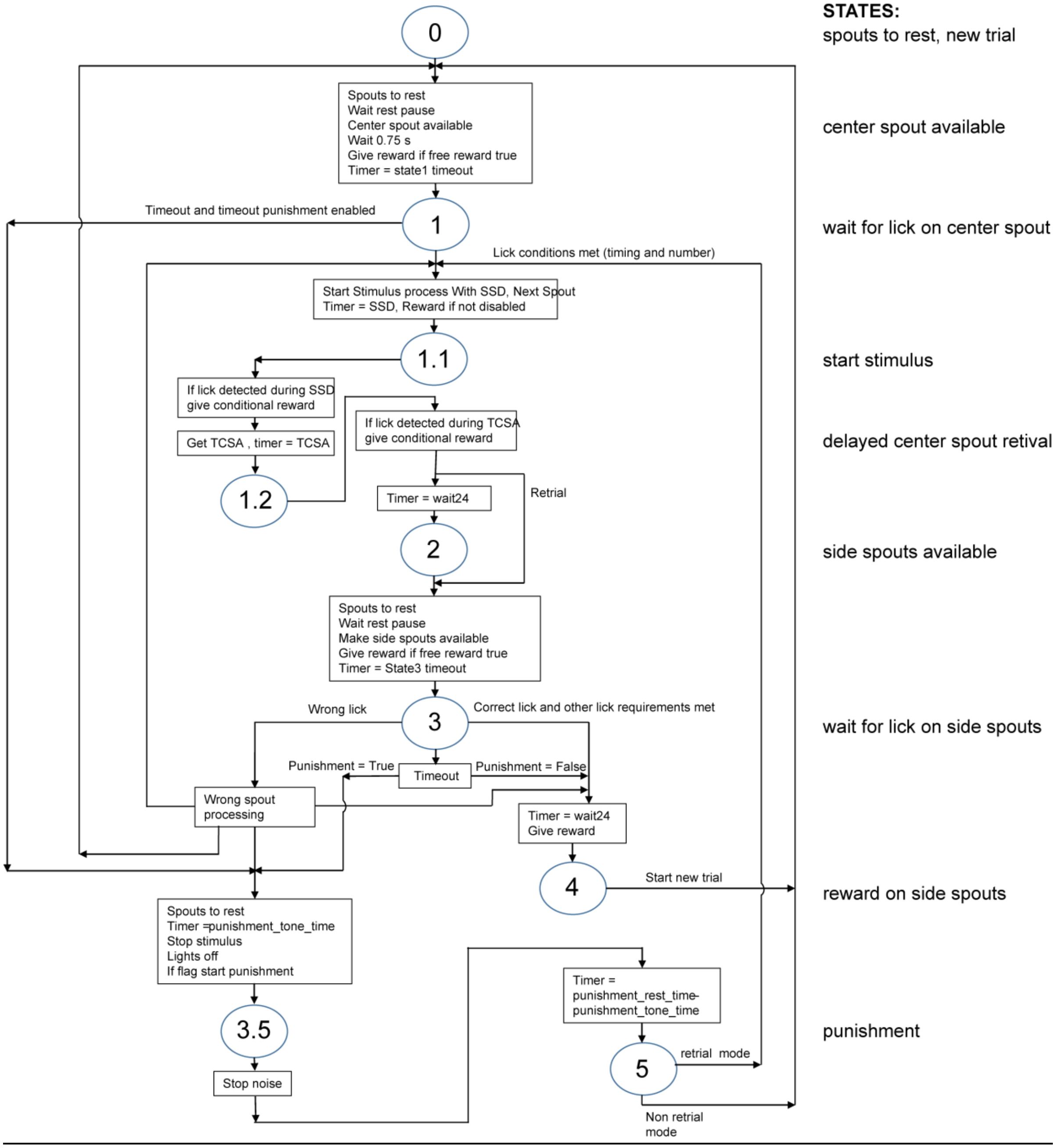

9. Go-nogo (GNG) graphical interface documentation

This GUI allows the user to customize stimuli and delays as well as check on a mouse's progress in real-time during a Go-NoGo task.

Control interface:

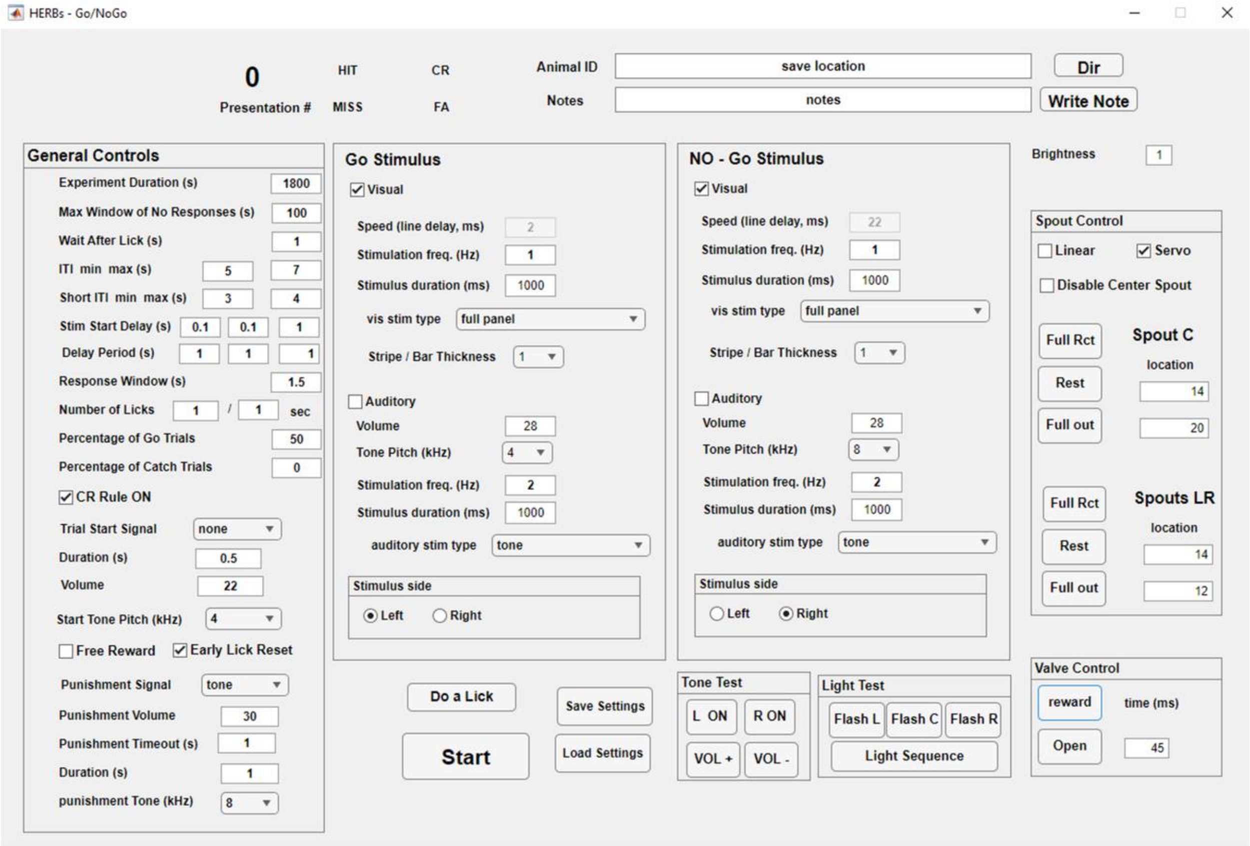

Top panel:

1. Presentation #, HIT, MISS, CR, FA. - These values are updated in-real time to inform the user of the mouse's progress.
    a. ***Presentation #***: displays the trial type (Go or NoGo) that the animal is currently viewing/listening
    b. ***HIT***: correct response to Go Trial: mouse licked spout
    c. ***MISS***: incorrect response to Go Trial: mouse refrained from licking the spout
    d. ***CR***: correct response to NoGo Trial: mouse refrained from licking spout
    e. ***FA***: incorrect response to Go Trial: mouse licked spout
2. ***Animal ID***: this can be a directory path to a saved data folder:
    a. Example: Z:\User\GNG\mouse1
3. ***Dir***: this allows the user to select the directory path to a saved data folder, and is used to prefill Animal ID (Item 2)
4. ***Notes*** bar: type in notes during the experiment
5. ***Write Note***: click to save timestamp for note, printed at the end of the experiment

Left Panel: General Controls

1. ***Experiment Duration(s)***: User inputs the length of the behavioral session in seconds.
    a. Example: A 5-minute session is 600s, so '600' would be typed.
2. ***Max Window of No Responses(s)***: The GUI stops the behavioral session if the mouse does not respond engage with the apparatus (i.e., does not perform a lick). User inputs the length of time corresponding to a maximum window of no responses before terminating the session.
3. ***Wait After Lick(s)***: User inputs how much time (in seconds) the mouse has to collect water reward.

   ITI (Intertrial Interval): the time (in seconds) in between trials. To have variable ITI lengths, the user can input two different values into the min and max. ITI and Short ITI can have the same values.
4. ***ITI min max(s)***: the ITI length (in seconds) which occurs after the mouse correctly responds to a GO trial (licks,"HIT"), incorrectly responds to a GO trial (withholds lick, "MISS"), or incorrectly responds to a NoGO trial (licks, "FA").
5. ***Short ITI min max(s)***: the ITI length (in seconds) which occurs after the mouse correctly responds to a NoGO trial (withholds licks,"CR").
6. ***Stim Start Delay(s)***: the time (in seconds) before the stimulus begins. To have variable delay periods before the stimulus appears, the user must input three values: a minimum (first box), a maximum (second box), and the number of "steps" (third box). The number of steps defined will define an equal distribution between min and max. Min and max values can be decimals. Min and max values will always be included if step is greater than 1. Step must equal 1 or higher, but if a decimal value is given, value will be rounded down. See some examples for how to set this up below under "Delay Period(s)".
7. ***Delay Period(s)***: the time (in seconds) between the end of the stimulus and the beginning of the response window. To have variable delay periods before the stimulus appears, the user must input three values: a minimum (first box), a maximum (second box), and the number of 'steps' (third box). The number of steps defined will define an equal distribution between min and max. Min and max values can be decimals. Min and max values will always be included if step is greater than 1. Step must equal 1 or higher, but if a decimal value is given, value will be rounded down.
    a. Example: Single, consistent delay period. min 0.2, max 0.2, step 0.5. MISTAKE: GUI will not function as step is not an integer
    b. Example: Single, consistent delay period. min 0.2, max 0.2, step 1. Delay period is always 0.2s
    c. Example: Single, consistent delay period. min 0.2, max 0.2, step 1.5. MISTAKE: GUI will round 1.5 to 1. Delay period is always 0.2s
    d. Example: Single, consistent delay period. min 1, max 1, step 1. Delay period is always 1s.
    e. Example: Variable delay period. min 0, max 2, step 1. MISTAKE: GUI will not give variable delays. Delay period is always 2s.
    f. Example: Variable delay period. min 0, max 2, step 2. Delay period can be either 0s or 2s.

    g. Example: Variable delay period. min 0, max 2, step 3. Delay period can be either 0s, 1s, 2s.
    h. Example: Variable delay period. min 0, max 2, step 4. Delay period can be either 0, 0.667s, 1.333s, 2s.
8. ***Response Window(s)***: the time (in seconds) the mouse has to respond to the presented stimulus (i.e. lick or withhold licking).
9. ***Number of Licks / sec***: the number of licks per second required to be counted as a correct response.
10. ***Percentage of Go Trials***: determines what proportion of the trials will be GO.
11. ***Percentage of Catch Trials***: determines the proportion of CATCH trials during the session. Catch Trials refer to the trials which do not display any stimulus.
12. ***CR Rule On***: Trial types are selected randomly, but an underlying rule is that a trial type will not repeat more than three times consecutively.
    a. Example: Go - Go - Go - NoGo - Go - Go -Go - NoGo.
    b. Example: NoGo - NoGo - NoGo - Go - NoGo - Go.
    c. Example:Go - NoGo - NoGo - Go.

    Selecting CR Rule On is critical for the extreme values (0% and 100%) of Percentage of Go Trials (Item 10). Selecting this allows for 0% Go trials to actually give 0 Go trials or 100% Go trials to actually give only Go trials. The CR Rule states that after a correct NoGo Trial (which results in a correct reject) a Go Trial will be given, aka forces a Go Trial. In other words, selecting CR Rule ON allows for a correct NoGo trial to be immediately followed by a Go Trial.
13. ***Trial Start Signal***: This is a drop down menu that allows the user to choose the stimulus (auditory/visual) to indicate that the trial has started. If none is chosen, then duration and volume are voided even if there are values in those boxes.
14. ***Duration(s)***: duration of the trial start signal.
15. ***Volume***: If an auditory start signal is chosen, then the user can also specify.
16. ***Start Tone Pitch (kHz)***: If an auditory start signal is chosen, then the user can also specify.
17. ***Free Reward***: This automatically dispenses a water reward during the presentation of a GO stimulus.
18. ***Early Lick Reset***: This restarts the ITI timer whenever the mouse licks outside of (so either before or after) the Response Window (Item 8). ITI timer continues to restart until mouse refrains from licking the spout.
19. ***Punishment Signal***: This is a drop down menu that allows the user to choose the stimulus (auditory/visual) to indicate that the wrong choice was made for a NoGo Trial (or a "FA"). This requires that Percentage of Go Trials (Item 10) be less than 100 (so to have a few NoGo trials). Otherwise, even if the option besides 'None' is selected for the Punishment Signal and Items 20-23 have values, there will be no punishment.
20. ***Punishment Volume***: If an auditory start signal is chosen, then the user can also specify.
21. ***Punishment Timeout***: In addition to the ITI, this window time is not reward if lick occurs.
22. ***Duration (s)***: This is the duration of the auditory or visual punishment which occurs concurrently with the punishment timeout.
23. ***Punishment Tone (kHz)***: If an auditory start signal is chosen, then the user can also specify.

Center panels: Go Stimulus/ NO- Go Stimulus

(This applies also to the NO-Go Stimulus panel as well)

The Go Stimulus and NO -Go Stimulus can be either both visual or both auditory or mixed.

1. ***Visual***: must be selected to be displayed. Values in Items 2 - 6 are voided if Visual is not selected.
2. ***Speed*** (line delay, ms): preset
3. ***Stimulation freq*** (Stimulation frequency) (Hz): User specifies an integer value for the number of presentations of the stimulus per second.
4. ***Stimulus duration (ms)***: User specifies an integer value the duration of stimulus presentation.
5. ***Vis Stim Type*** (Visual Stimulus Type): This is a drop-down menu that allows the user to choose the visual stimulus type: full panel, vertical stripes, horizontal stripes, left diagonal, right diagonal, single vertical stripe, single horizontal stripe.
6. ***Stripe/Bar Thickness***: This is a drop-down menu that allows the user to choose the stimulus thickness of the bars.
    a. Suggested: Stripe bar 1-2 is suggested for single and multiple vertical/horizontal bars.
7. ***Auditory***: must be selected to be displayed. Values in Items 8 - 11 are voided if Auditory is not selected.
8. ***Volume***: If an auditory start signal is chosen, then the user can also specify
9. ***Stimulation freq*** (Stimulation frequency) (Hz): User specifies an integer value for the number of presentations of the stimulus per second.
10. ***Stimulus duration*** (ms): User specifies an integer value the duration of stimulus presentation.
11. ***Auditory Stim Type*** (Visual Stimulus Type): This is a drop down menu that allows the user to choose the auditory stimulus type: tone, white noise.
12. ***Stimulus side***: Which side the stimulus is presented on. Go and NO -GO Stimulus can be both presented on the same side if both Left options are chosen or both Right options are chosen in the respective Go and NO-Go panels

Right panel

Buttons:

1. ***Brightness***: User specifies an integer value for the brightness of the visual stimulus, whether as Go signal, NO-GO signal, Trial Start Signal, or Punishment signal.

Spout Control

1. ***Linear/Servo***: spout movement system
2. ***Disable Center Spout***: disabling the movement of the Center Spout as Center spout moves after Delay Period (Left Panel:General Controls Item 7). When this is chosen, Full Rct/Rest/Full Out (Item 3-5) will not function.
    a. Suggested: Make sure that Spout is in Full Out (Item 5) position before enabling Disable Center Spout. This is useful during the beginning stages of Go/NOGo training so mice associate reward to stimulus rather than movement of the spout.

   The user has the option of using one spout ("Spout C"), two spouts ("Spouts LR") or three spouts ("Spout C" + "Spouts LR"). The following applies to both **Spout C** and **SpoutsLR**

3. ***Full Rct*** (Full retract): this places the spout at its furthest position from the mouse. Make sure Disable Center Spout (Item 2) is not chosen to check this, but after confirming this position, you can re-enable Disable Center Spout. (Item 2).
4. ***Rest***: this should be the position that spout remains in through Delay Period (Left Panel:General Controls Item 7). Mouse should not be able to lick spout when spout is at rest. Make sure Disable Center Spout (Item 2) is not chosen to check this, but after confirming this position, you can re-enable Disable Center Spout. (Item 2).
5. ***Full Out***: this should be the position that spout remains in until after Delay Period (Left Panel: General Controls Item 7). Mouse should be able to lick spout when spout is at Full Out but not at Rest. Make sure Disable Center Spout (Item 2) is not chosen to check this, but after confirming this position, you can re-enable Disable Center Spout. (Item 2).

Bottom panels:

Buttons:

1. ***Do a Lick***: GUI command interpreted as a lick from the animal
   a. Suggested use: trouble shooting. User can make a response (a “Lick”) to check GUI set-up.
2. ***Start/Stop***: This button appears as “Start” to initiate the trial but displays “Stop” during the trial. Clicking this button when it is labeled “Stop” will stop the trial.
3. ***Save Settings***: All GUI variables can be saved into a Matlab Data file. This file can be saved into a data folder specified by the user (similar to Animal ID, **Top panel** Item 4) and used in conjunction with Load Settings (Item 5).
4. ***Load Settings***: All GUI variables can be loaded from a Matlab Data file saved in a folder specified by the user if Save Settings was used (Item 3).

Tone Test

If an auditory stimulus was chosen for the following (whether for Trial Start Signal [Left Panel:General Controls Item 13], Punishment Signal [Left Panel:General Controls Item 19], or Go/No-GO stimulus [Center panels: Go Stimulus/ NO- Go Stimulus Item 7]), the following allows the user to check the auditory stimulus.

1. ***L ON***: this allows the user to check the L side speaker.
2. ***Vol +*** (under L ON): user can change the volume for L ON check
3. ***R ON***: this allows the user to check the R side speaker.
4. ***Vol +*** (under R ON): user can change the volume for R ON check

Light test

If an visual stimulus was chosen for the following (whether for Trial Start Signal [Left Panel:General Controls Item 13], Punishment Signal [Left Panel:General Controls Item 19], or Go/No-GO stimulus [Center panels: Go Stimulus/ NO- Go Stimulus Item 7]), the following allows the user to check the visual stimulus. The following also assumes that the user has a 3 LED panel system to display the visual stimulus.

1. ***Flash L***: this allows the user to check the Left (L) side LED panel.
2. ***Flash C***: this allows the user to check the Center (C) side LED panel.
3. ***Flash R***: this allows the user to check the Right (R) side LED panel.

4. ***Light Sequence***: this allows the user to run all the LED panels going from L to C to R.

Valve Control

During the GO/NoGO task, reward is dispensed via Free Reward (**Left Panel:General Controls,** Item 17) or during the trial when a "HIT" (the correct response [licking] to the Go trials) occurs.

1. ***Reward***: the user can dispense a reward manually.
    a. Suggested uses:
        i. Setting up the behavioral rig to make sure the reward system is working before starting the session.
        ii. Setting up the spout height and distance from the mouse's tongue.
        iii. Motivating mouse during the session
2. ***Open Valve "time (ms)"***: Depending on the spout system, this value indicates how long the solenoid valve will remain open to dispense water.
    a. Suggested: User should use a pipette to measure the amount of water dispensed per ms open.

Detailed state machine for the go-nogo task (GNG)

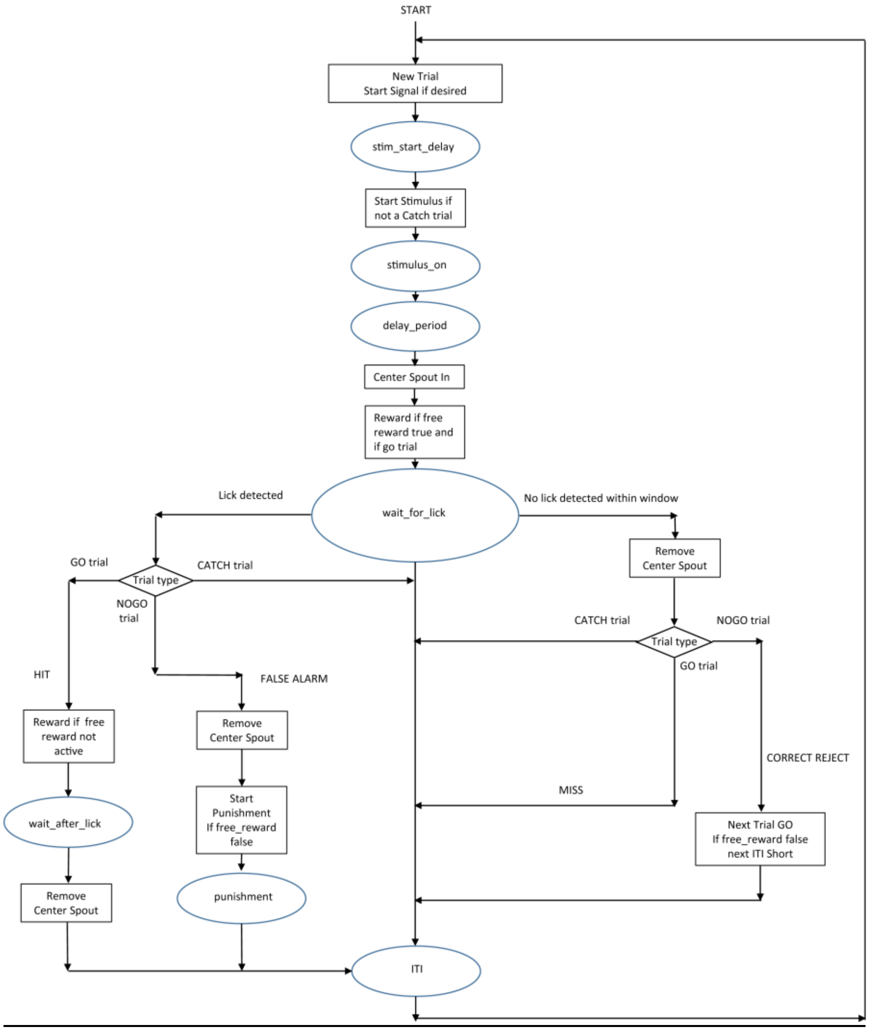

10. Sensory stimulus presentation graphical interface documentation

This GUI allows the user to customize stimuli and presentation intervals for sensory stimuli.
Control interface:

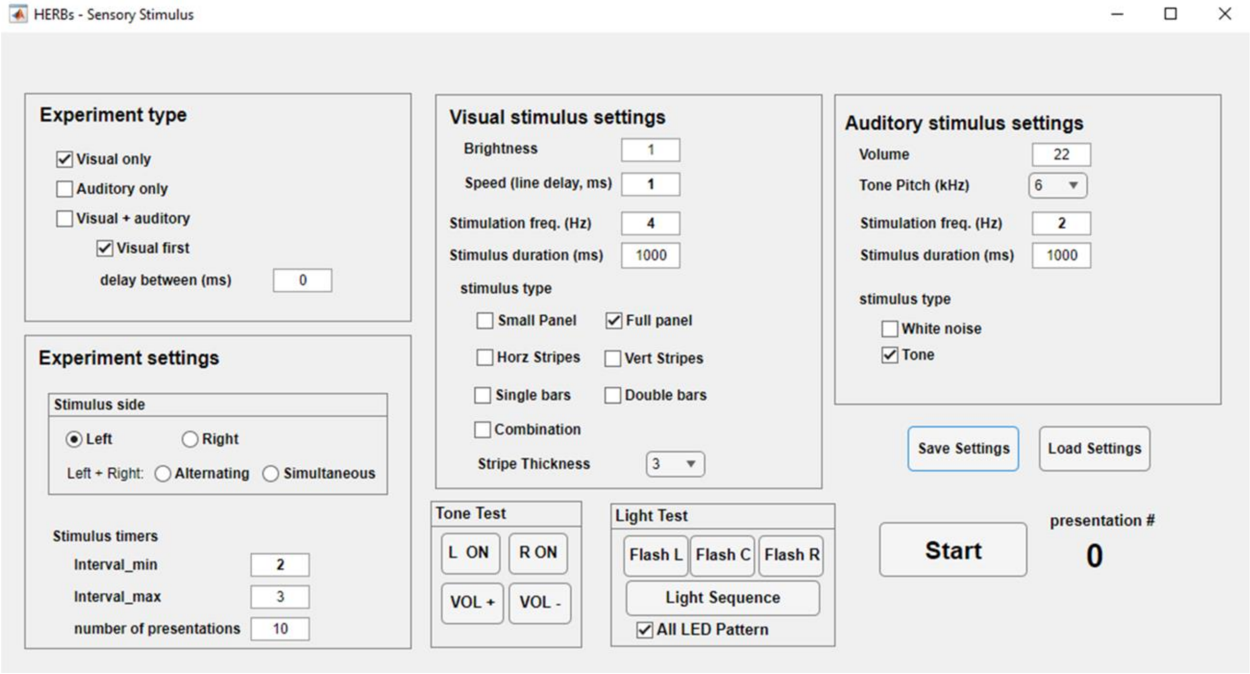

Main push buttons:

- ***Start***: Starts program with input settings
- ***Presentation #***: will update for each stimulus presentation
- ***Save Settings***: will save current settings as .txt for future input
- ***Load Settings***: will allow you to select a pre-saved settings file from previous session

Stimulus Test:

- ***Tone test***: plays a continuous tone with the frequency (tone pitch) as is selected in auditory stimulus settings (refer **F**)
  Note: volume adjustments for tone test should be made with these buttons instead of manual input in auditory stimulus settings (F)
- ***Light test***:
  i. ***Flash L/C/R***: flashes a subpart of selected panel; this function can be used to test that panels are responsive
  ii. ***All LED pattern:*** full panel will light up
  iii. ***Light Sequence***: displays bars in each panel sequentially starting with the left panel

Experiment Type: Select stimulus type

- ***Visual only:*** Refer to **E**
- ***Auditory Only:*** Refer to **F**
- ***Visual + auditory:*** option to present both visual and auditory stimuli, refer to settings for both **E** and **F,** note below if delay between stimulus types desired

i. ***visual first:*** note that if there is a delay and this tick box is not selected, will automatically play auditory stimulus first.
ii. ***Delay between:*** in milliseconds; if set to 0, will present visual and auditory stimuli simultaneously

Experiment settings:

- ***Stimulus side:*** Select either left/right/center (center for visual center panel)
  i. ***Alternating:*** Recommended to use even numbers so that both sides are presented the same number of times.
  Note: If using the Combination type visual stimulus, it is recommended to use in multiples of four, where each page in a set of four is one presentation.
  ii. ***Simultaneous*** (for visual only): Will present the stimuli on both the left and right side
  Note: visual, moving bars stimulus type selected, orientations will be top → bottom, bottom → top, inner → outer, outer → inner
- Stimulus timers:
  i. ***Interval min/max***: time in seconds for inter-trial intervals between presentations
  ii. ***Number of presentations***: number of times stimulus to be presented
  Note: if presenting visual stimulus, that this number should be set in such a way that the experimenter desires based on **visual stimulus type**

Visual Stimulus Settings

- ***Brightness***: brightness level of LED panels, recommended value 1-2
- ***Speed*** (line delay, ms): for moving lines if stimulus type single bars/double bars selected
- ***Stimulation frequency (Hz)***: presentation per second; "blinking" effect depending on stimulus duration
- ***Stimulus duration (ms):*** total stimulus presentation time in milliseconds
- ***Stimulus Type***
  i. Small panel: outer edge most 4x9 of panel
  ii. Full panel: entire panel
  iii. Horizontal stripes: horizontal stripe pattern across entire panel
  iv. Vertical stripes: vertical stripe pattern across entire panel
  v. Single bars: single-line moving bar in all four directions
  (ie., L →R, R→L, Top → bottom, or bottom → top in random order)
  Note: one set of **all** four directions will count as **one** presentation
  vi. Double bars: moving double-bar in all four directions
  vii. Combination: set of pages displaying vertical, horizontal, 45°, -45° bars of a selected bar thickness (only for combination) in random order
  Note: The pages are NOT moving, and **each** orientation counts as **one** presentation. Therefore, it is recommended to input number of presentations in multiples of four so that each orientation is presented an equal number of times.
- ***Stripe Thickness:*** select 1–3-line thickness for combination type

Auditory Stimulus Settings

- ***Volume***: up to 63. Calibration recommended.

- ***Tone Pitch (kHz):*** 2-32 kHz
- ***Stimulation frequency (Hz):*** number of beeps per second
- ***Stimulus duration (ms):*** for stimulus frequency
  i.e., frequency 5Hz, duration 1000ms will play 5 beeps (100 ms each) per second in one presentation
- ***Stimulus type:***
  i. Tone: pure tone based on selected tone pitch (kHz)
  ii. White noise: combination of waveforms that produce white noise

11. Parts List

	#	vendor	part #	link
Arduino				
Arduino mega	1	Arduino	A000067	https://store-usa.arduino.cc/products/arduino-mega-
Arduino DUE	1	Arduino	A000062	https://store-usa.arduino.cc/products/arduino-due
Electronics				
lick detector	3	sparkfun	14520	https://www.sparkfun.com/products/14520
capacitor	3	sparkfun	8375	https://www.sparkfun.com/products/8375
power control kit	3	sparkfun	12959	https://www.sparkfun.com/products/12959
MOSFET chip (alternative for power control)	3	sparkfun	10213	https://www.sparkfun.com/products/10213
level converter	1	sparkfun	BOB-12009	https://www.sparkfun.com/products/12009
diode rectifier	3	sparkfun	14884	https://www.sparkfun.com/products/14884
speakers	2	sparkfun	9151	https://www.sparkfun.com/products/9151
1/4-inch hub	1	sparkfun	12488	https://www.sparkfun.com/products/12488
LED panels	3	adafruit	2973	https://www.adafruit.com/product/2973
LED panel drivers	3	adafruit	2946	https://www.adafruit.com/product/2946
audio amp	1	adafruit	MAX9744	https://www.adafruit.com/product/1752
tubing (if longer range is needed)	1	Cole Parmer	EW-06419-04	https://www.coleparmer.com/i/masterflex-microbore
solenoid valve	3	Reet corp.	075P2NC12-02S	http://www.reetcorp.com/products-valves.html
1/4 inch shaft ~1.5'' long	1	McMaster	8632T144	https://www.mcmaster.com/8632T144/
1/4 bearings	2	McMaster	4262T36	https://www.mcmaster.com/4262T36/
1/4'' wheel lock shaft collar	1	McMaster	9946K11	https://www.mcmaster.com/9946K11/
12v power supply	1	amazon	generic	https://www.amazon.com/Adapter-SANSUN-AC100-2
9V power supply	1	amazon	generic	https://www.amazon.com/DAddario-PW-CT-9V-Powe
running wheel	1	amazon	generic	https://www.amazon.com/gp/product/B00ZB3S62K/r
plasti dip (wheel coating)	1	amazon	generic	https://www.amazon.com/Plasti-Dip-11252-6-Spray-F 7d4df261a850&pd_rd_w=Z2I6I&pd_rd_wg=YT3ET&pf
3D arm	1	amazon	generic	https://www.amazon.com/gp/product/B07KPY6ZC3/r
IR webcam	1	amazon	generic	https://www.amazon.com/gp/product/B0829HZ3Q7/

power strip (8x)	1	amazon	generic	https://www.amazon.com/Protector-Nuetsa-Extensio
PC	1	amazon	any	https://www.amazon.com/Dell-Inspiron-Compact-Tov
breadboards	1	amazon	generic	https://www.amazon.com/DEYUE-Solderless-Prototyp
20awg wire	1	amazon	generic	https://www.amazon.com/gp/product/B083DNGSPV/
goosneck lamp	1	amazon	generic	https://www.amazon.com/gp/product/B07L6T1N1K/r
spout tubing	1	amazon	generic	https://www.amazon.com/gp/product/B004WPQ59O
rotary servo	2	amazon	MG90S	https://www.amazon.com/gp/product/B07F7VJQL5/r
linear actuator (optional if not servo)	2	actuonix	L12-30-50-12-I	https://www.actuonix.com/Rod-Actuators-s/1924.htm
Mechanical parts				
90° holder	4	Thorlabs	RA90	https://www.thorlabs.com/thorproduct.cfm?partnum
adjustable angle holder	2	Thorlabs	SWC	https://www.thorlabs.com/thorproduct.cfm?partnum
1/2'' rod to screw	1	Thorlabs	RA180	https://www.thorlabs.com/thorproduct.cfm?partnum
1/2'' rod, 1''	1	Thorlabs	TR1	https://www.thorlabs.com/newgrouppage9.cfm?obje
1/2'' rod, 2''	1	Thorlabs	TR2	https://www.thorlabs.com/newgrouppage9.cfm?obje
1/2'' rod, 4''	3	Thorlabs	TR4	https://www.thorlabs.com/newgrouppage9.cfm?obje
1/2'' rod, 6''	4	Thorlabs	TR6	https://www.thorlabs.com/newgrouppage9.cfm?obje
1/2'' rod, 8''	1	Thorlabs	TR8	https://www.thorlabs.com/newgrouppage9.cfm?obje
post holder base	4	Thorlabs	BA1	https://www.thorlabs.com/newgrouppage9.cfm?obje
post holder base	2	Thorlabs	BA1S	https://www.thorlabs.com/newgrouppage9.cfm?obje
post holders 1’’	1	Thorlabs	PH1	https://www.thorlabs.com/newgrouppage9.cfm?obje
post holders 2’’	2	Thorlabs	PH2	https://www.thorlabs.com/newgrouppage9.cfm?obje
post holders 3’’	2	Thorlabs	PH3	https://www.thorlabs.com/newgrouppage9.cfm?obje
mini adapter	2	Thorlabs	MSA8	https://www.thorlabs.com/newgrouppage9.cfm?obje
90° bracket	2	Thorlabs	ER90B	https://www.thorlabs.com/newgrouppage9.cfm?obje
cage rod	2	Thorlabs	ER05	https://www.thorlabs.com/newgrouppage9.cfm?obje
3D stage	1	Thorlabs	DT12XYZ	https://www.thorlabs.com/newgrouppage9.cfm?obje
breadboard	1	Thorlabs	MB1218	https://www.thorlabs.com/newgrouppage9.cfm?obje
machined / printed				
LED panel holder	1			
spout holder	1			
servo-to-linear-actuator translator	1	lab made 3D print		
data acquisition				
NI DAQ (alternative)	1	National instruments	USB-6218	https://www.ni.com/en-us/shop/hardware/products/
MC USB-1208FS-Plus (low cost)	1	Measurement Computing	6069-410-061	https://www.mccdaq.com/usb-data-acquisition/USB-[illegible]

enclosure				
1 3/8'' dowel for frame 12'' long	8			
1 3/8'' dowel for frame 24'' long	6			
1 3/8'' dowel for frame 18'' long	6			
1 3/8'' dowel 8ft cut to above sizes	5	Home Depot	generic	https://www.homedepot.com/p/Mendocino-Forest-P
1/2'' plywood 21x24'' for tops				
1/2'' plywood 14x11'' for doors				
1/2'' plywood 30x24'' for back				
1/2'' plywood 30x21'' for sides				
1/2'' plywood 4' x 8' cut to above size	1	Home Depot	generic	https://www.homedepot.com/p/Columbia-Forest-Pro
door hinges	8	amazon	generic	https://www.amazon.com/LIBERTY-H01915C-SN-2-Inc Overlay/dp/B005YVKMR6/ref=sr_1_6?crid=1UZLTQ5JI -6
door magnets (set of 4)	2	amazon	generic	https://www.amazon.com/JQK-Magnetic-Stainless-Th 8c368726ac8e&pd_rd_w=EKVF9&pd_rd_wg=opalB&p
2'' wood screws (box)	1	amazon	generic	https://www.amazon.com/gp/product/B08MDXC1GG
0.5'' truss head wood screws (box)	1	amazon	generic	https://www.amazon.com/gp/product/B08545BSYB/r
egg crate foam	1	amazon	generic	https://www.amazon.com/Polyurethane-Charcoal-Co
mass loaded vinyl	2	amazon	generic	https://www.amazon.com/TMS-Sound-Proofing-Padd ff6136f9ad59&pd_rd_w=5qHAU&pd_rd_wg=Vc3wQ&

12. Installation instructions

1. Build hardware and electronic components (see further instructions in the Extended Data)
2. Upload scripts to Arduino MEGA
3. Upload scripts to Arduino DUE
4. In MATLAB cd to the folder containing all HERBs code (launching the GUI will automatically add the relevant folders to your path) and type in the command line the following to launch:
    a. for 2AFC type >> `HERBs_2AFC`
    b. for GNG type >> `HERBs_GoNoGo`
    c. for passive perception type >> `HERBs_Sensory_Stimulus`
5. To launch applications without MATLAB installation, first install Runtime then launch the appropriate .exe file (see more instructions in Section 1 of this document).
